# Willingness to accept the HBV vaccine and related factors: Administering the vaccination attitudes examination scale to Urban Vietnamese adults

**DOI:** 10.1371/journal.pgph.0006886

**Published:** 2026-07-22

**Authors:** Nghi Hoc Ho, Tung Hoang, Cong Minh Le, Tram Thi Huyen Nguyen

**Affiliations:** 1 Faculty of Pharmacy, University of Health Sciences, Vietnam National University, Ho Chi Minh City, Vietnam; 2 Institute for Social Sciences and Humanities Research, University of Social Sciences and Humanities, Vietnam National University Ho Chi Minh City, Ho Chi Minh City, Vietnam; University of Antwerp, BELGIUM

## Abstract

Vietnam has low hepatitis B vaccination coverage among adults, posing a threat to the healthcare system. To mitigate negative impacts, it is essential to identify vaccine hesitancy and refusal levels and their sociodemographic correlates. We conducted a cross-sectional survey of 926 adults in Ho Chi Minh City (January–March 2025) using a self-administered questionnaire and the Vaccination Attitudes Examination (VAX) scale. Descriptive analysis was performed, and associations between variables were explored using a chi-square test. Multinomial logistic regression identified correlates of vaccine hesitancy and refusal, statistical significance was set at p < 0.05. When analyzing general vaccine intentions regardless of vaccination status, 65.6% of participants indicated acceptance, 19.3% hesitancy, and 15.1% refusal. The overall Cronbach’s alpha was 0.833 (range = 0.812–0.919), and the McDonald’s omega coefficients ranged from 0.820 to 0.920. The uniqueness values (0.115–0.422) indicated that substantial variances were explained by latent factors, with significant unstandardized loadings of 0.94 to 1.31 (*p* < 0.001). A unique “knowledge paradox” was identified: poor knowledge about the HBV vaccine predicted outright refusal (AOR = 2.42), whereas higher factual knowledge was positively correlated with mistrust of vaccine benefits (r = 0.23, p < 0.01), worries about future effects (r = 0.19, p < 0.01), and total VAX scores (r = 0.19, p < 0.01). This suggests that limited knowledge contribute to refusal, while greater factual knowledge coexisting with “informed skepticism” or cautious hesitancy. Vaccine refusal was primarily associated with a preference for natural immunity, while mistrust of vaccine benefits and commercial concerns were specifically linked to increased hesitancy. The VAX scale serves as a reliable instrument for assessing vaccine attitudes among urban Vietnamese adults. It helps identify key vaccine-related concerns and supports continuous monitoring of public attitudes, which is essential for designing effective vaccination programs and enhancing public trust in vaccines.

## Introduction

Hepatitis remains one of the most serious public health threats of the 21^st^ century due to its severe long-term complications, such as liver cirrhosis, liver failure, liver cancer, and hepatocellular carcinoma, a problem that has prompted extensive global efforts to control and eliminate the disease [1]. Globally, approximately 254 million individuals were afflicted with hepatitis B virus (HBV), which was responsible for an estimated 1.1 million deaths, accounting for 83% of all viral hepatitis-related fatalities. The burden of chronic hepatitis B and C is highest among individuals aged 30–54 years, who represent half of all cases, while children under 18 years account for 12%. Men constitute 58% of the total infected population [1]. Moreover, even if the incidence of HBV infections has decreased globally, geographic disparities still exist. For example, the WHO stated that African and Southeast Asian regions are particularly affected, with these areas suffering from a 3% prevalence of the disease as well as bearing the burden of 60.5 million afflicted people and 180,000 HBV-related fatalities in 2019 [2].

The WHO Global Health Sector Strategy on Viral Hepatitis aims to eliminate viral hepatitis as a public health threat by 2030, targeting a 90% reduction in incidence and a 65% reduction in mortality [3]. However, achieving these goals remains particularly challenging for developing countries. Vietnam, a middle-income nation in Southeast Asia with a population of 97 million, has an HBV prevalence of 7% to 8% [4], ranking 9^th^ globally in terms of disease burden. In the country, as well, HBV infection is the leading cause of liver cancer [5], with chronic hepatitis B accounting for up to 62.3% of cases at Cho Ray Hospital in Ho Chi Minh City [6].

Vaccination remains a key intervention in eliminating the HBV infection, consistent with the WHO’s 2030 global elimination target. The hepatitis B vaccine is a safe and effective method of preventing HBV infection, with a birth dose and subsequent boosters providing nearly 100% protection [7]. Advancements in vaccine development also promise to curb HBV transmission by reducing mother-to-child transmission and enhancing vaccine immunogenicity in at-risk adult groups [8]. A one-year interruption in birth-dose and childhood HBV vaccination could cause 5.3 million additional chronic infections from 2020 to 2030, leading to 1 million more HBV-related deaths [7]. Vietnam has used HBV vaccination as part of its national immunization program since 2003, achieving the WHO target of 90% coverage for three doses and 50% coverage for birth doses by 2020 [9]. Nonetheless, vaccination rates among adults, those born before the implementation of the National Expanded Program on Immunization for children, remain low. This is largely due to Vietnam’s National Action Plan for the Prevention and Control of Hepatitis B (2021–2025), which prioritizes children and high-risk groups rather than the broader adult population [10].

Meanwhile, vaccine hesitancy, defined by the SAGE Working Group as a delay or refusal to acquire vaccination despite the availability of services, has been listed by WHO as a top 10 global health threat since 2019 [11]. Such reluctance diminishes immunization rates, increasing the susceptibility of individuals and weakening herd immunity, which in turn, raise outbreak risks and may reverse progress against vaccine-preventable diseases [12]. Vaccine hesitancy has increased since the 2009 influenza pandemic, as suggested in the literature [12]. However, the issue gained much greater attention and urgency during the COVID-19 pandemic, which caused widespread disruptions including prolonged lockdowns, increased misinformation, and significant interruptions to routine immunization services worldwide [13]. These factors have intensified the complexity of vaccine hesitancy, which is widely recognized as a complex and context-dependent issue, with attitudes ranging from full acceptance to outright rejection of vaccines. Notably, the reluctance to get vaccinated can occur even among individuals who acknowledge the importance of immunization [14]. This behavior is driven by several factors, including socioeconomic conditions, parental education, maternal healthcare access, and lack of knowledge. In the Vietnamese context, while the National Expanded Program on Immunization has provided free HBV vaccines for children since 2003, the National Action Plan for 2021–2025 does not prioritize the broader adult population. Consequently, most adults must access vaccination through voluntary services and pay out-of-pocket expenses. This financial requirement makes income a major determinant of vaccine uptake, as individuals with lower economic status may face significant barriers in accessing non-subsidized immunization services. Furthermore, specific cultural factors in Vietnam, such as the widespread usage of Traditional Medicine and a long-standing reliance on natural remedies, may significantly shape public preferences for natural immunity over pharmaceutical interventions [15]. Broader influences encompass awareness, past experiences, perceived vaccination importance, social norms, religious or moral beliefs, distrust in vaccine manufacturers, and concerns regarding risk [14,16]. Understanding the intricate interplay between the factors affecting vaccine acceptance is essential for public health practitioners, policymakers, and researchers to be able to optimize vaccination coverage and improve immunization strategies.

Such aims can be realized through the vaccination attitudes examination (VAX) scale, a robust instrument introduced by Martin and Petrie in 2017 to evaluate attitudes toward vaccination [17]. The scale has undergone thorough psychometric evaluation across various countries and has been translated into multiple languages, including Spanish, Romanian, Hebrew, Turkish, French, and Colombian [18–23]. Meanwhile, vaccine acceptance is largely influenced by the interplay between perceived disease risk and vaccine safety [24]. Individuals often prioritize avoiding disease exposure over vaccination, particularly when personal risk is deemed low. Acceptance also varies across nations and cultural contexts, shaped by psychosocial factors and differing views on the advantages of vaccines [25]. These challenges highlight the importance of reliable psychometric tools, such as the VAX scale, in effectively assessing hesitancy-related factors. The VAX scale is designed to measure general attitudes towards vaccination rather than hesitancy related to a specific type of vaccine. This makes the VAX scale a versatile and valuable tool for assessing vaccine attitudes in diverse contexts, including during the COVID-19 pandemic.

On the basis of the studies discussed above, the current research was conducted to bridge the tremendous gap in our understanding of vaccination attitudes, specifically the degree of hesitancy and refusal toward the HBV vaccine and the independent factors influencing these behaviors among Vietnamese adults. By offering evidence-based insights to create focused interventions that successfully address vaccine hesitancy, the findings are anticipated to influence public health programming and initiatives. Furthermore, this study adds to the creation of a reliable and validated measure for assessing general vaccine attitudes, which may be used to assist continuous monitoring and targeted communication efforts to increase vaccination uptake in the Vietnamese population.

## Materials and methods

### Ethics statement

In this research, informed consent was secured from all participants, all of whom were aged 18 years and above. Before giving their agreement, participants were fully aware of the study’s goals, methods, possible dangers, and benefits. Written consent was acquired and appropriately recorded. The institutional ethics committee examined and approved the study protocol, which included the consent procedure.

### Setting and research design

This study was conducted from January to March 2025. A cross-sectional design was implemented following the STROBE (Strengthening the Reporting of Observational Studies in Epidemiology) reporting guidelines.

Respondents were recruited through convenience sampling based on the following criteria: individuals aged 18 years or older, Vietnamese citizens, permanent residents of Ho Chi Minh City, able to understand the questionnaires, and willing to participate in the study. The selection process did not intentionally target any specific group, but individuals who were illiterate, were unable to understand Vietnamese, or provided incomplete survey responses were excluded from the analysis.

The required sample size was determined using the formula $\frac{Z_{\text{1}-\alpha /\text{2}}^{\text{2}}p(\text{1}-p)}{d^{\text{2}}}$, where the standard normal variate ($Z_{\text{1}-\alpha /\text{2}}$) is 1.96 at a 95% confidence level (α = 0.05), the expected population proportion (p) is 0.5, and the margin of error (*d*) is 5% [5]. On the basis of this calculation, a minimum of 384 respondents was required to achieve adequate statistical power. To account for an anticipated 10% of incomplete responses, the target sample size was adjusted to 423, which was further expanded to 1,100 respondents to reduce sampling error.

### Data collection and instruments

A structured self-administered questionnaire was developed on the basis of similar surveys and the relevant literature on the topic [17,26,27] before it was modified to align with the study’s objectives. The questions were refined to eliminate ambiguity, ensuring clarity and conciseness. The questionnaire demonstrated strong internal consistency, with a Cronbach’s alpha (α) coefficient of 0.897. Following revisions, it exhibited good reliability and validity.

The final questionnaire consisted of three sections. The first encompassed five questions regarding sociodemographic characteristics, such age, gender, personal income, occupation, and vaccination status. The second section consisted of 10 questions about specific knowledge of HBV vaccines explicitly stating that the questions are neutral and aim to assess health literacy without bias or leading content, and the final section comprised 12 questions covering vaccination intention and those in the VAX scale.

Data collection was conducted from January to March 2025 using either Google Forms or printed questionnaires. Our research collaborators approached individuals in public spaces, such as parks, cafés, and supermarkets, to distribute the questionnaire. Participation was voluntary, with informed consent forms acquired before proceeding with the survey. We maintained anonymity and confidentiality by refraining from collecting personally identifiable information. The respondents were told that they were free to withdraw at any time, but no incentives were offered to ensure an unbiased and diverse sample covering individuals of different socioeconomic backgrounds.

### Questionnaire

#### Sociodemographic characteristics.

The survey took on average of 20 minutes to complete. The following basic demographic details were derived: age (18–44 years, ≥ 45 years), gender (coded as 1 = male, 2 = female), personal income (in VND, with average monthly income categorized as follows: < VND 15 million (<US$ 605) and ≥VND 15 million (≥US$ 605), occupation (with the following codes used: 1 = manual labor, 2 = nonmedical intellectual labor, 3 = healthcare worker, 4 = homemaker/retired/unemployed), vaccination status (coded as 1 = unvaccinated, 2 = vaccinated), and vaccine intention (coded as 1 = refused, 2 = hesitant, 3 = accepted).

#### Knowledge of the HBV vaccine.

Ten items in the vaccine knowledge section were equally weighted and scored dichotomously. Each question had the response options “yes,’ “no,” and “do not know.” The total knowledge score was calculated by summing the number of correct answers, with the maximum possible score being 10. The respondents scoring 5 or higher were classified as having good knowledge of the HBV vaccine, while those scoring below 5 were considered to have poor knowledge [28,29].

#### Vaccination intention.

A six-point Likert scale was used to assess the respondents’ intention to receive the HBV vaccine. They were asked to answer the question “How do you rate your chances of getting the HBV vaccine?” The scale ranged from 1 to 6, with ratings of 1–2 indicating unwillingness or refusal, 3–4 reflecting hesitancy, and 5–6 representing a strong likelihood of vaccine acceptance.

#### Vaccination attitudes examination scale.

The VAX scale [17] consists of 12 items intended to measure general attitudes toward vaccinations. It comprises four three-item subscales: (1) mistrust of vaccine benefits, (2) worries about unforeseen future effects, (3) concerns about commercial profiteering, and (4) preference for natural immunity. The respondents were asked to rate their agreement with each item using a six-point scale ranging from 1 (*totally disagree*) to 6 (*totally agree*). The level of negative attitudes toward vaccination across all four VAX domains was arranged into three groups: low (1–2), intermediate (3–4), and high (5–6). The final score was obtained by calculating the mean scores for each subscale as well as the mean total score. Low scores indicated positive vaccination views, whereas high scores represented negative perspectives [17].

### Data processing and statistical analyses

#### Data processing.

The data processing was grounded in a structured approach to ensure accuracy and reliability. The dataset was cleaned by removing missing values and incomplete responses using a listwise deletion approach (complete case analysis). This method ensured that only respondents with complete data across all variables were included in the final analysis (n = 926), while maintaining a sample size that exceeded the minimum requirement for statistical power. For surveys that were completed using printed questionnaires, data entry was conducted manually by trained research assistants, followed by double-checking and verification to minimize entry errors and ensure data quality. Outliers were then identified and excluded to prevent bias in the analysis by using a box plot and the Shapiro–Wilk test.

#### Statistical analyses.

Data were analyzed using IBM Statistical Package for the Social Sciences (SPSS) for Windows, version 26.0 (IBM Corp., Armonk, NY, USA), AMOS version 24.0, and the ‘psych’ package in R version 4.3.1 (R Foundation for Statistical Computing). Continuous variables were reported as means with standard deviations (*SD*s), while categorical variables were presented as frequencies and percentages. Chi-square (χ²) tests were carried out to examine differences in vaccine uptake across categorical variables, with statistical significance set at *p* < 0.05. Additionally, multinomial analyses were performed to identify the independent factors associated with vaccine hesitancy and refusal.

Confirmatory factor analysis (CFA) was performed using maximum likelihood parameter estimates with standard errors and mean-adjusted chi-square tests to validate the structural model. Model fit was evaluated on the basis of several indices with established cut-off criteria: chi-square test, comparative fit index (CFI), Tucker–Lewis index (TLI), root mean square error of approximation (RMSEA), and standardized root mean square residual (SRMR). The internal reliability of the VAX scale was assessed using Cronbach’s α and McDonald’s omega (ω) coefficients. Measurement invariance across genders (male vs. female) was tested via three nested models with increasing constraints: configural invariance, metric invariance, and scalar invariance. Measurement invariance was confirmed when the change in CFI (ΔCFI) remained below 0.01 [30].

We used multinomial logistic regression to estimate adjusted odds ratios (AOR) and 95% confidence intervals for hesitancy and refusal versus acceptance. Multinomial logistic regression was performed to identify the correlates of vaccine hesitancy and refusal. Vaccine acceptance was used as the reference outcome. The reference category for gender was female; for age, 45 years or older; for occupation, homemaker/retired/unemployed; for income, at least 605 USD per month; for vaccination status, not yet vaccinated; for HBV knowledge, good knowledge; and for VAX scores, the low VAX score group.The variables considered theoretically important, including demographic characteristics (e.g., age, sex, and socioeconomic status) and interpersonal factors (e.g., knowledge of hepatitis B vaccination), were entered into the model. Statistical significance was assessed using two-tailed tests, with *p* < 0.05 considered indicative of a statistically significant association.

### Risk of bias assessment

Quality assessment was performed using the STROBE checklist, which is designed to evaluate the quality and transparency of observational studies. The checklist includes key items related to study design, participants, variables, biases, statistical methods, results, and interpretations [31] (**S1 STROBE Checklist**).

## Results

### Demographic characteristics of the participants

The sociodemographic characteristics of the participants are summarized in **Table 1**. There were a total of 926 participants, among whom 49.6% were male (*n* = 459), 35.3% (*n* = 327) were 45 years or older, and over half (64.7%, *n* = 599) were aged 18–44 years. More than 70% of participants (n = 676) reported a monthly income of less than US$605. This threshold reflects the average urban living standard in Ho Chi Minh City, serving as a benchmark to distinguish between low- and middle-to-high income levels in this specific economic hub. Occupationally, 42.3% (*n* = 392) were nonmedical intellectual laborers, 34.8% (*n* = 322) were manual laborers, 11.6% (*n* = 107) were healthcare workers, and 11.3% (*n* = 105) were homemakers/retired/unemployed. Among the participants, 41.4% (*n* = 383) were unvaccinated, whereas 58.6% (*n* = 543) had received the HBV vaccine. Regarding vaccination status, 58.6% (n = 543) of participants had been vaccinated, while 41.4% (n = 383) had not. When analyzing vaccination intentions, acceptance was 65.6%, hesitancy 19.3%, and refusal 15.1%.

**Table 1:** Sociodemographic characteristics of the study population (*N* = 926).

Variable	Number	Percentage (%)
**Gender**		
Male	459	49.6
Female	467	50.4
**Age (years)**		
18-44	599	64.7
≥ 45	327	35.3
**Occupation**		
Manual labor	322	34.8
Non-medical intellectual labor	392	42.3
Healthcare worker	107	11.6
Homemaker/Retired/Unemployed	105	11.3
**Income (USD/month)**		
< 605	676	73.0
≥ 605	250	27.0
**Vaccination status**		
Vaccinated	543	58.6
Not yet	383	41.4
**Vaccination intention among unvaccinated participants (n = 383)**		
Intent to vaccinate	275	71.8
No intention	108	28.2
**General vaccine intentions regardless of vaccination status (n = 926)**		
Accept	607	65.6
Refusal	140	15.1
Hesitant	179	19.3

US$1 = VND 24,786 (*source:* Vietnamese Ministry of Finance: exchange rate for foreign currencies in March 2025)

Table 2 presents respondents’ knowledge about the Hepatitis B (HBV) vaccine, including its effectiveness, dosing, duration of protection, necessity of post-vaccination testing, and safety. The data indicate high awareness in certain areas, with 86.4% of participants correctly identifying that an effective vaccine exists (K1) and knowing where to get vaccinated (K10). Additionally, a majority (80.2%) recognized that the vaccine is safe (K9), and 75.2% understood that a complete dose is 95% effective (K3).

**Table 2:** Knowledge about HBV vaccine (n = 926).

Items	Variables	Correct response, n (%)	Incorrect response, n (%)
K1	There is effective vaccine for hepatitis B	800 (86.4)	126 (13.6)
K2	Hepatitis B vaccine dosing 0, 1, 6 months: 3 doses	679 (73.3)	247 (26.7)
K3	Complete dose of Hepatitis B vaccine is 95% effective	696 (75.2)	230 (24.8)
K4	It provides protection for at least 20 years	647 (69.9)	279 (30.1)
K5	A patient who has fully recovered cannot infect others	586 (63.3)	340 (36.7)
K6	Post-Hepatitis B vaccination test is necessary	680 (73.4)	246 (26.6)
K7	For protection against Hepatitis B, one needs antibody titre of >10 IU/m	652 (70.4)	274 (29.6)
K8	The HBV vaccine may cause harmful side effects for recipients	633 (68.4)	293 (31.6)
K9	The HBV vaccine is safe for recipients	743 (80.2)	183 (19.8)
K10	You know where to get the HBV vaccination	800 (86.4)	126 (13.6)

However, while knowledge about HBV vaccine efficacy and accessibility is good, misconceptions about transmission risks and vaccine safety remain. Only 63.3% correctly answered that a fully recovered patient cannot infect others (K5), suggesting uncertainty about HBV transmission. Furthermore, 31.6% of respondents believed that the HBV vaccine may not cause harmful side effects (K8), highlighting safety concerns that could impact vaccine acceptance.

Table 3 presents the VAX scale data. As previously stated, the mean scores ranged from 3.14 (Item 9) to 4.49 (Item 1). The internal dependability of the majority of the items was indicated by the adjusted item-total correlations exceeding 0.40. Items V1, V2, and V3 contributed less to the scale, as their correlations fell below this threshold.

**Table 3:** Descriptive statistics of the VAX scale.

Item	Min	Max	Mean	Std. Deviation	Skewness	Kurtosis	Item-total correlation
V1	1	6	4.49	1.04	−1.02	0.77	0.37
V2	1	6	4.43	0.99	−0.97	0.83	0.38
V3	1	6	4.39	1.02	−0.87	0.44	0.36
V4	1	6	4.35	1.04	−0.81	0.25	0.40
V5	1	6	4.33	1.07	−0.82	0.23	0.46
V6	1	6	4.29	1.05	−0.73	0.04	0.47
V7	1	6	3.29	1.43	−0.14	−1.10	0.53
V8	1	6	3.24	1.45	−0.11	−1.17	0.55
V9	1	6	3.14	1.50	−0.02	−1.27	0.50
V10	1	6	3.80	1.31	−0.58	−0.63	0.62
V11	1	6	3.67	1.38	−0.50	−0.87	0.63
V12	1	6	3.67	1.41	−0.52	−0.90	0.61

### Preliminary analysis

Model fit was significant (χ²(32) = 287.40, p < 0.001), indicating that the final model provided a better fit than the intercept-only model. Goodness-of-fit tests showed an adequate fit (χ²(904) = 818.02, p = 0.981), although Pearson χ² was significant. Pseudo-R^2^ values suggested moderate explanatory power (Cox & Snell = 0.267; Nagelkerke = 0.322; McFadden = 0.176). Likelihood ratio tests indicated that age, occupation, income, vaccination status, HBV knowledge, and three VAX subscales (F1, F3, F4) significantly contributed to the model (all p < 0.05). Multicollinearity was examined using Variance Inflation Factors (VIF), all predictors showed VIF values ranging from 1.34 to 2.10, these results indicate that multicollinearity was not a concern in the regression model.

Parallel factor analysis confirmed the validity of the four-factor structure of the VAX scale, with a strong fit (χ²(24, *N* = 926) at 41.61, a *p* = 0.01, a TLI = 0.99, and an RMSEA = 0.028 (95% confidence interval [CI]: 0.01–0.04). The model explained 70.04% of the cumulative variance in the data. A KMO = 0.85 indicated sampling adequacy, and Bartlett’s test showed substantial item correlations (χ²(66, *N* = 926) = 7050.23, *p* < .001), permitting component analysis (see Table 4).

**Table 4:** Factor loadings, explained variances, and reliability.

Items	F1	F2	F3	F4	Uniqueness (PAF)
V1	−0.010				0.419
V2	−0.012				0.284
V3	0.034				0.315
V4		0.318			0.414
V5		0.090			0.345
V6		−0.106			0.419
V7			−0.053		0.209
V8			0.012		0.190
V9			0.073		0.207
V10				0.159	0.422
V11				−0.058	0.115
V12				−0.058	0.213
Cronbach’s alpha (α)	0.848	0.812	0.919	0.890	
McDonald’s omega (ω)	0.850	0.820	0.920	0.900	

F1  =  mistrust of vaccine benefit; F2  =  worries about unforeseen future effects; F3  =  concerns about commercial profiteering; F4  =  preference for natural immunity.

The CFA based on the maximum likelihood χ² S-B estimator for nonnormal data validated the VAX scale’s four-component structure. The results suggested a strong fit—χ²(49, *N* = 926) = 198.454, *p* < 0.001, CFI = 0.98, TLI = 0.97, RMSEA = 0.06 (90% CI: 0.05–0.07); internal consistency was satisfactory, with Cronbach’s α ranging from 0.812 to 0.919 and McDonald’s ω ranging from 0.820 to 0.920 across subscales, demonstrating satisfactory internal consistency. Most of the items significantly contributed to latent components, but those with high uniqueness values (>0.40) pointed to weaker correlations or ambiguity.

**Fig 1:**
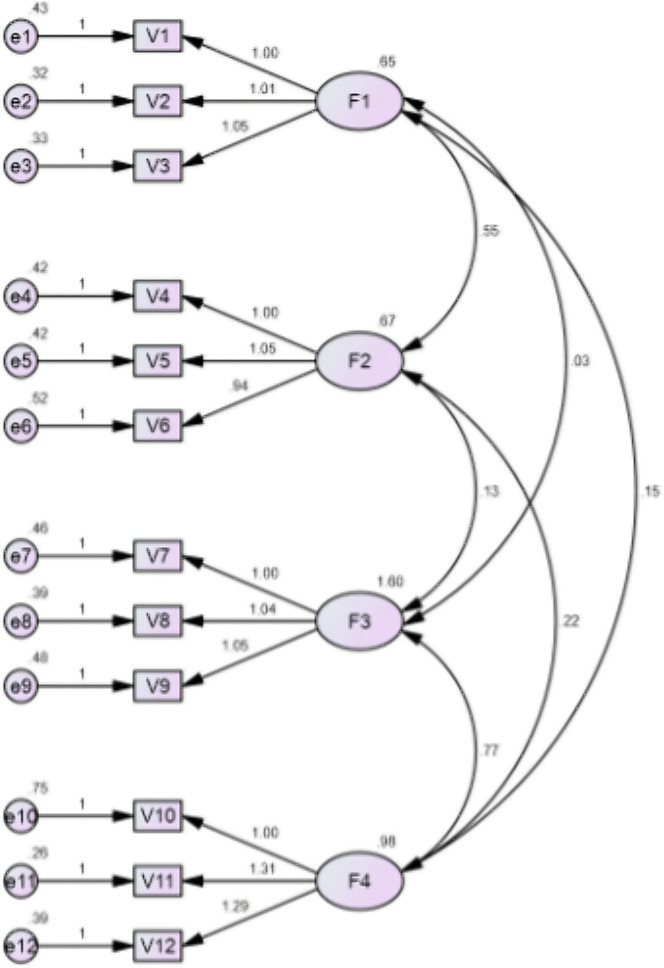
CFA results: Path diagram and standardized estimated parameters for the Vietnamese VAX scale.

The CFA validation of the VAX scale structure also verified the scale’s constructs (see Fig 1).

The interfactor correlations were inconsistent, with the strongest correlation between F1 and F4 (*r* = 0.77) and the weakest between F1 and F2 (*r* = 0.03), indicating a variety of attitudinal dimensions on vaccine attitudes. Measurement invariance across genders showed no differences, implying that the male and female participants understood the VAX scale in the same manner (see S1 Table). Strong associations, such as those between F1–F2 (*r* = 0.61, *p* = 0.01) and VAX_Total-F4(*r* = 0.782, *p* = 0.01), indicated F4’s substantial contribution to unfavorable vaccination beliefs, as evidenced by the Pearson correlations between the subscales and VAX_Total (see S3 Table).

**Fig 2:**
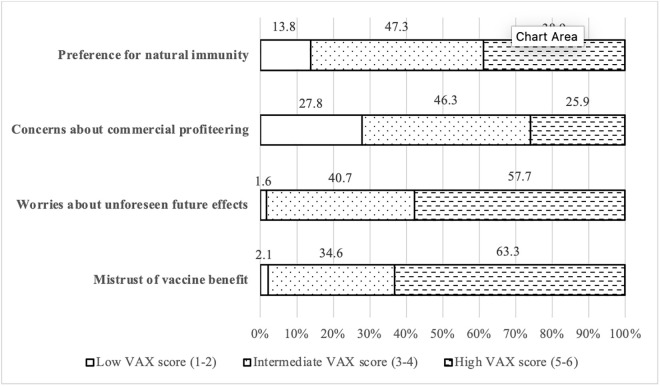
Perceptions of vaccination.

Age was associated with favorable vaccination views, reflecting decreased mistrust (*r* = –0.10, *p* < 0.01), worries about future effects (*r* = –0.09, *p* = 0.05), concern about commercial profiteering (r = –0.07, *p* < 0.05), and preference for natural immunity (F4: r = –0.08, *p < 0.05*). Regarding employment, the ‘Job’ variable (coded from 1 = manual labor to 4 = unemployed/retired) showed a negative correlation with mistrust (*r* = -0.11, *p* < 0.01). This indicates that individuals with stable employment (lower numerical codes) exhibited significantly higher levels of mistrust and vaccine hesitancy compared to those who were unemployed or retired (higher numerical codes). Getting vaccinated was negatively associated with VAX_Total (*r* = –0.15, *p* = 0.01) and particular worries (e.g., profiteering: *r* = –0.14, *p* = 0.01), reflecting favorable attitudes among those receiving the vaccine. The intention to receive vaccination was substantially linked to income (*r* = 0.12, *p* < 0.01) and VAX_Total (*r* = 0.14, *p* < 0.01), albeit with respondents exhibiting mild suspicion and fears (e.g., F1: *r* = 0.18, *p* = 0.01). Knowledge of HBV vaccination was positively correlated with F1 (*r* = 0.23, *p* = 0.01), F2 (*r* = 0.19, *p* = 0.01), F4 (*r* = 0.11, *p* = 0.01), and VAX_Total (*r* = 0.19, *p* = 0.01), denoting that strong knowledge is related to increased fears and cautious attitudes toward vaccination. These results show the complex interaction between demographic attributes, knowledge of vaccines, and vaccination attitudes in the Vietnamese context (see Fig 2).

Among the respondents highly suspicious of vaccine benefits (high group), 63.3% expressed concerns about vaccine efficacy compared with those exhibiting low suspicion (low group, 2.1%). Concerns regarding unexpected consequences were more frequent in the high group (57.7%) than in the low group (1.6%). Commercial profiteering concerns were fairly distributed across the sample, with 46.3% of participants in the intermediate group, 27.8%, and 25.9% exhibiting moderately strong, low, and strong concerns, respectively. Ultimately, the moderate group (47.3%) favored natural immunity the most, followed by the high (38.9%) and low (13.8%) groups. Of the participants, 65.6% were ready to receive vaccination, 19.3% remained dubious, and 15.1% were reluctant.

### Comparison of intention on the basis of VAX subscale mean scores

A one-way ANOVA was conducted to compare the mean scores on the VAX subscales of the refusal, hesitant, and willing groups for HBV vaccination. With modest effect sizes, the willing group scored far higher on F1 (mistrust of vaccine advantages) and F2 (worries about future consequences) than the hesitant or refusal groups (*p* < 0.001). With a *p* < 0.001, the refusal group scored higher on F4 (preference for natural immunity). The groups’ F3 (commercial profiteering concerns) scores had no significant differences (see S2 Table).

### Predictors of HBV vaccine hesitancy and refusal

**Fig 3:**
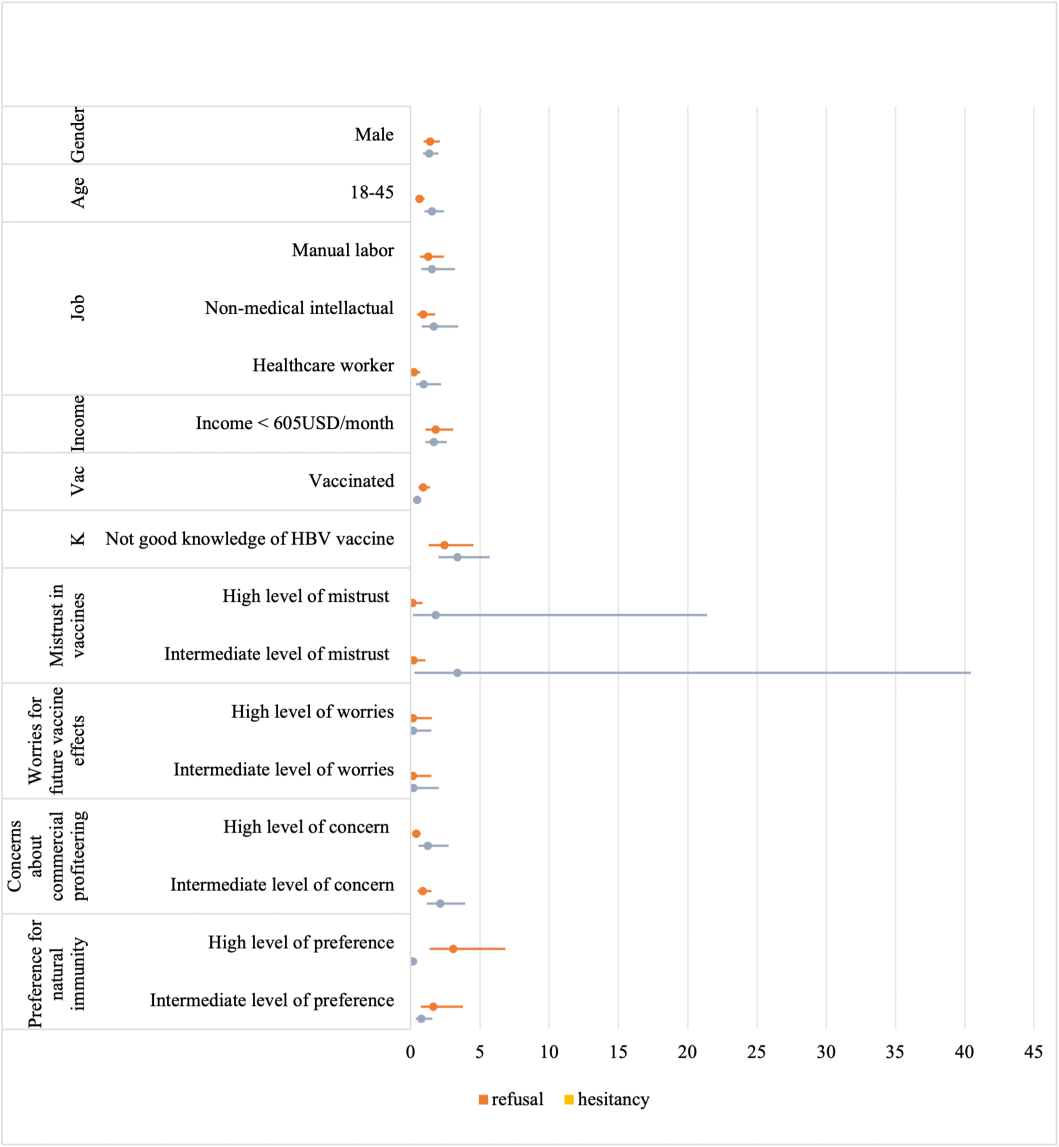
Predictors of HBV vaccine hesitancy and refusal using multinomial logistic regression analysis.

The results indicate that individuals with a high level of mistrust were 85.3% less likely to refuse the vaccine but 78.8% more likely to exhibit hesitancy compared to those with low level of mistrust. Similarly, those with a high level of worries were 82.2% less likely to refuse the vaccine and 85.0% less likely to be hesitant than individuals with low levels of worries. Among participants with a high level of concern about commercial profiteering, vaccine refusal decreased by 59.3%, while hesitancy increased by 41.6% compared to those with low level of concern. In contrast, individuals with a high preference for natural immunity were more than twice as likely to refuse the vaccine and 84.4% less likely to be hesitant compared to those with a low preference. Overall, mistrust and commercial concerns appear to characterize hesitant individuals, whereas preference for natural immunity is more strongly associated with outright refusal (see Fig 3).

### Predictors of refusal to vaccinate against HBV

We found that the refusal to receive the HBV vaccine was higher among the participants with personal incomes of less than US$605 per month (AOR = 1.82, 95% CI: 1.08–3.07) and those with poor knowledge of the HBV vaccine (AOR = 2.42, 95% CI: 1.30–4.53). Such refusal was lower among the healthcare workers (AOR = 0.23, 95% CI: 0.07–0.72) and participants aged 18–44 years (AOR = 0.64, 95% CI: 0.41–0.99). The results indicate a nuanced relationship between attitudes and behavior: individuals with a high level of mistrust were 85.3% less likely to refuse the vaccine (AOR = 0.15, 95% CI: 0.03–0.86) but 78.8% more likely to exhibit hesitancy (AOR = 1.78, 95% CI: 1.05–3.02) compared to those with low levels of mistrust. Similarly, while strong concerns about commercial profiteering reduced the odds of outright refusal (AOR = 0.41, 95% CI: 0.23–0.73), they significantly increased the likelihood of hesitancy by 41.6%. In contrast, a strong preference for natural immunity remained the only attitudinal factor that consistently predicted outright vaccine refusal (AOR = 3.06, 95% CI: 1.37–6.84).

### Predictors of hesitancy to vaccinate against HBV

We identified the predictors of hesitancy and acceptance of the HBV vaccine. As mentioned earlier, hesitancy was observed in the participants with personal incomes of less than US$605 dollars (AOR = 1.66, 95% CI: 1.06–2.60) and those with poor knowledge of the HBV vaccine (AOR = 3.39, 95% CI: 2.01–5.71). Such reluctance was greater among the respondents displaying moderate concerns about commercial profiteering (AOR = 2.14, 95% CI: 1.16–3.95). In contrast, hesitancy was lower among the participants highly preferring natural immunity (AOR = 0.16, 95% CI: 0.07–0.37) (**Fig 3**).

## Discussion

Our findings highlight a nuanced relationship between knowledge, attitudes, and vaccination behaviors. Poor knowledge was identified as a strong predictor of outright vaccine refusal (AOR = 2.42), likely reflecting a lack of perceived need or limited awareness of HBV risks and vaccine benefits. In contrast, higher factual knowledge was positively correlated with higher VAX scores, particularly in the domains of mistrust and worries about future effects (r = 0.19, p < 0.01). Together, these findings suggest a “knowledge paradox,” whereby knowledge may play different roles across the hesitancy–refusal spectrum. Importantly, this finding should not be interpreted as suggesting that limited knowledge is beneficial. Rather, basic knowledge remains necessary to reduce outright refusal by increasing awareness of HBV susceptibility and vaccine benefits. However, higher knowledge may also coexist with “informed skepticism” among urban Vietnamese adults, as greater familiarity with vaccine-related complexities, such as vaccine-induced immunity, antibody titers, post-vaccination testing, and potential adverse effects, may heighten concerns about vaccine safety or long-term implications. This may be reflected in higher VAX scores and may manifest as hesitancy rather than complete refusal. This distinction is important for public health messaging: education campaigns should provide accurate information while also using transparent, empathetic, and trust-building communication to address safety concerns, misconceptions, and confidence in health authorities.

The study demonstrates that the mean VAX scores were 3.0 or higher, suggesting moderate to high skepticism toward vaccination. However, vaccine acceptance remained reasonably high, at 65.6%. This discrepancy implies that vaccination acceptance is not directly predicted by attitudes as assessed by the VAX scale. This could be explained by a number of factors, including perceived dangers of not getting vaccinated, the immediacy of the health threat, cultural and social influences, and the accessibility and affordability of vaccines. For instance, many people decided for vaccination during the COVID-19 pandemic in spite of concerns over its efficacy or safety. It is crucial to remember that the VAX scale evaluates skepticism or trust in vaccines rather than acceptance of them. Consequently, vaccination adoption is impacted by a complex interaction of factors beyond attitude alone, even when VAX scores indicate widespread hesitation.

Our findings support the factorial structure of the VAX scale: mistrust of vaccine benefits (F1), worries about unforeseen future effects (F2), concerns about commercial profiteering (F3), and preference for natural immunity (F4). This four-factor solution aligns with the original VAX structure proposed by Martin and Petrie [17] and is consistent with findings from other cross-cultural validations, such as those conducted in Romania [19], Italy [27], and Turkey [21]. The results also support the reliability and validity of the VAX scale in the Vietnamese context, a culturally distinct setting, suggesting that the scale maintains its psychometric robustness across diverse cultural backgrounds.

Vaccine hesitancy remains one of the major barriers to the elimination of hepatitis B. Numerous studies have assessed the acceptance of the HBV vaccine and have discovered a reluctance to receive vaccination, even in countries with high immunization rates. Common concerns include potential side effects (e.g., multiple sclerosis) [32–34], a perceived low risk of infection in newborns, and a greater fear of vaccine-related harm, such as infant death, than of the disease itself [35–37]. Other contributing factors are the lack of strong recommendations from healthcare providers [38–40], limited media communication [41,42], low education levels, insufficient knowledge about HBV infection risks [43], and a general perception that the vaccine is unnecessary [44], among other issues.

In an Italian sample, high levels of vaccine hesitancy were observed among healthcare workers with low educational attainment, with such hesitancy frequently associated with fears of developing multiple sclerosis, as well as beliefs linking aluminum-based adjuvants in vaccines to Alzheimer’s disease [45]. Racial or ethnic disparities in vaccine acceptance have also been reported. Vulnerable populations, such as pregnant women [46], hemodialysis patients [47], people living with HIV [48], and men who have sex with men, are particularly affected by these attitudes [49].

The configuration, metric, and scalar levels of measurement invariance were all validated in the examination of the factorial structure during the gender-based multigroup analyses. The consistent underlying structure, item loadings, and intercepts across gender groups of the VAX scale validated its effectiveness in evaluating vaccine attitudes equivalently for men and women. Consistent with results of Bruno et al., our findings confirmed the suitability of the scale for gender-based comparisons [27]. Such uniformity and cross-group comparability offer strong support for further studies on gender variations in self-reported vaccine reluctance with conceptual consistency.

Our study found a 65.6% acceptance rate of hepatitis B vaccination, which is lower than that found in the Indonesian context (84.5%), where many participants were healthcare professionals with better awareness of health and access to insurance coverage of vaccination expenses [50]. On the other hand, our rate exceeds that derived in a Chinese study, in which only 20.67% of unvaccinated individuals regarded the hepatitis B vaccine necessary, mostly due to safety and efficacy matters [51]. To contextualize our findings within Southeast Asia, the 65.6% vaccine acceptance rate among urban Vietnamese adults falls between regional variations. Thailand reports higher HBV vaccine willingness, often above 80%, which is attributed to the long-standing success of their Universal Coverage Scheme and high levels of trust in the national immunization system [52]. In contrast, the Philippines faces greater vaccine hesitancy, with acceptance fluctuating due to high-profile controversies and logistical challenges in remote areas [53]. These cross-country variations underscore the influence of sociocultural, informational, and systemic factors that shape vaccine acceptance. These differences in healthcare financing are relevant to our findings, as income was a significant determinant of vaccine refusal and hesitancy in Vietnam, where adult HBV vaccination often requires out-of-pocket payment. Understanding these differences is critical for developing tailored public health interventions. Our findings contribute to the literature by highlighting how contextual factors impact vaccine tolerance across diverse populations.

With a projected population of 8.9 million by mid-2023, Ho Chi Minh City, Vietnam’s most populous and economically vibrant city, offers a unique socioeconomic environment that might help explain its high hepatitis B vaccination rates [54]. As a major financial, commercial, educational, and technological hub, the city has undergone rapid urbanization and significant healthcare improvements through substantial investments in infrastructure and services. These developments have enhanced public access to healthcare information and services, increasing awareness of the importance of vaccination. Additionally, coordinated efforts among local authorities, NGOs, and healthcare providers have been pivotal in building public trust and encouraging participation in vaccination campaigns.

Despite these favorable conditions and ongoing public health initiatives, our findings reveal that the hepatitis B vaccination acceptance rate is lower than might be expected for a city with such advancements. This discrepancy suggests the presence of persistent barriers, such as vaccine hesitancy, misinformation, or unequal access among certain population groups. These factors highlight the complexity of vaccine acceptance and indicate that socioeconomic progress and campaign efforts alone may not be sufficient to achieve optimal vaccination coverage. Therefore, a deeper understanding of these contextual challenges is necessary to tailor more effective interventions and policies to increase vaccine uptake in Ho Chi Minh City.

### Relationship between demographic characteristics and vaccine hesitancy and refusal

Contrary to earlier studies showing gender differences in vaccine uptake, gender was not a significant predictor of hepatitis B vaccine refusal in the present research. For example, a study among Laotian Americans found that women are far more likely to receive vaccination (adjusted OR = 4.0, 95% CI: 1.2–19), whereas men tend to believe in the preventability and treatability of HBV [55]. Similarly, studies on healthcare workers in five developing nations found that female workers are predisposed to complete the hepatitis B vaccine series, albeit the findings varied by setting [56]. Gender variations were also discovered in relation to other vaccines, with women found to be more sensitive to side effects or more strongly affected by issues such as pregnancy [57]. These contradictions imply that sociocultural standards, healthcare availability, and vocational positions influence the effects of gender on vaccination acceptance. The absence of a gender effect in our study may reflect specific sociocultural factors in Vietnam. Traditional gender roles, coupled with relatively equal healthcare access and vocational opportunities in urban settings, might reduce gender disparities in vaccination acceptance. This contrasts with findings from other contexts, highlighting the importance of localized sociocultural and structural influences.

In this study, income level was a major determinant of the reluctance or refusal to get vaccinated. Low earners were around 1.8 times more likely than high-income individuals to refuse or hesitate with respect to vaccination. This corresponds with prior research on social inequalities in immunization rates, such as studies in rural China highlighting poverty as a primary factor in hepatitis B vaccination disparities [58]. In the Vietnamese context, this financial barrier is particularly acute for adults. While the National Expanded Program on Immunization provides free HBV vaccines for children, the National Action Plan for 2021–2025 does not subsidize vaccination for the broader adult population. Consequently, adults must pay out-of-pocket for voluntary vaccination services. For low-income individuals, these costs represent a significant structural barrier, intensifying hesitancy and making economic status a primary predictor of vaccine uptake.

In interpreting the findings on vaccine hesitancy and refusal, it is important to clarify how the VAX variable captures attitudes toward hepatitis B vaccination. The VAX measure reflects participants’ general views about vaccination, regardless of whether they have previously been vaccinated. Therefore, even individuals who have already received one or more doses may still report “accept,” “hesitant,” or “refusal” depending on their current attitude. Based on this framework, the refusal group likely includes those who do not intend to vaccinate (108 participants) as well as some previously vaccinated individuals who could refuse additional doses or oppose recommending vaccination. The hesitant group may include individuals who have initiated vaccination but remain uncertain about completing the series. Meanwhile, the accept group consists of those who intend to vaccinate (275 participants) and those who are already vaccinated. Distinguishing between actual vaccination status and future vaccination intention helps resolve the seeming inconsistency in the results and provides a clearer understanding of how attitudes toward vaccination vary across demographic groups.

Individuals with inadequate knowledge regarding HBV vaccination exhibit vaccine refusal or hesitancy that is two to three times greater than that among those with a sufficient understanding of the issue. Research conducted in Indonesia uncovered that a core obstacle to immunization is ignorance of its advantages [59]. Similar trends were noted in Vietnam and Uganda, where little knowledge of HBV and its transmission diminishes the acceptability of vaccination [60,61]. Efforts should thus prioritize low-awareness communities, with easily available, culturally acceptable resources used as part of campaigns. Particularly in rural or underprivileged regions, tools such as illustrated fliers, pamphlets, and short educational movies with clear graphics should be extensively available in local health clinics, schools, marketplaces, and mobile health units [62]. Involving local healthcare professionals, leaders, and influencers can also help build confidence and increase community participation. These strategies can increase health literacy, thereby reducing hesitation and encouraging the equitable distribution of HBV vaccines.

Our findings provide a critical distinction between vaccine hesitancy and refusal: mistrust of vaccine benefits (F1) and concerns about commercial profiteering (F3) manifested as cautious indecision rather than outright rejection. Specifically, individuals with high mistrust were significantly less likely to refuse the vaccine (AOR = 0.15) but more likely to be hesitant (AOR = 1.78) compare to those with low mistrust. This paradox suggests that despite underlying skepticism, the high prevalence of HBV in Vietnam leads individuals to pragmatically recognize vaccination as an essential preventive measure, causing them to vacillate or postpone (hesitancy) rather than definitively decline (refusal). In contrast, a strong preference for natural immunity (F4) remains the primary and consistent driver of absolute vaccine refusal (AOR = 3.06), suggesting that these individuals hold an ideological resistance that is much firmer than the doubts held by the hesitant group. This aligns with findings in South Africa, where vaccine distrust was identified as the strongest predictor of hesitancy, leading individuals to postpone vaccination rather than avoid it completely [63]. Similarly, a Turkish study among healthcare professionals identified mistrust as a key driver of vaccine reservations [64]. In Vietnam, the high prevalence of HBV may lead individuals to pragmatically recognize vaccination as an essential preventive measure despite their underlying skepticism, encouraging them to eventually proceed with vaccination rather than absolute refusal.

Regarding worries about unforeseen future effects, our results indicated that a high level of concern reduced the likelihood of both refusal (by 82.2%) and hesitancy (by 85.0%). This indicates that even individuals with greater worries appear less likely to avoid the vaccine when the perceived necessity of protection against HBV is high. Consistent with research conducted in France, our findings demonstrated that concerns about unanticipated future impacts did not exert a dominant influence on vaccine rejection or hesitation in the context of HBV [33]. This suggests that while future risks are a consideration, they do not necessarily translate into a barrier for vaccine uptake when the immediate threat of disease is significant.

Participants who perceived vaccine promotion as being primarily driven by commercial profiteering were 59.3% less likely to refuse the vaccine but 41.6% more likely to be hesitant. This pattern mirrors observations in other contexts where moderate anxiety about commercial motives increases hesitation without leading to a definitive decision to decline [65]. Such hesitation often stems from a lack of institutional trust rather than opposition to the vaccine itself. Therefore, improving transparency in distribution and pricing, while emphasizing public health benefits over commercial interests, is a critical strategy for reducing these doubts and encouraging acceptance.

In contrast to other domains, a strong preference for natural immunity was a definitive predictor of vaccine rejection. Individuals in this group were more than twice as likely to refuse vaccination and 84.4% less likely to be hesitant, suggesting they tend to make firm decisions against vaccination rather than remain uncertain. This result is consistent with findings in France and other settings, where a belief in the superiority of natural infection leads to a three-fold increase in vaccine rejection [33,66–68]. In Vietnam, where deep rooted beliefs about natural protection may pose a challenge, public health communication must go beyond providing information to emphasize how vaccination safely strengthens, rather than replaces, natural immunity.

Vaccine refusal and hesitation ultimately result from a confluence of psychological and sociodemographic factors. The results can inform future studies and enable public health experts to create sensible plans. Important activities include addressing personal concerns, offering accurate information on vaccine safety and efficacy, countering misinformation, and involving medical professionals in education campaigns. Thus, public health interventions should adopt an integrated approach that addresses attitudinal concerns, institutional trust, and financial barriers to adult HBV vaccination, particularly among low-income adults.

### Strengths and limitations of the study

This study presents several strengths. Notably, the VAX scale’s reliability and validity in the Vietnamese context are guaranteed by the fact that it is the first to do a psychometric validation of the scale among Vietnamese adults, confirming its original four-factor structure and proving measurement invariance across genders. The study also finds actionable correlations of vaccine reluctance and refusal, such as income, hepatitis B vaccination knowledge, natural immunity beliefs, and commercial interest concerns. Last but not least, these results establish precise programmatic goals to raise adult hepatitis B vaccination rates, giving legislators important direction for creating efficient vaccination laws, improving program execution, and customizing public health initiatives. A primary limitation of this study is the potential selection bias stemming from the convenience sampling conducted exclusively in Ho Chi Minh City (HCMC), a highly developed metropolitan area with superior healthcare access and information. Recruitment from public spaces such as cafés, parks, and supermarkets may have further biased the sample toward individuals with greater leisure time, higher mobility, or more active social engagement, while underrepresenting those who are housebound, have demanding work schedules, or limited mobility. Consequently, the findings may have limited generalizability to rural or less developed regions of Vietnam, where healthcare infrastructure and socio-cultural factors differ substantially. Additionally, reliance on self-reported data may introduce social desirability and reporting biases. Due to the cross-sectional design, causal inferences cannot be drawn from the observed associations. Furthermore, residual confounding from unmeasured variables cannot be entirely excluded despite adjustment for multiple covariates. Future research should consider longitudinal designs, diversify data collection methods, and randomize item presentation order to minimize potential biases and improve validity.

## Conclusion

Despite favorable conditions and ongoing public health efforts in Ho Chi Minh City, the acceptance of the hepatitis B vaccine remains lower than expected, indicating the presence of persistent barriers such as vaccine hesitancy, misinformation, and unequal access. Our research emphasizes that vaccine decision-making is influenced by the strength and lucidity of personal attitudes toward vaccination, in addition to existing concerns. The VAX scale appears to be a reliable instrument for assessing vaccine attitudes among urban Vietnamese adults, particularly in metropolitan settings such as Ho Chi Minh City. Nevertheless, because this study was conducted in an urban population, the generalizability of these findings to rural communities and other geographical regions of Vietnam remains limited. Broader applicability of the scale requires additional research in more diverse geographic settings. By pinpointing specific factors contributing to hesitancy, this scale can help tailor public health interventions more effectively, ultimately improving vaccination rates.

## Supporting information

S1 Table Test of the invariance of VAX scale across gender. (DOCX)

S2 Table One-way Anova results of the difference among the total score of the VAX and intention to get HBV vaccine. (DOCX)

S3 Table Pearson’s correlation, means, standard deviation (SD) for VAX subscales, and total score. (DOCX)

S1 STROBE Checklist Checklist of items that should be included in reports of cross-sectional studies. (DOCX)

## Abbreviation

HBV: Hepatitis B Virus
CDC: Centers for Disease Control and Prevention
CFA: Confirmatory factor analysis
DALYs: disability-adjusted life years
HCV: Hepatitis C Virus
HCMC: Ho Chi Minh City
NGOs: non-governmental organizations
OR: Odds Ratio
KAP: Knowledge Attitude and Practice
SES: Socioeconomic status
VAX: Vaccination Attitudes Examination
WHO: World Health Organization
