# Supplementary material for: Willingness to accept the HBV vaccine and related factors: Administering the vaccination attitudes examination scale to Urban Vietnamese adults
Source: PLOS Glob Public Health. 2026 Jul 22;6(7):e0006886. doi: 10.1371/journal.pgph.0006886 (PMC13390869; doi:10.1371/journal.pgph.0006886)
Supplement: S1 Table — (DOCX) [file pgph.0006886.s001.docx]

**S1 Table. Test of invariance in the VAX scale across genders.**

| **Model** | **χ^2^** | **df** | **Δχ^2^** | **Δdf** | **CFI** | **TLI** | **RMSEA** | **SRMR** | **ΔCFI** |
| --- | --- | --- | --- | --- | --- | --- | --- | --- | --- |
| Male | 125.14 | 48.00 |  |  | 0.98 | 0.97 | 0.06 | 0.04 |  |
| Female | 159.20 | 48.00 |  |  | 0.97 | 0.96 | 0.07 | 0.05 |  |
| Configural | 284.34 | 96.00 | NA | NA | 0.97 | 0.96 | 0.07 | 0.04 | NA |
| Metric | 296.95 | 104.00 | 12.61 | 8.00 | 0.97 | 0.97 | 0.06 | 0.04 | 0.00 |
| Scalar | 306.19 | 112.00 | 9.23 | 8.00 | 0.97 | 0.97 | 0.06 | 0.04 | 0.00 |
