# Supplementary material for: Willingness to accept the HBV vaccine and related factors: Administering the vaccination attitudes examination scale to Urban Vietnamese adults
Source: PLOS Glob Public Health. 2026 Jul 22;6(7):e0006886. doi: 10.1371/journal.pgph.0006886 (PMC13390869; doi:10.1371/journal.pgph.0006886)
Supplement: S2 Table — (DOCX) [file pgph.0006886.s002.docx]

**S2Table. One-way ANOVA results of the differences among VAX total scores on the intention to receive the HBV vaccine.**

| **F** | **Refusal**  **(N=140)** | | **Hesitant**  **(N=179)** | | **Willing (N=607)** | | **df** | **p** |
| --- | --- | --- | --- | --- | --- | --- | --- | --- |
|  | **Mean** | **SD** | **Mean** | **SD** | **Mean** | **SD** |  |  |
| F1 | 12.78 | 3.34 | 12.23 | 2.21 | 13.76 | 2.50 | 923 | <0.01 |
| F2 | 12.86 | 3.37 | 11.83 | 2.28 | 13.33 | 2.53 | 923 | <0.01 |
| F3 | 8.98 | 4.28 | 9.70 | 3.05 | 9.83 | 4.26 | 923 | 0.08 |
| F4 | 12.11 | 3.67 | 10.04 | 2.85 | 11.25 | 3.86 | 923 | <0.001 |
