# Supplementary material for: Willingness to accept the HBV vaccine and related factors: Administering the vaccination attitudes examination scale to Urban Vietnamese adults
Source: PLOS Glob Public Health. 2026 Jul 22;6(7):e0006886. doi: 10.1371/journal.pgph.0006886 (PMC13390869; doi:10.1371/journal.pgph.0006886)
Supplement: S3 Table — (DOCX) [file pgph.0006886.s003.docx]

**S3 Table. Pearson’s correlations, means, standard deviations of the VAX subscales and total scores.**

|  | **Mean** | **SD** | **Gender** | **Age** | **Job** | **Income** | **Vac** | **ItoV** | **K** | **F1** | **F2** | **F3** | **F4** | **VAX_Total** |
| --- | --- | --- | --- | --- | --- | --- | --- | --- | --- | --- | --- | --- | --- | --- |
| Gender | 1.50 | 0.50 | 1.00 |  |  |  |  |  |  |  |  |  |  |  |
| Age | 1.35 | 0.48 | 0.01 | 1.00 |  |  |  |  |  |  |  |  |  |  |
| Job | 1.99 | 0.96 | 0.15^**^ | 0.15^**^ | 1.00 |  |  |  |  |  |  |  |  |  |
| Income | 1.27 | 0.44 | -0.03 | 0.05 | 0.06 | 1.00 |  |  |  |  |  |  |  |  |
| Vac | 1.41 | 0.49 | -0.12^**^ | -0.10^**^ | -0.29^**^ | -0.20^**^ | 1.00 |  |  |  |  |  |  |  |
| ItoV | 2.50 | 0.74 | 0.07^*^ | -0.05 | 0.08 | 0.12^**^ | -0.14^**^ | 1.00 |  |  |  |  |  |  |
| K | 0.87 | 0.34 | -0.06^*^ | 0.05 | 0.01 | 0.05 | -0.12^**^ | 0.18^**^ | 1.00 |  |  |  |  |  |
| F1 | 2.61 | 0.53 | 0.02 | -0.10^**^ | -0.11^**^ | -0.03 | -0.04 | 0.18^**^ | 0.23^**^ | 1.00 |  |  |  |  |
| F2 | 2.56 | 0.53 | 0.04 | -0.09^**^ | -0.13^**^ | -0.06 | -0.01 | 0.15^**^ | 0.19^**^ | 0.61^**^ | 1.00 |  |  |  |
| F3 | 1.98 | 0.73 | -0.08^*^ | -0.07^*^ | -0.05 | -0.08^*^ | 0.17^**^ | 0.08^*^ | 0.03 | 0.02 | 0.11^**^ | 1.00 |  |  |
| F4 | 1.50 | 0.50 | -0.16^**^ | -0.08^*^ | -0.13^**^ | -0.16^**^ | 0.23^**^ | 0.01 | 0.11^**^ | 0.22^**^ | 0.28^**^ | 0.52^**^ | 1.00 |  |
| **VAX_Total** | 1.35 | 0.48 | -0.08^*^ | -0.12^**^ | -0.15^**^ | -0.13^**^ | 0.15^**^ | 0.14^**^ | 0.19^**^ | 0.60^**^ | 0.66^**^ | 0.68^**^ | 0.782^**^ | 1.00 |
| **Notes.** Job (from 1 = Manual labor, 2= Non-medical intellectual labor, 3=Healthcare worker, 4=Homemaker /Retired/ Unemployed); Income =  Personal Income (US Dollar/month); Vac  = Vaccination (1 =  Vaccinated, 2 = Not yet); ItoV = Intention to vaccination; K= Knowledge about vaccine; F1 = mistrust of vaccine benefit; F2 = worries over unforeseen future effects; F3 = concerns about commercial profiteering; F4 = preference for natural immunity.  **^*^*p* < 0.05; ***p* < 0.01** | | | | | | | | | | | | | | |
