# Supplementary material for: Willingness to accept the HBV vaccine and related factors: Administering the vaccination attitudes examination scale to Urban Vietnamese adults
Source: PLOS Glob Public Health. 2026 Jul 22;6(7):e0006886. doi: 10.1371/journal.pgph.0006886 (PMC13390869; doi:10.1371/journal.pgph.0006886)
Supplement: Attachment — Submitted filename: Willingness to accept the HBV vaccine and related factors.pdf [file pgph.0006886.s008.pdf]

Willingness to accept the HBV vaccine and related factors: Administering the vaccination attitudes examination scale to Vietnamese adults

Recommendation: Major Revisions

Summary

This manuscript presents a cross-sectional study involving 926 adults in Vietnam, investigating the acceptance of the Hepatitis B virus (HBV) vaccine using the Vaccination Attitudes Examination (VAX) scale. The study aims to identify correlates of vaccine hesitancy and refusal. The authors report that 65.6% of participants accept the vaccine, while 19.3% are hesitant and 15.1% refuse it. The study validates the VAX scale in the Vietnamese context and explores the relationship between sociodemographic factors, knowledge, and vaccination attitudes.

While the topic is highly relevant to public health in Vietnam and the use of the VAX scale adds methodological value, there are significant inconsistencies in the reporting of results, particularly regarding the relationship between mistrust, concerns and vaccine refusal. Furthermore, there are concerns regarding the sampling frame's generalizability and contradictions between the abstract and the main text.

Major Concerns

1. Contradictions Regarding Predictors of Refusal and Hesitancy (Critical) There are severe discrepancies between the Abstract, the Results section, and the Discussion regarding the direction of the associations between VAX subscales (mistrust, commercial concerns) and vaccine refusal.

- Abstract vs. Results:
  - The Abstract states: *"Vaccine refusal was linked to... mistrust..."*
  - The Results section states: *"Refusal was lower among the respondents who were substantially concerned about commercial profiteering... but lower among those who mistrusted vaccine benefits (AOR = 0.15)."*
  - An AOR of 0.15 implies that those with high mistrust are *85% less likely* to refuse the vaccine compared to the reference group. This contradicts the abstract and the fundamental premise of the study (that negative attitudes lead to refusal).
- Interpretation of Odds Ratios: The authors also report that individuals with high commercial concerns had decreased refusal (AOR 0.41). It is counter-intuitive that higher mistrust and higher commercial concerns would lead to *lower* odds of refusal. The authors must double-check the coding of the outcome variable (Reference category: Acceptance) and the predictor variables.
  - Did the authors reverse the coding for the "High" vs. "Low" VAX groups in the regression analysis?

    - Alternatively, does the "Refusal" group in the regression actually represent "Acceptance"?
- Action: The authors need to perform a rigorous audit of their multinomial logistic regression output, recode variables if necessary to ensure logical consistency (e.g., High Mistrust should logically increase the odds of refusal/hesitancy), and correct the Abstract and Results accordingly.

2. Generalizability and Sampling Method

- The title claims the study applies to "Vietnamese adults," but the sampling method was strictly convenience-based in "public spaces, such as parks, cafés, and supermarkets" in Ho Chi Minh City (HCMC).
- HCMC is the most developed economic hub in Vietnam. Attitudes toward healthcare and vaccines there are not representative of rural areas, the Mekong Delta, or Northern highlands.
- Action: The title should be modified to reflect the specific population studied (e.g., "Adults in Ho Chi Minh City" or "Urban Vietnamese Adults"). The limitations section must explicitly discuss how urban convenience sampling biases the results.

3. The "Knowledge Paradox"

- The study reports a positive correlation between HBV knowledge and negative attitudes (mistrust, worries about future effects, preference for natural immunity). The authors suggest this is because "individuals who have a deeper acquaintance... develop a stronger awareness of rare adverse effects."
- While "informed skepticism" is a valid phenomenon, the strength of the positive correlation (for mistrust) is notable. The "Knowledge" section of the questionnaire (Items K1-K10) appears to measure factual knowledge.
- Action: The authors should verify the content of the knowledge questions. Do any questions inadvertently reinforce negative myths (e.g., asking about side effects in a leading way)? If the questions are purely factual, the discussion needs to expand significantly on why health education in this population is correlating with *increased* hesitancy, rather than the standard assumption that knowledge increases compliance.

4. Internal Consistency and Redundancy

- The Cronbach's alpha for the VAX scale is reported as 0.963, which is exceptionally high. While this indicates reliability, values above 0.95 often suggest item redundancy, where multiple items are measuring the exact same concept without adding new information.

- Action: The authors should review the inter-item correlations. If redundancy is found, a shorter version of the scale might be more efficient for future public health use.

Specific Comments

Abstract

- *Inconsistency:* As noted above, please align the statement "Vaccine refusal was linked to... mistrust" with the regression results presented later in the text.

Introduction

- *Tone:* The introduction is well-structured but could benefit from briefly mentioning specific cultural factors in Vietnam that might influence natural immunity preferences (e.g., Traditional Medicine usage).

Materials and Methods

- *Author Order:* There is a discrepancy in the author order between the manuscript header ("Order of Authors: Tram Thi Huyen Nguyen, Tung Hoang...") and the title page in the document text ("Nghi Ho Hoc, Tung Hoang..."). Please ensure the author list is consistent and meets the journal's criteria for authorship.

Results

- *Table 1:* The income categorization cutoff is US$605. It would be helpful to contextually explain what this threshold represents (e.g., minimum wage, average income) to assist international readers in interpreting "low income."
- *Correlations:* The text states: *"Knowledge... was positively correlated with mistrust... ($r = 0.23$)."* Later, it states: *"Employed individuals exhibited increased vaccine hesitancy... negative associations across categories (e.g., mistrust $r = -0.11$)."* Please clarify which variables are being correlated in the second instance to avoid confusion.
- *Regression Interpretation:* The results state: *"Individuals with a high level of mistrust were 85.3% less likely to refuse the vaccine but 78.8% more likely to exhibit hesitancy."* This suggests that mistrust moves people from "Refusal" to "Hesitancy"? This is a nuanced finding. If accurate, it implies that total rejection (refusal) is driven by *other* factors (like natural immunity preference), while mistrust keeps people in the "hesitant" camp. This is a fascinating insight but requires clearer explanation.

Discussion

- *Discrepancy:* The discussion states: *"refusal was lower among... those who mistrusted vaccine benefits."* It then attempts to explain why mistrust leads to refusal. This section contradicts the quantitative findings reported in the

Results section. The entire discussion regarding predictors needs to be rewritten to match the (corrected) regression outputs.

- *Comparison:* The comparison with the Indonesian study (84.5% acceptance) and Chinese study (20.67% necessity) is good. Adding a comparison with recent HBV acceptance studies in similar Southeast Asian contexts (e.g., Thailand or Philippines) would strengthen the regional relevance.

Conclusion

- The conclusion is generally appropriate but should be tempered by the sampling limitations. It claims the VAX scale is effective for "Vietnam" generally; rephrase to indicate it is effective for the sampled population (urban adults), pending further validation in rural areas.

Conclusion

This manuscript addresses an important topic with a potentially large sample size. However, the contradictions between the abstract and results regarding the direction of associations (particularly for mistrust and commercial concerns) undermine the validity of the findings. A major revision is required to correct the statistical reporting, resolve the "future date" error, and align the title with the sampling scope. Once these inconsistencies are addressed, the paper will make a solid contribution to the literature on vaccine hesitancy in Vietnam.
