# Supplementary material for: Willingness to accept the HBV vaccine and related factors: Administering the vaccination attitudes examination scale to Urban Vietnamese adults
Source: PLOS Glob Public Health. 2026 Jul 22;6(7):e0006886. doi: 10.1371/journal.pgph.0006886 (PMC13390869; doi:10.1371/journal.pgph.0006886)
Supplement: Attachment — Submitted filename: PGPH-D-25-02314_R1_reviewer_03.18.2026.pdf [file pgph.0006886.s009.pdf]

PLOS Global Public Health

Willingness to accept the HBV vaccine and related factors: Administering the vaccination attitudes examination scale to Vietnamese adults

--Manuscript Draft--

**Manuscript Number:**	PGPH-D-25-02314R1
**Article Type:**	Research Article
**Full Title:**	Willingness to accept the HBV vaccine and related factors: Administering the vaccination attitudes examination scale to Vietnamese adults
**Short Title:**	Acceptance of HBV Vaccine among Vietnamese Adults
**Corresponding Author:**	Tram Thi Huyen Nguyen, Ph.D., B.Pharm<br>Vietnam National University Ho Chi Minh City<br>Hochiminh, Ho Chi Minh [Sai Gon] VIET NAM
**Order of Authors:**	Tram Thi Huyen Nguyen, Ph.D., B.Pharm
	Tung Hoang, PhD.
	Cong Minh Le, PhD.
	Nghi Hoc Ho, B.Pharm
**Keywords:**	VAX; HBV; Attitude; Vietnam; vaccination
**Abstract:**	Vietnam is one of the countries in the East Asian region with a low hepatitis B virus (HBV) vaccination rate, posing a serious threat to the country's healthcare system. To mitigate the negative impacts of low herd immunity, it is essential to identify the population's level of vaccine hesitancy and refusal toward the HBV vaccine as well as the sociodemographic factors correlated with these behaviors. Accordingly, a cross-sectional study involving 926 adults were conducted in Vietnam from January to March 2025. A self-administered questionnaire and the vaccination attitudes examination (VAX) scale were used. Descriptive analysis was performed, and associations between variables were explored using a chi-square test. Multinominal logistical regression was conducted to identify the correlates of vaccine hesitancy and refusal. A $p < 0.05$ was considered indicative of statistical significance. The overall Cronbach's alpha was 0.963 (range = 0.812–0.919), and the McDonald's omega coefficients ranged from 0.820 to 0.920. The uniqueness values (0.115–0.422) indicated that substantial variances were explained by latent factors, with significant standardized loadings of 0.94 to 2.31 ($p < 0.001$). Knowledge about the HBV vaccine was positively correlated with mistrust of vaccine benefits ($r = 0.23$, $p < 0.01$), worries about future effects ($r = 0.19$, $p < 0.01$), preference for natural immunity ($r = 0.11$, $p < 0.01$), and total VAX score ($r = 0.19$, $p < 0.01$). Vaccine refusal was linked to age, occupation, income, knowledge, mistrust, commercial concerns, and preference for natural immunity, while hesitancy was associated with income, vaccination history, commercial concerns, and preference for natural immunity. The VAX scale is a valuable tool for assessing vaccine resistance and refusal in Vietnam as well as public health communication programs. It also effectively identifies key vaccine-related reservations.
**Additional Information:**	
**Question**	**Response**
**Financial Disclosure**<br><br>Enter a financial disclosure statement that describes the sources of funding for the work included in this submission and the role the funder(s) played. This includes grants and any commercial funding of the	This research is funded by Vietnam National University Ho Chi Minh City (VNUHCM) under grant number 36-2025-44-02. The funders had no role in study design, data collection and analysis, decision to publish, or preparation of the manuscript.

Powered by Editorial Manager® and ProduXion Manager® from Aries Systems Corporation

work or authors.<br><br>This statement will be typeset if the manuscript is accepted for publication.<br><br>*Please review the submission guidelines and the instructions link below for detailed requirements and guidance.*	
**Competing Interests**<br><br>On behalf of all authors, disclose any competing interests that could be perceived to bias this work.<br><br>This statement will be typeset if the manuscript is accepted for publication.<br><br>*Please review the instructions link below and PLOS Global Public Health's competing interests policy to determine what information must be disclosed at submission.*	The authors have declared that no competing interests exist.
**Data Availability**<br><br>Before publication, Authors are required to make fully available and without restriction all data underlying their findings. Please see our PLOS Data Policy page for detailed information on this policy.<br><br>A **Data Availability Statement**, detailing where the data can be accessed, is required at first submission. Insert your Data Availability Statement in the box below.<br><br>Please see the data reporting section of our submission guidelines for instructions on what you need to include in your Data Availability Statement.	All relevant data are within the paper and its Supporting Information files.

This statement will be typeset if the manuscript is accepted for publication.<br><br>*PLOS allows rare exemptions to address legal and ethical concerns. If you have legal or ethical restrictions, please use the box below to detail these in full sentences for the Journal team to consider.*	

Powered by Editorial Manager® and ProduXion Manager® from Aries Systems Corporation

Manuscript

Click here to access/download;Manuscript;VAX.HBV.Ver2.fin.docx

Willingness to accept the HBV vaccine and related factors: Administering the vaccination attitudes examination scale to Vietnamese adults

Nghi Ho Hoc[1], Tung Hoang[1], Cong Minh Le[2], Tram Thi Huyen Nguyen [1,*],

[1] Faculty of Pharmacy, University of Health Sciences, Vietnam National University Ho Chi Minh City, Ho Chi Minh city 700000, Vietnam.

[2] Center for Mental Health Research and Support, University of Social Sciences and Humanities, Vietnam National University Ho Chi Minh City, Ho Chi Minh City, 700000, Vietnam

***Corresponding author: Tram Thi Huyen Nguyen (PhD., BSc. Pharm.)**

[1] Faculty of Pharmacy, University of Health Sciences, Vietnam National University Ho Chi Minh City, Ho Chi Minh city 700000, Vietnam.

Add: YA1 Administrative Building, Hai Thuong Lan Ong Street, Dong Hoa Ward, Di An City, Binh Duong Province, Vietnam.

Email: nthtram@uhsvnu.edu.vn

Abstract

Vietnam is one of the countries in East Asia with a low hepatitis B vaccination coverage, posing a serious threat to the country's healthcare system. To mitigate the negative impacts of low herd immunity, it is essential to identify the population's level of vaccine hesitancy and refusal toward the HBV vaccine as well as the sociodemographic factors correlated with these behaviors. We conducted a cross-sectional survey of 926 adults in Vietnam from January to March 2025. When analyzing general vaccine intentions regardless of vaccination status, 65.6% of participants indicated acceptance, 19.3% hesitancy, and 15.1% refusal. A self-administered questionnaire and the vaccination attitudes examination (VAX) scale were used. Descriptive analysis was performed, and associations between variables were explored using a chi-square test. Multinominal logistical regression was conducted to identify the correlates of vaccine hesitancy and refusal. A $p < 0.05$ was considered indicative of statistical significance. The overall Cronbach's alpha was 0.963 (range = 0.812–0.919), and the McDonald's omega coefficients ranged from 0.820 to 0.920. The uniqueness values (0.115–0.422) indicated that substantial variances were explained by latent factors, with significant unstandardized loadings of 0.94 to 1.31 ($p < 0.001$). Knowledge about the HBV vaccine was positively correlated with mistrust of vaccine benefits ($r = 0.23$, $p < 0.01$), worries about future effects ($r = 0.19$, $p < 0.01$), preference for natural immunity ($r = 0.11$, $p < 0.01$), and total VAX score ($r = 0.19$, $p < 0.01$). Vaccine refusal was linked to age, occupation, income, knowledge, mistrust, commercial concerns, and preference for natural immunity, while hesitancy was associated with income, vaccination history, commercial concerns, and preference for natural immunity. The

VAX scale serves as a powerful and reliable instrument for assessing vaccine resistance and refusal in Vietnam. It not only helps identify key vaccine-related concerns but also supports continuous monitoring of public attitudes, which is essential for designing effective vaccination programs and enhancing public trust in vaccines.

Introduction

Hepatitis remains one of the most serious public health threats of the 21st century due to its severe long-term complications, such as liver cirrhosis, liver failure, liver cancer, and hepatocellular carcinoma, a problem that has prompted extensive global efforts to control and eliminate the disease [1]. Globally, approximately 254 million individuals were afflicted with hepatitis B virus (HBV), which was responsible for an estimated 1.1 million deaths, accounting for 83% of all viral hepatitis-related fatalities.The burden of chronic hepatitis B and C is highest among individuals aged 30 to 54 years, who represent half of all cases, while children under 18 years account for 12%. Men constitute 58% of the total infected population [1]. Moreover, even if the incidence of HBV infections has decreased globally, geographic disparities still exist. For example, the WHO stated that African and Southeast Asian regions are particularly affected, with these areas suffering from a 3% prevalence of the disease as well as bearing the burden of 60.5 million afflicted people and 180,000 HBV-related fatalities in 2019 [2].

For developing countries, conversely, achieving these goals remains particularly challenging. Vietnam, a middle-income nation in Southeast Asia with a population of 97 million, has an HBV prevalence of 7% to 8% [3], ranking 9th globally in terms of disease burden. In the country, as well, HBV infection is the leading cause of liver

cancer [4], with chronic hepatitis B accounting for up to 62.3% of cases at Cho Ray Hospital in Ho Chi Minh City [5].

Vaccination remains a key intervention in eliminating the HBV infection, consistent with the WHO's 2030 global elimination target. The hepatitis B vaccine is a safe and effective method of preventing HBV infection, with a birth dose and subsequent boosters providing nearly 100% protection [6]. Advancements in vaccine development also promise to curb HBV transmission by reducing mother-to-child transmission and enhancing vaccine immunogenicity in at-risk adult groups [7]. A one-year interruption in birth-dose and childhood HBV vaccination could cause 5.3 million additional chronic infections from 2020 to 2030, leading to 1 million more HBV-related deaths [6]. Vietnam has used HBV vaccination as part of its national immunization program since 2003, achieving the WHO target of 90% coverage for three doses and 50% coverage for birth doses by 2020 [8]. Nonetheless, vaccination rates among adults, those born before the implementation of the National Expanded Program on Immunization for children, remain low. This is largely due to Vietnam's National Action Plan for the Prevention and Control of Hepatitis B (2021–2025), which prioritizes children and high-risk groups rather than the broader adult population [9].

Meanwhile, vaccine hesitancy, defined by the SAGE Working Group as a delay or refusal to acquire vaccination despite the availability of services, has been listed by WHO as a top 10 global health threat since 2019 [10]. Such reluctance diminishes immunization rates, increasing the susceptibility of individuals and weakening herd immunity, which in turn, raise outbreak risks and may reverse progress against vaccine-preventable diseases [11]. Vaccine hesitancy has increased since the 2009 influenza

pandemic, as suggested in the literature [11]. However, the issue gained much greater attention and urgency during the COVID-19 pandemic, which caused widespread disruptions including prolonged lockdowns, increased misinformation, and significant interruptions to routine immunization services worldwide [12]. These factors have intensified the complexity of vaccine hesitancy, which is widely recognized as a complex and context-dependent issue, with attitudes ranging from full acceptance to outright rejection of vaccines. Notably, the reluctance to get vaccinated can occur even among individuals who acknowledge the importance of immunization [13]. This behavior is driven by several factors, including socioeconomic conditions, parental education, maternal healthcare access, and lack of knowledge. Broader influences encompass awareness, past experiences, perceived vaccination importance, social norms, religious or moral beliefs, distrust in vaccine manufacturers, and concerns regarding risk [13, 14]. Understanding the intricate interplay between the factors affecting vaccine acceptance is essential for public health practitioners, policymakers, and researchers to be able to optimize vaccination coverage and improve immunization strategies.

Such aims can be realized through the vaccination attitudes examination (VAX) scale, a robust instrument introduced by Martin and Petrie in 2017 to evaluate attitudes toward vaccination [15]. The scale has undergone thorough psychometric evaluation across various countries and has been translated into multiple languages, including Spanish, Romanian, Hebrew, Turkish, French, and Colombian [16-21]. Meanwhile, vaccine acceptance is largely influenced by the interplay between perceived disease risk and vaccine safety [22]. Individuals often prioritize avoiding disease exposure over

vaccination, particularly when personal risk is deemed low. Acceptance also varies across nations and cultural contexts, shaped by psychosocial factors and differing views on the advantages of vaccines [23]. These challenges highlight the importance of reliable psychometric tools, such as the VAX scale, in effectively assessing hesitancy-related factors. The VAX scale is designed to measure general attitudes towards vaccination rather than hesitancy related to a specific type of vaccine. This makes the VAX scale a versatile and valuable tool for assessing vaccine attitudes in diverse contexts, including during the COVID-19 pandemic.

On the basis of the studies discussed above, the current research was conducted to bridge the tremendous gap in our understanding of vaccination attitudes, specifically the degree of hesitancy and refusal toward the HBV vaccine and the independent factors influencing these behaviors among Vietnamese adults. By offering evidence-based insights to create focused interventions that successfully address vaccine hesitancy, the findings are anticipated to influence public health programming and initiatives. Furthermore, this study adds to the creation of a reliable and validated measure for assessing general vaccine attitudes, which may be used to assist continuous monitoring and targeted communication efforts to increase vaccination uptake in the Vietnamese population.

Materials and methods

Ethics statement

In this research, informed consent was secured from all participants, all of whom were aged 18 years and above. Before giving their agreement, participants were fully aware of the study's goals, methods, possible dangers, and benefits. Written consent was

acquired and appropriately recorded. The institutional ethics committee examined and approved the study protocol, which included the consent procedure.

Setting and research design

This study was conducted from January to March 2025. A cross-sectional design was implemented following the STROBE (Strengthening the Reporting of Observational Studies in Epidemiology) reporting guidelines.

Respondents were recruited through convenience sampling based on the following criteria: individuals aged 18 years or older, Vietnamese citizens, permanent residents of Ho Chi Minh City, able to understand the questionnaires, and willing to participate in the study. The selection process did not intentionally target any specific group, but individuals who were illiterate, were unable to understand Vietnamese, or provided incomplete survey responses were excluded from the analysis.

The required sample size was determined using the formula $\frac{Z_{a-\alpha/2}^{2}p(1-p)}{d^{2}}$, where the standard normal variate ($Z_{1-\alpha/2}$) is 1.96 at a 95% confidence level (α = 0.05), the expected population proportion (p) is 0.5, and the margin of error (*d*) is 5% [4]. On the basis of this calculation, a minimum of 384 respondents was required to achieve adequate statistical power. To account for an anticipated 10% of incomplete responses, the target sample size was adjusted to 423, which was further expanded to 1,100 respondents to reduce sampling error.

Data collection and instruments

A structured self-administered questionnaire was developed on the basis of similar surveys and the relevant literature on the topic [15, 24, 25] before it was

modified to align with the study's objectives. The questions were refined to eliminate ambiguity, ensuring clarity and conciseness. The questionnaire demonstrated strong internal consistency, with a Cronbach's alpha (α) coefficient of 0.897. Following revisions, it exhibited good reliability and validity.

The final questionnaire consisted of three sections. The first encompassed five questions regarding sociodemographic characteristics, such age, gender, personal income, occupation, and vaccination status. The second section consisted of 10 questions about specific knowledge of HBV vaccines, and the final section comprised 12 questions covering vaccination intention and those in the VAX scale.

Data collection was conducted from January to March 2025 using either Google Forms or printed questionnaires. Our research collaborators approached individuals in public spaces, such as parks, cafés, and supermarkets, to distribute the questionnaire. Participation was voluntary, with informed consent forms acquired before proceeding with the survey. We maintained anonymity and confidentiality by refraining from collecting personally identifiable information. The respondents were told that they were free to withdraw at any time, but no incentives were offered to ensure an unbiased and diverse sample covering individuals of different socioeconomic backgrounds.

Questionnaire

Sociodemographic characteristics

The survey took on average of 20 minutes to complete. The following basic demographic details were derived: age (18–44 years, ≥45 years), gender (coded as 1 = male, 2 = female), personal income (in VND, with average monthly income categorized as follows: <VND 15 million (<US$ 605) and ≥VND 15 million (≥US$ 605),

occupation (with the following codes used: 1 = manual labor, 2 = nonmedical intellectual labor, 3 = healthcare worker, 4 = homemaker/retired/unemployed), vaccination status (coded as 1 = unvaccinated, 2 = vaccinated), and vaccine intention (coded as 1 = refused, 2 = hesitant, 3 = accepted).

Knowledge of the HBV vaccine

Ten items in the vaccine knowledge section were equally weighted and scored dichotomously. Each question had the response options "yes,' "no," and "do not know." The total knowledge score was calculated by summing the number of correct answers, with the maximum possible score being 10. The respondents scoring 5 or higher were classified as having good knowledge of the HBV vaccine, while those scoring below 5 were considered to have poor knowledge [26, 27].

Vaccination intention

A six-point Likert scale was used to assess the respondents' intention to receive the HBV vaccine. They were asked to answer the question "How do you rate your chances of getting the HBV vaccine?" The scale ranged from 1 to 6, with ratings of 1 to 2 indicating unwillingness or refusal, 3 to 4 reflecting hesitancy, and 5 to 6 representing a strong likelihood of vaccine acceptance.

Vaccination attitudes examination scale

The VAX scale [15] consists of 12 items intended to measure general attitudes toward vaccinations. It comprises four three-item subscales: (1) mistrust of vaccine benefits, (2) worries about unforeseen future effects, (3) concerns about commercial profiteering, and (4) preference for natural immunity. The respondents were asked to rate their agreement with each item using a six-point scale ranging from 1 (*totally*

disagree) to 6 (*totally agree*). The level of negative attitudes toward vaccination across all four VAX domains was into three groups: low (1–2), intermediate (3–4), and high (5–6). The final score was obtained by calculating the mean scores for each subscale as well as the mean total score. Low scores indicated positive vaccination views, whereas high scores represented negative perspectives [15].

Data processing and statistical analyses

Data processing

The data processing was grounded in a structured approach to ensure accuracy and reliability. The dataset was cleaned by removing incomplete responses and missing values. For surveys that were completed using printed questionnaires, data entry was conducted manually by trained research assistants, followed by double-checking and verification to minimize entry errors and ensure data quality. Outliers were then identified and excluded to prevent bias in the analysis by using a box plot and the Shapiro–Wilk test.

Statistical analyses

Data from the printed and online survey forms were exported to Microsoft Excel (version 2024) and analyzed using IBM Statistical Package for the Social Sciences (SPSS) for Windows (version 26.0), AMOS, and the psych package in R. Continuous variables were reported as means with standard deviations (*SD*s), while categorical variables were presented as frequencies and percentages. Chi-square (χ^2) tests were carried out to examine differences in vaccine uptake across categorical variables, with statistical significance set at $p < 0.05$. Additionally, multinominal analyses were performed to identify the independent factors associated with vaccine hesitancy and

refusal.

Confirmatory factor analysis (CFA) was performed using maximum likelihood parameter estimates with standard errors and mean-adjusted chi-square tests to validate the structural model. Model fit was evaluated on the basis of several indices with established cut-off criteria: chi-square test, comparative fit index (CFI), Tucker–Lewis index (TLI), root mean square error of approximation (RMSEA), and standardized root mean square residual (SRMR). The internal reliability of the VAX scale was assessed using Cronbach's α and McDonald's omega (ω) coefficients. Measurement invariance across genders (male vs. female) was tested via three nested models with increasing constraints: configural invariance, metric invariance, and scalar invariance. Measurement invariance was confirmed when the change in CFI (ΔCFI) remained below 0.01 [28].

We used multinomial logistic regression to estimate adjusted odds ratios (AOR) and 95% confidence intervals for hesitancy and refusal versus acceptance. Multinomial logistic regression was performed to identify the correlates of vaccine hesitancy and refusal. Vaccine acceptance was used as the reference outcome. The reference category for gender was female; for age, 45 years or older; for occupation, ohmemaker/retired/unemployed; for income, at least 605 USD per month; for vaccination status, not yet vaccinated; for HBV knowledge, good knowledge; and for VAX scores, the low VAX score group.The variables considered theoretically important, including demographic characteristics (e.g., age, sex, and socioeconomic status) and interpersonal factors (e.g., knowledge of hepatitis B vaccination), were entered into the model. Statistical significance was assessed using two-tailed tests, with

$p < 0.05$ considered indicative of a statistically significant association.

Ethical considerations

This study was approved by the Research Ethics Committee of the University of Health Sciences at Vietnam National University in Ho Chi Minh City (IRB No. 01/QD-HDDD). All procedures adhered to the ethical principles outlined in the Declaration of Helsinki. The authors affirm that all procedures in this study complied with the ethical standards of national and institutional committees on human research, as well as the 2008 version of the Helsinki Declaration. The survey was designed to maintain participant privacy, with no personally identifiable information collected. Participation was entirely voluntary and anonymous, with informed consent obtained and documented before the surveys were administered. All collected data were kept confidential and used exclusively for research purposes.

Risk of bias assessment

Quality assessment was performed using the STROBE checklist, which is designed to evaluate the quality and transparency of observational studies. The checklist includes key items related to study design, participants, variables, biases, statistical methods, results, and interpretations [29] (**Appendix**).

Results

Demographic characteristics of the participants

The sociodemographic characteristics of the participants are summarized in **Table 1**. There were a total of 926 participants, among whom 49.6% were male (n = 459), 35.3% (n = 327) were 45 years or older, and over half (64.7%, n = 599) were aged

18 to 44 years. more than 80% (*n* = 676) earned less than US$605 monthly. Occupationally, 42.3% (*n* = 392) were nonmedical intellectual laborers, 34.8% (*n* = 322) were manual laborers, 11.6% (*n* = 107) were healthcare workers, and 11.3% (*n* = 105) were homemakers/retired/unemployed. Among the participants, 41.4% (*n* = 383) were unvaccinated, whereas 58.6% (*n* = 543) had received the HBV vaccine. Regarding vaccination status, 58.6% (n = 543) of participants had been vaccinated, while 41.4% (n = 383) had not. When analyzing vaccination intentions, acceptance was 65.6%, hesitancy 19.3%, and refusal 15.1%.

Table 1. Sociodemographic characteristics of the study population (*N* = 926).

Variable	Number	Percentage (%)
**Gender**		
Male	459	49.6
Female	467	50.4
**Age (years)**		
18-44	599	64.7
≥ 45	327	35.3
**Occupation**		
Manual labor	322	34.8
Non-medical intellectual labor	392	42.3
Healthcare worker	107	11.6
Homemaker/Retired/Unemployed	105	11.3
**Income (US Dollar[1]/month)**		
< 605	676	73.0
≥ 605	250	27.0
**Vaccination status**		
Vaccinated	543	58.6
Not yet	383	41.4
**Vaccination intention among unvaccinated participants (n = 383)**		
Intent to vaccinate	275	71.8
No intention	108	28.2
**General vaccine intentions regardless vaccination status (n=926)**		
Accept	607	65.6
Refusal	140	15.1
Hesitant	179	19.3

[1]US$1 = VND24,786 (*source:* Vietnamese Ministry of Finance: exchange rate for foreign currencies in March 2025)

Table 2 presents respondents' knowledge about the Hepatitis B (HBV) vaccine, including its effectiveness, dosing, duration of protection, necessity of post-vaccination testing, and safety. The data indicate high awareness in certain areas, with 86.4% of participants correctly identifying that an effective vaccine exists (K1) and knowing where to get vaccinated (K10). Additionally, a majority (80.2%) recognized that the vaccine is safe (K9), and 75.2% understood that a complete dose is 95% effective (K3). However, while knowledge about HBV vaccine efficacy and accessibility is good, misconceptions about transmission risks and vaccine safety remain. Only 63.3% correctly answered that a fully recovered patient cannot infect others (K5), suggesting uncertainty about HBV transmission. Furthermore, 31.6% of respondents believed that the HBV vaccine may not cause harmful side effects (K8), highlighting safety concerns that could impact vaccine acceptance.

Table 2. Knowledge about HBV vaccine (n=926)

Items	Varibles	Correct response, n (%)	Incorrect response, n (%)
K1	There is effective vaccine for hepatitis B	800 (86.4)	126 (13.6)
K2	Hepatitis B vaccine dosing 0, 1, 6 months: 3 doses	679 (73.3)	247 (26.7)
K3	Complete dose of Hepatitis B vaccine is 95% effective	696 (75.2)	230 (24.8)
K4	It provides protection for at least 20 years	647 (69.9)	279 (30.1)
K5	A patient who has fully recovered cannot infect others	586 (63.3)	340 (36.7)
K6	Post-Hepatitis B vaccination test is necessary	680 (73.4)	246 (26.6)
K7	For protection against Hepatitis B, one needs antibody titre of >10 IU/m	652 (70.4)	274 (29.6)
K8	The HBV vaccine may cause harmful side effects for recipients	633 (68.4)	293 (31.6)
K9	The HBV vaccine is safe for recipients	743 (80.2)	183 (19.8)

K10	You know where to get the HBV vaccination	800 (86.4)	126 (13.6)

Table 3 presents the VAX scale data. As previously stated, the mean scores ranged from 3.14 (Item 9) to 4.49 (Item 1). The internal dependability of the majority of the items was indicated by the adjusted item-total correlations exceeding 0.40. Items V1, V2, and V3 contributed less to the scale, as their correlations fell below this threshold.

Table 3. Descriptive statistics of the VAX scale

Item	Min	Max	Mean	Std. Deviation	Skewness	Kurtosis	Item-total correlation
V1	1	6	4.49	1.04	-1.02	0.77	0.37
V2	1	6	4.43	0.99	-0.97	0.83	0.38
V3	1	6	4.39	1.02	-0.87	0.44	0.36
V4	1	6	4.35	1.04	-0.81	0.25	0.40
V5	1	6	4.33	1.07	-0.82	0.23	0.46
V6	1	6	4.29	1.05	-0.73	0.04	0.47
V7	1	6	3.29	1.43	-0.14	-1.10	0.53
V8	1	6	3.24	1.45	-0.11	-1.17	0.55
V9	1	6	3.14	1.50	-0.02	-1.27	0.50
V10	1	6	3.80	1.31	-0.58	-0.63	0.62
V11	1	6	3.67	1.38	-0.50	-0.87	0.63
V12	1	6	3.67	1.41	-0.52	-0.90	0.61

Preliminary analysis

Model fit was significant ($\chi^2(32) = 287.40$, $p < 0.001$), indicating that the final model provided a better fit than the intercept-only model. Goodness-of-fit tests showed an adequate fit ($\chi^2(904) = 818.02$, $p = 0.981$), although Pearson χ^2 was significant. Pseudo-R^2 values suggested moderate explanatory power (Cox & Snell = 0.267; Nagelkerke = 0.322; McFadden = 0.176). Likelihood ratio tests indicated that age, occupation, income, vaccination status, HBV knowledge, and three VAX subscales (F1, F3, F4) significantly contributed to the model (all $p < 0.05$). Multicollinearity was

examined using Variance Inflation Factors (VIF), all predictors showed VIF values ranging from 1.34 to 2.10, these results indicate that multicollinearity was not a concern in the regression model.

Parallel factor analysis confirmed the validity of the four-factor structure of the VAX scale, with a strong fit (χ^2(24, N = 926) at 41.61, a p = 0.01, a TLI = 0.99, and an RMSEA = 0.028 (95% confidence interval [CI]: 0.01–0.04). The model explained 70.04% of the cumulative variance in the data. A KMO = 0.85 indicated sampling adequacy, and Bartlett's test showed substantial item correlations (χ^2(66, N = 926) = 7050.23, p < .001), permitting component analysis (see Table 4).

Table 4. Factor loadings, explained variances, and reliability.

Items	F1	F2	F3	F4	Uniqueness (PAF)
V1	-0.010				0.419
V2	-0.012				0.284
V3	0.034				0.315
V4		0.318			0.414
V5		0.090			0.345
V6		-0.106			0.419
V7			-0.053		0.209
V8			0.012		0.190
V9			0.073		0.207
V10				0.159	0.422
V11				-0.058	0.115
V12				-0.058	0.213
Cronbach's alpha (α)	0.848	0.812	0.919	0.890	
McDonald's omega (ω)	0.850	0.820	0.920	0.900	

F1 = mistrust of vaccine benefit; F2 = worries about unforeseen future effects; F3 = concerns about commercial profiteering; F4 = preference for natural immunity.

The CFA based on the maximum likelihood χ^2 S-B estimator for nonnormal data validated the VAX scale's four-component structure. The results suggested a strong fit—χ^2(49, N = 926) = 198.454, p < 0.001, CFI = 0.98, TLI = 0.97, RMSEA = 0.06 (90% CI: 0.05–0.07); internal consistency was satisfactory, with Cronbach's α ranging from 0.812 to 0.919 and McDonald's ω ranging from 0.820 to 0.920 across subscales,

demonstrating satisfactory internal consistency. Most of the items significantly contributed to latent components, but those with high uniqueness values (>0.40) pointed to weaker correlations or ambiguity.

The CFA validation of the VAX scale structure also verified the scale's constructs (see Fig 1).

Fig 1. CFA results: Path diagram and standardized estimated parameters for the Vietnamese VAX scale.

The interfactor correlations were inconsistent, with the strongest correlation between F1 and F4 (r = 0.77) and the weakest between F1 and F2 (r = 0.03), indicating a variety of attitudinal dimensions on vaccine attitudes. Measurement invariance across genders showed no differences, implying that the male and female participants understood the VAX scale in the same manner (see Supplementary Table 1). Strong associations, such as those between F1–F2 (r = 0.61, p = 0.01) and VAX_Total-F4(r = 0.782, p = 0.01), indicated F4's substantial contribution to unfavorable vaccination beliefs, as evidenced by the Pearson correlations between the subscales and VAX_Total.

Age was associated with favorable vaccination views, reflecting decreased mistrust (r = –0.10, p < 0.01), worries about future effects (r = –0.09, p = 0.05), profiteering concern (r = –0.07, p < *0.01*) and concern about commercial profiteering (r = –0.07, p < 0.05). Employed individuals exhibited increased vaccine hesitancy compared with unemployed/retired individuals, even under negative associations across categories (e.g., mistrust r = –0.11, p = 0.01). Getting vaccinated was negatively associated with VAX_Total (r = –0.15, p = 0.01) and particular worries (e.g., profiteering: r = –0.14, p = 0.01), reflecting favorable attitudes among those receiving

the vaccine. The intention to receive vaccination was substantially linked to income ($r = 0.12$, $p < 0.01$) and VAX_Total ($r = 0.14$, $p < 0.01$), albeit with respondents exhibiting mild suspicion and fears (e.g., F1: $r = 0.18$, $p = 0.01$). Knowledge of HBV vaccination was positively correlated with F1 ($r = 0.23$, $p = 0.01$), F2 ($r = 0.19$, $p = 0.01$), F4 ($r = 0.11$, $p = 0.01$), and VAX_Total ($r = 0.19$, $p = 0.01$), denoting that strong knowledge is related to increased fears and cautious attitudes toward vaccination. These results show the complex interaction between demographic attributes, knowledge of vaccines, and vaccination attitudes in the Vietnamese context (see Fig 2).

Fig 2. Perceptions of vaccination.

Among the respondents highly suspicious of vaccine benefits (high group), 63.3% expressed concerns about vaccine efficacy compared with those exhibiting low suspicion (low group, 2.1%). Concerns regarding unexpected consequences were more frequent in the high group (57.7%) than in the low group (1.6%). Commercial profiteering concerns were fairly distributed across the sample, with 46.3% of participants in the intermediate group, 27.8%, and 25.9% exhibiting moderately strong, low, and strong concerns, respectively. Ultimately, the moderate group (47.3%) favored natural immunity the most, followed by the high (38.9%) and low (13.8%) groups. Of the participants, 65.6% were ready to receive vaccination, 19.3% remained dubious, and 15.1% were reluctant.

Comparison of intention on the basis of VAX subscale mean scores

A one-way ANOVA was conducted to compare the mean scores on the VAX subscales of the refusal, hesitant, and willing groups for HBV vaccination. With modest effect sizes, the willing group scored far higher on F1 (mistrust of vaccine advantages) and F2 (worries about future consequences) than the hesitant or refusal groups ($p <$

0.001). With a $p < 0.001$, the refusal group scored higher on F4 (preference for natural immunity). The groups' F3 (commercial profiteering concerns) scores had no significant differences (see Supplementary Table 2).

Predictors of HBV vaccine hesitancy and refusal

The results indicate that individuals with a high level of mistrust were 85.3% less likely to refuse the vaccine but 78.8% more likely to exhibit hesitancy compared to those with low level of mistrust. Similarly, those with a high level of worries were 82.2% less likely to refuse the vaccine and 85.0% less likely to be hesitant than individuals with low levels of worries. Among participants with a high level of concern about commercial profiteering, vaccine refusal decreased by 59.3%, while hesitancy increased by 41.6% compared to those with low level of concern. In contrast, individuals with a high preference for natural immunity were more than twice as likely to refuse the vaccine and 84.4% less likely to be hesitant compared to those with a low preference (see Fig 3).

Fig 3. Predictors of HBV vaccine hesitancy and refusal using multinominal logistic regression analysis.

Predictors of refusal to vaccinate against HBV

We found that the refusal to receive the HBV vaccine was higher among the participants with personal incomes of less than US$605 per month (AOR = 1.82, 95% CI: 1.08–3.07) and those with poor knowledge of the HBV vaccine (AOR = 2.42, 95% CI: 1.30–4.53). Such refusal was lower among the healthcare workers (AOR = 0.23, 95% CI: 0.07–0.72) and participants aged 18 to 44 years (AOR = 0.64, 95% CI: 0.41–0.99). The refusal to get vaccinated was also greater among the participants who strongly favored natural immunity (AOR = 3.06, 95% CI: 1.37–6.84). It was lower

among the respondents who were substantially concerned about commercial profiteering (AOR = 0.41, 95% CI: 0.23–0.73) but lower among those who mistrusted vaccine benefits (AOR = 0.15, 95% CI: 0.03–0.86).

Predictors of hesitancy to vaccinate against HBV

We identified the predictors of hesitancy and acceptance of the HBV vaccine. As mentioned earlier, hesitancy was observed in the participants with personal incomes of less than US$605 dollars (AOR = 1.66, 95% CI: 1.06–2.60) and those with poor knowledge of the HBV vaccine (AOR = 3.39, 95% CI: 2.01–5.71). Such reluctance was greater among the respondents displaying moderate concerns about commercial profiteering (AOR = 2.14, 95% CI: 1.16–3.95). In contrast, hesitancy was lower among the participants highly preferring natural immunity (AOR = 0.16, 95% CI: 0.07–0.37) (**Fig 3**).

Discussion

Evidence points to although most people are aware of the HBV vaccination's efficacy and accessibility, there are still considerable misconceptions about transmission hazards and vaccine safety. This knowledge gap could lead to increased anxiety as individuals become aware more about the vaccine but are still unsure about possible adverse effects or misjudge how HBV spreads. These misunderstandings may impact their choice for different ways to protect themselves from HBV infection. Furthermore, there are some pieces of information that deal with technical aspects and side effects of vaccines (such as antibody titers and post-vaccination testing), which can be complicated and hard to grasp, leading people to misunderstand or be confused. It is possible that individuals who have a deeper acquaintance with these features will

develop a stronger awareness of rare adverse effects and the complexity involved in vaccine-induced immunity, which may lead to increased concerns about unanticipated future implications. The current pattern indicates a measured reluctance to trust without first gathering relevant information.

The study demonstrates that the mean VAX scores were 3.0 or higher, suggesting moderate to high skepticism toward vaccination. However, vaccine acceptance remained reasonably high, at 65.6%. This discrepancy implies that vaccination acceptance is not directly predicted by attitudes as assessed by the VAX scale. This could be explained by a number of factors, including perceived dangers of not getting vaccinated, the immediacy of the health threat, cultural and social influences, and the accessibility and affordability of vaccines. For instance, many people decided for vaccination during the COVID-19 pandemic in spite of concerns over its efficacy or safety. It is crucial to remember that the VAX scale evaluates skepticism or trust in vaccines rather than acceptance of them. Consequently, vaccination adoption is impacted by a complex interaction of factors beyond attitude alone, even when VAX scores indicate widespread hesitation.

Our findings support the factorial structure of the VAX scale: mistrust of vaccine benefits (F1), worries about unforeseen future effects (F2), concerns about commercial profiteering (F3), and preference for natural immunity (F4). This four-factor solution aligns with the original VAX structure proposed by Martin and Petrie [15] and is consistent with findings from other cross-cultural validations, such as those conducted in Romania [17], Italy [25], and Turkey [19]. The results also support the reliability and validity of the VAX scale in the Vietnamese context, a culturally distinct setting,

suggesting that the scale maintains its psychometric robustness across diverse cultural backgrounds.

Vaccine hesitancy remains one of the major barriers to the elimination of hepatitis B. Numerous studies have assessed the acceptance of the HBV vaccine and have discovered a reluctance to receive vaccination, even in countries with high immunization rates. Common concerns include potential side effects (e.g., multiple sclerosis) [30-32], a perceived low risk of infection in newborns, and a greater fear of vaccine-related harm, such as infant death, than of the disease itself[33-35]. Other contributing factors are the lack of strong recommendations from healthcare providers [36-38], limited media communication [39, 40], low education levels, insufficient knowledge about HBV infection risks [41], and a general perception that the vaccine is unnecessary [42], among other issues.

In an Italian sample, high levels of vaccine hesitancy were observed among healthcare workers with low educational attainment, with such hesitancy frequently associated with fears of developing multiple sclerosis, as well as beliefs linking aluminum-based adjuvants in vaccines to Alzheimer's disease [43]. Racial or ethnic disparities in vaccine acceptance have also been reported. Vulnerable populations, such as pregnant women [44], hemodialysis patients [45], people living with HIV [46], and men who have sex with men, are particularly affected by these attitudes [47].

The configuration, metric, and scalar levels of measurement invariance were all validated in the examination of the factorial structure during the gender-based multigroup analyses. The consistent underlying structure, item loadings, and intercepts across gender groups of the VAX scale validated its effectiveness in evaluating vaccine

attitudes equivalently for men and women. Consistent with results of Bruno et al., our findings confirmed the suitability of the scale for gender-based comparisons [25]. Such uniformity and cross-group comparability offer strong support for further studies on gender variations in self-reported vaccine reluctance with conceptual consistency.

Our study found a 65.6% acceptance rate of hepatitis B vaccination, which is lower than that found in the Indonesian context (84.5%), where many participants were healthcare professionals with better awareness of health and access to insurance coverage of vaccination expenses [48]. On the other hand, our rate exceeds that derived in a Chinese study, in which only 20.67% of unvaccinated individuals regarded the hepatitis B vaccine necessary, mostly due to safety and efficacy matters [49]. These cross-country variations underscore the influence of sociocultural, informational, and systemic factors—such as health knowledge, access to vaccination services, and trust in healthcare institutions—that shape vaccine acceptance. Understanding these differences is critical for developing tailored public health interventions. Our findings contribute to the literature by highlighting how contextual factors impact vaccine tolerance across diverse populations.

With a projected population of 8.9 million by mid-2023, Ho Chi Minh City, Vietnam's most populous and economically vibrant city, offers a unique socioeconomic environment that might help explain its high hepatitis B vaccination rates [50]. As a major financial, commercial, educational, and technological hub, the city has undergone rapid urbanization and significant healthcare improvements through substantial investments in infrastructure and services. These developments have enhanced public access to healthcare information and services, increasing awareness of the importance

of vaccination. Additionally, coordinated efforts among local authorities, NGOs, and healthcare providers have been pivotal in building public trust and encouraging participation in vaccination campaigns.

Despite these favorable conditions and ongoing public health initiatives, our findings reveal that the hepatitis B vaccination acceptance rate is lower than might be expected for a city with such advancements. This discrepancy suggests the presence of persistent barriers, such as vaccine hesitancy, misinformation, or unequal access among certain population groups. These factors highlight the complexity of vaccine acceptance and indicate that socioeconomic progress and campaign efforts alone may not be sufficient to achieve optimal vaccination coverage. Therefore, a deeper understanding of these contextual challenges is necessary to tailor more effective interventions and policies to increase vaccine uptake in Ho Chi Minh City.

Relationship between demographic characteristics and vaccine hesitancy and refusal

Contrary to earlier studies showing gender differences in vaccine uptake, gender was not a significant predictor of hepatitis B vaccine refusal in the present research. For example, a study among Laotian Americans found that women are far more likely to receive vaccination (adjusted OR = 4.0, 95% CI: 1.2–19), whereas men tend to believe in the preventability and treatability of HBV [51]. Similarly, studies on healthcare workers in five developing nations found that female workers are predisposed to complete the hepatitis B vaccine series, albeit the findings varied by setting [52]. Gender variations were also discovered in relation to other vaccines, with women found to be more sensitive to side effects or more strongly affected by issues such as pregnancy

[53]. These contradictions imply that sociocultural standards, healthcare availability, and vocational positions influence the effects of gender on vaccination acceptance. The absence of a gender effect in our study may reflect specific sociocultural factors in Vietnam. Traditional gender roles, coupled with relatively equal healthcare access and vocational opportunities in urban settings, might reduce gender disparities in vaccination acceptance. This contrasts with findings from other contexts, highlighting the importance of localized sociocultural and structural influences.

In this study, income level was a major determinant of the reluctance or refusal to get vaccinated. Low earners were around 1.8 times more likely than high-income individuals to refuse or hesitate with respect to vaccination. This corresponds with prior research on social inequalities in immunization rates, such as studies in rural China highlighting poverty as a primary factor in hepatitis B vaccination disparities [54]. In Vietnam, economic hardship intersects with limited access to trustworthy health information and lower institutional trust, which may intensify vaccine hesitancy among low-income groups. Moreover, unlike some low-income countries where increased government vaccine delivery effectively boosts coverage, structural and sociocultural barriers in Vietnam require tailored public health strategies. These contextual and population-specific factors not only reinforce the global pattern linking low income to vaccine hesitancy but also emphasize the need for interventions addressing both financial and informational obstacles to improve vaccination uptake in Vietnam.

In interpreting the findings on vaccine hesitancy and refusal, it is important to clarify how the VAX variable captures attitudes toward hepatitis B vaccination. The VAX measure reflects participants' general views about vaccination, regardless of

whether they have previously been vaccinated. Therefore, even individuals who have already received one or more doses may still report "accept," "hesitant," or "refusal" depending on their current attitude. Based on this framework, the refusal group likely includes those who do not intend to vaccinate (108 participants) as well as some previously vaccinated individuals who could refuse additional doses or oppose recommending vaccination. The hesitant group may include individuals who have initiated vaccination but remain uncertain about completing the series. Meanwhile, the accept group consists of those who intend to vaccinate (275 participants) and those who are already vaccinated. Distinguishing between actual vaccination status and future vaccination intention helps resolve the seeming inconsistency in the results and provides a clearer understanding of how attitudes toward vaccination vary across demographic groups.

Individuals with inadequate knowledge regarding HBV vaccination exhibit vaccine refusal or hesitancy that is two to three times greater than that among those with a sufficient understanding of the issue. Research conducted in Indonesia uncovered that a core obstacle to immunization is ignorance of its advantages [55]. Similar trends were noted in Vietnam and Uganda, where little knowledge of HBV and its transmission diminishes the acceptability of vaccination [56, 57]. Efforts should thus prioritize low-awareness communities, with easily available, culturally acceptable resources used as part of campaigns. Particularly in rural or underprivileged regions, tools such as illustrated fliers, pamphlets, and short educational movies with clear graphics should be extensively available in local health clinics, schools, marketplaces, and mobile health units [58]. Involving local healthcare professionals, leaders, and influencers can also

help build confidence and increase community participation. These strategies can increase health literacy, thereby reducing hesitation and encouraging the equitable distribution of HBV vaccines.

In this study, psychological and attitudinal factors clearly affected HBV vaccination reluctance and rejection. First, individuals with a high level of mistrust in vaccine benefits were significantly less likely to refuse the vaccine (by 84.3%) but more likely to exhibit hesitancy (by 78.8%) compared to those with lower levels of mistrust. This suggests that mistrust may not necessarily lead to outright rejection but rather to uncertainty and cautious indecision. In other words, mistrustful individuals may delay or question vaccination without completely refusing it, Kollamparambil et al. (2023), for example, demonstrated that vaccine distrust was the strongest predictor of vaccine hesitancy in South Africa, highlighting that individuals with high levels of distrust are more likely to vacillate or postpone vaccination than to reject it completely [59].

In the context of Vietnam, where hepatitis B infection remains highly prevalent, vaccination is recognized as an essential preventive measure. Therefore, even though some individuals express greater worries, they appear less likely to refuse or hesitate about receiving the vaccine. This may reflect a pragmatic understanding that the benefits of vaccination outweigh potential risks, particularly given the serious consequences of HBV infection. In other words, despite existing worries, the perceived necessity and urgency of protection against HBV encourage people to proceed with vaccination rather than avoid it.

Regarding concerns about commercial profiteering, participants who believed that vaccine promotion was mainly motivated by business or profit were less likely to

completely refuse the vaccine but more likely to hesitate. Their hesitation may come from a lack of trust rather than opposition to the vaccine itself. In practice, this indicates that improving transparency in vaccine distribution, providing clear information about pricing, and emphasizing public health benefits instead of commercial aspects could help reduce such doubts and encourage higher vaccine acceptance.

In contrast, individuals with a strong preference for natural immunity were more than twice as likely to refuse vaccination and 84.4% less likely to be hesitant. This suggests that those who believe natural infection provides stronger or more "authentic" protection than vaccines tend to make firm decisions against vaccination rather than remain uncertain. In contexts like Vietnam, where public awareness of HBV risk is increasing, such attitudes may pose a challenge for vaccine promotion because they represent a deep-seated worldview rather than a simple lack of information. Therefore, public health communication should not only address misinformation but also emphasize how vaccination safely strengthens, rather than replaces, natural immunity.

Underlining the intricacy of hesitation, a Turkish study among healthcare professionals identified mistrust, fears of injury or autism, media disinformation, and religious concerns as grounds for rejection [60]. In the present work, the participants displaying considerable concerns about vaccines were 60% less likely to decline vaccination, while those with moderate anxiety about this issue were 1.5 times more likely to hesitate[61]. Similar patterns were observed among individuals expressing concerns about commercial profiteering. Furthermore, our findings suggest that individuals with strong opinions tend to make more definitive decisions regarding vaccination, whether acceptance or refusal, compared to those with less pronounced

views [62]. This highlights that vaccine decision-making is influenced not only by the presence of concerns but also by the clarity and strength of personal attitudes.

The respondents who preferred natural immunity were three times more likely to reject HBV vaccination but were 85% less likely to display hesitation [63-65]. Consistent with the results derived in the context of France [31], our findings demonstrated that concerns about unanticipated future impacts had no appreciable influence on rejection or hesitation. These outcomes emphasize the importance of addressing the psychological as well as socioeconomic factors of vaccination behavior. Public health messages should be customized to address issues such as commercial uncertainty or natural immunity preferences to build trust by means of evidence-based, sympathetic communication.

Vaccine refusal and hesitation ultimately result from a confluence of psychological and sociodemographic elements. The results can inform future studies and enable public health experts to create sensible plans. Important activities include addressing personal issues, offering correct information on vaccine safety and efficacy, fighting false information, and involving medical professionals in education campaigns. Vaccine acceptance and confidence in healthcare systems can be improved even more by customizing communication to match community-specific issues of age, income, and education levels.

Strengths and limitations of the study

This study presents several strengths. Notably, the VAX scale's reliability and validity in the Vietnamese context are guaranteed by the fact that it is the first to do a psychometric validation of the scale among Vietnamese adults, confirming its original

four-factor structure and proving measurement invariance across genders. The study also finds actionable correlations of vaccine reluctance and refusal, such as income, hepatitis B vaccination knowledge, natural immunity beliefs, and commercial interest concerns. Last but not least, these results establish precise programmatic goals to raise adult hepatitis B vaccination rates, giving legislators important direction for creating efficient vaccination laws, improving program execution, and customizing public health initiatives. Limitations include the results' applicability to the larger Vietnamese population, particularly rural populations, may be limited by the use of convenience sampling that was concentrated on metropolitan regions. Using self-reported data could lead to social desirability bias or reporting bias. Furthermore, no causal conclusions can be made about the associations found because of the cross-sectional study methodology. Lastly, residual confounding from unmeasured factors cannot be completely ruled out even after controlling for a number of covariates.Future research should adopt longitudinal designs, diversify data collection methods, and randomize item order to reduce consistency bias from consecutive item presentation.

Conclusion

Despite favorable conditions and ongoing public health efforts in Ho Chi Minh City, the acceptance of the hepatitis B vaccine remains lower than expected, indicating the presence of persistent barriers such as vaccine hesitancy, misinformation, and unequal access. Our research emphasizes that vaccine decision-making is influenced by the strength and lucidity of personal attitudes toward vaccination, in addition to existing concerns. The VAX scale has been demonstrated to be a valuable instrument for accurately evaluating these attitudes within the Vietnamese population in this context.

By identifying specific factors contributing to hesitancy through this scale, public health interventions can be better tailored to address these challenges and ultimately improve vaccination rates.

Abbreviation

HBV*:* Hepatitis B Virus**, CDC*:*** Centers for Disease Control and Prevention**, CFA**: Confirmatory factor analysis**, DALYs:** disability-adjusted life years, **HCV:** Hepatitis C Virus, , ***HCMC:*** Ho Chi Minh City**, NGOs**: non-governmental organizations, ***OR:*** Odds Ratio, **KAP*:*** Knowledge Attitude and Practice**, SES*:*** Socioeconomic status, **VAX:** Vaccination Attitudes Examination, **WHO*:*** World Health Organization.

Supplementary materials

Supplement Table 1. Test of the invariance of VAX scale across gender
Supplement Table 2. One-way Anova results of the difference among the total score of the VAX and intention to get HBV vaccine.
Supplement Table 3. Pearson's correlation, means, standard deviation (SD) for VAX subscales, and total score

Author contributions

T.N. contributed to conception the research idea and creating the research design T.H and N.H contributed to the acquisition of data and analysis. C.L. and T.N. contributed to drafting the manuscript's intellectual content. All authors had full access to all the data in the study and take the responsibility for the integrity of the data and the accuracy of the data analysis. All the authors have read and agree to the published version of the manuscript.

Financial Disclosure

This research is funded by Vietnam National University Ho Chi Minh City (VNU-HCM) under grant number 36-2025-44-02. The funders had no role in study design, data collection and analysis, decision to publish, or preparation of the manuscript

Institutional Review Board Statement: This study was conducted in accordance with the Declaration of Helsinki and approved by the Institutional Ethics Committee of University of Health Sciences, Vietnam National University (Ethics No. IRB No. 01/QD-HDDD). No Animals were used in the studies that were the basis of this research. All human procedures were carried out in accordance with the ethical standards of the committee responsible for human experimentation (institutional and national) and with the Helsinki Declaration of 1975, as revised in 2013.

Informed Consent Statement: Informed consent was obtained from all subjects involved in the study.

Data availability Statement: All relevant data are within the paper and its Supporting Information files.

Acknowledgments

We thank the respondents from pharmacists who gave us valuable inputs in the questionnaire development phase that helped us to improve the questionnaire and adapt it for the local population.

Conflict of Interest: The authors declare no conflict of interest.

APPENDIX

Appendix 1. STROBE Statement- Checklist of items that should be included in reports of *cross-sectional studies*

REFERENCES

1. Organization WH. Hepatitis B 2024 [cited 2024 Oct 2]. Available from: https://www.who.int/news-room/fact-sheets/detail/hepatitis-b.
2. Devarbhavi H, Asrani SK, Arab JP, Nartey YA, Pose E, Kamath PS. Global burden of liver disease: 2023 update. Journal of hepatology. 2023;79(2):516-37.
3. Pham TND, Le DH, Dao DVB, Phan LTB, Pham TTT, Nguyen TB, et al. Establishing baseline framework for hepatitis B virus micro-elimination in Ho Chi Minh City, Vietnam - A community-based seroprevalence study. Lancet Reg Health West Pac. 2023;30:100620.
4. Global prevalence, treatment, and prevention of hepatitis B virus infection in 2016: a modelling study. Lancet Gastroenterol Hepatol. 2018;3(6):383-403.
5. Nguyen-Dinh SH, Do A, Pham TND, Dao DY, Nguy TN, Chen MS, Jr. High burden of hepatocellular carcinoma and viral hepatitis in Southern and Central Vietnam: Experience of a large tertiary referral center, 2010 to 2016. World J Hepatol. 2018;10(1):116-23.
6. de Villiers MJ, Nayagam S, Hallett TB. The impact of the timely birth dose vaccine on the global elimination of hepatitis B. Nature communications. 2021;12(1):6223.
7. El-Sayed MH, Feld JJ. Vaccination at the forefront of the fight against hepatitis B and C. Nature Reviews Gastroenterology & Hepatology. 2022;19(2):87-8.
8. Komada K, Ichimura Y, Shimada M, Funato M, Do HT, Le HX, et al. Impact of hepatitis B vaccination programs in Vietnam evaluated by estimating HBsAg prevalence. Journal of Virus Eradication. 2022;8(4):100309.
9. Health VMo. Issuance of the national action plan for viral hepatitis prevention and management 2021-2025. 4531/QD-BYT. 2021.
10. Organization WH. Ten threats to global health in 2019 2019 [Available from: https://www.who.int/news-room/spotlight/ten-threats-to-global-health-in-2019?utm_source=chatgpt.com.
11. Larson HJ, Jarrett C, Eckersberger E, Smith DM, Paterson P. Understanding vaccine hesitancy around vaccines and vaccination from a global perspective: a systematic review of published literature, 2007–2012. Vaccine. 2014;32(19):2150-9.
12. Causey K, Fullman N, Sorensen RJD, Galles NC, Zheng P, Aravkin A, et al. Estimating global and regional disruptions to routine childhood vaccine coverage during the COVID-19 pandemic in 2020: a modelling study. Lancet. 2021;398(10299):522-34.
13. Jiang H, Wei C. How to address vaccine hesitancy? Lessons from national hepatitis B immunization program in China. Frontiers in Public Health. 2024;12:1286801.
14. Yaqub O, Castle-Clarke S, Sevdalis N, Chataway J. Attitudes to vaccination: a critical review. Soc Sci Med. 2014;112:1-11.

15. Martin LR, Petrie KJ. Understanding the dimensions of anti-vaccination attitudes: The vaccination attitudes examination (VAX) scale. Annals of Behavioral Medicine. 2017;51(5):652-60.
16. Paredes B, Cárdaba MÁ, Cuesta U, Martinez L. Validity of the Spanish version of the vaccination attitudes examination scale. Vaccines. 2021;9(11):1237.
17. Huza G. The psychometric properties of a Romanian version of the vaccination attitudes examination (VAX) scale. International Journal of HIV/AIDS Prevention, Education and Behavioural Science. 2020;6(1):25.
18. Shacham M, Greenblatt-Kimron L, Hamama-Raz Y, Martin LR, Peleg O, Ben-Ezra M, Mijiritsky E. Increased COVID-19 Vaccination Hesitancy and Health Awareness amid COVID-19 Vaccinations Programs in Israel. Int J Environ Res Public Health. 2021;18(7).
19. Yildiz E, Gungormus Z, Dayapoglu N. Assessment of validity and reliability of the turkish version of the vaccination attitudes examination (VAX) scale. Int J Caring Sci. 2021;14(1):261-9.
20. Eisenblaetter M, Madiouni C, Laraki Y, Capdevielle D, Raffard S. Adaptation and Validation of a French Version of the Vaccination Attitudes Examination (VAX) Scale. Vaccines (Basel). 2023;11(5).
21. Espejo B, Martín-Carbonell M, Romero-Acosta KC, Fernández-Daza M, Paternina Y. Evidence of Validity and Measurement Invariance by Gender of the Vaccination Attitudes Examination (VAX) Scale in Colombian University Students. J Clin Med. 2022;11(16).
22. Kim J, Han K, Chung SJ, Kim C. Psychometric validation of the Korean versions of the Vaccine Hesitancy Scale and Vaccination Attitudes Examination Scale. Vaccine. 2023;41(32):4685-92.
23. Cheng L, Kong J, Xie X, Zhang F. A psychometric assessment of a novel scale for evaluating vaccination attitudes amidst a major public health crisis. Scientific Reports. 2024;14(1):10250.
24. Biella M, Orrù G, Ciacchini R, Conversano C, Marazziti D, Gemignani A. Anti-Vaccination Attitude and Vaccination Intentions Against Covid-19: A Retrospective Cross-Sectional Study Investigating the Role of Media Consumption. Clin Neuropsychiatry. 2023;20(4):252-63.
25. Bruno F, Laganà V, Pistininzi R, Tarantino F, Martin L, Servidio R. Validation and psychometric properties of the Italian Vaccination Attitudes Examination (VAX-I) scale. Curr Psychol. 2022:1-11.
26. Shrestha DB, Khadka M, Khadka M, Subedi P, Pokharel S, Thapa BB. Hepatitis B vaccination status and knowledge, attitude, and practice regarding Hepatitis B among preclinical medical students of a medical college in Nepal. PloS one. 2020;15(11):e0242658.
27. Bhattarai S, Gyawali M, Sapkota S, Karki D, Lamsal S. Knowledge, attitude and practice of hepatitis B vaccination among clinical medical students at a medical college in Nepal. Europasian Journal of Medical Sciences. 2020;2(2):17-23.
28. Cheung GW, Rensvold RB. Evaluating goodness-of-fit indexes for testing measurement invariance. Structural equation modeling. 2002;9(2):233-55.
29. Von Elm E, Altman DG, Egger M, Pocock SJ, Gøtzsche PC, Vandenbroucke JP. The Strengthening the Reporting of Observational Studies in Epidemiology (STROBE) statement: guidelines for reporting observational studies. The lancet. 2007;370(9596):1453-7.
30. Gobert C, Semaille P, Van der Schueren T, Verger P, Dauby N. Prevalence and determinants of vaccine hesitancy and vaccines recommendation discrepancies among general practitioners in French-speaking parts of Belgium. Vaccines. 2021;9(7):771.
31. Moyroud L, Hustache S, Goirand L, Hauzanneau M, Epaulard O. Negative perceptions of hepatitis B vaccination among attendees of an urban free testing center for sexually

transmitted infections in France. Human Vaccines & Immunotherapeutics. 2017;13(5):998-1004.
32. Erdoğan F. Could vaccine hesitancy be a global health threat after pandemics?: A observational study at early phase of COVID-19 pandemic in 2020. 2022.
33. Gilmartin CE, Daley AJ, Leung L. The hepatitis B birth-dose immunisation: Exploring parental refusal. Australian and New Zealand Journal of Obstetrics and Gynaecology. 2020;60(1):93-100.
34. Rozbroj T, Lyons A, Lucke J. Vaccine-hesitant and vaccine-refusing parents' reflections on the way parenthood changed their attitudes to vaccination. Journal of Community Health. 2020;45(1):63-72.
35. Yu W, Liu D, Zheng J, Liu Y, An Z, Rodewald L, et al. Loss of confidence in vaccines following media reports of infant deaths after hepatitis B vaccination in China. International Journal of Epidemiology. 2016;45(2):441-9.
36. Agrinier N, Le Maréchal M, Fressard L, Verger P, Pulcini C. Discrepancies between general practitioners' vaccination recommendations for their patients and practices for their children. Clinical Microbiology and Infection. 2017;23(5):311-7.
37. Killian M, Detoc M, Berthelot P, Charles R, Gagneux-Brunon A, Lucht F, et al. Vaccine hesitancy among general practitioners: evaluation and comparison of their immunisation practice for themselves, their patients and their children. European Journal of Clinical Microbiology & Infectious Diseases. 2016;35:1837-43.
38. Verger P, Fressard L, Collange F, Gautier A, Jestin C, Launay O, et al. Vaccine hesitancy among general practitioners and its determinants during controversies: a national cross-sectional survey in France. EBioMedicine. 2015;2(8):891-7.
39. Bar-Lev S, Reichman S, Barnett-Itzhaki Z. Prediction of vaccine hesitancy based on social media traffic among Israeli parents using machine learning strategies. Israel journal of health policy research. 2021;10:1-8.
40. Powell GA, Zinszer K, Verma A, Bahk C, Madoff L, Brownstein J, Buckeridge D. Media content about vaccines in the United States and Canada, 2012–2014: An analysis using data from the Vaccine Sentimeter. Vaccine. 2016;34(50):6229-35.
41. Afolabi IB, Aremu AB, Maidoki LA, Atulomah NO. Predictors of Hepatitis B Virus Infection Vaccine Hesitancy Among Pregnant Women Attending Antenatal Care at Lubaga Hospital, Kampala, Uganda. Int J Womens Health. 2022;14:1093-104.
42. Francisco PM, Donalisio MR, Gabriel Fde J, Barros MB. Hepatitis B vaccination in adolescents living in Campinas, São Paulo, Brazil. Rev Bras Epidemiol. 2015;18(3):552-67.
43. de Araújo TM, Souza FO, Pinho PS, Werneck GL. Beliefs and Sociodemographic and Occupational Factors Associated with Vaccine Hesitancy among Health Workers. Vaccines (Basel). 2022;10(12).
44. Silva T, Gomes CS, Carmo ASD, Mendes LL, Rezende EM, Velasquez-Melendez G, Matozinhos FP. Spatial analysis of vaccination against Hepatitis B in pregnant women in an urban Brazilian area. Cien Saude Colet. 2021;26(3):1173-82.
45. Guimarães MNC, Facincani T, Santos SSD. Hepatitis B status in hemodialysis patients. Arq Gastroenterol. 2017;54(4):356-8.
46. Pinto Neto L, Vieira JV, Ronchi NR. Vaccination coverage in a cohort of HIV-infected patients receiving care at an AIDS outpatient clinic in Espírito Santo, Brazil. Braz J Infect Dis. 2017;21(5):515-9.
47. Xiridou M, Adam P, Meiberg A, Visser M, Matser A, de Wit J, Op de Coul E. The impact of the COVID-19 pandemic on hepatitis B virus vaccination and transmission among men who have sex with men: A mathematical modelling study. Vaccine. 2022;40(33):4889-96.

48. Machmud PB, Mikolajczyk R, Gottschick C. Understanding hepatitis B vaccination willingness in the adult population in Indonesia: a survey among outpatient and healthcare workers in community health centers. Journal of Public Health. 2023;31(12):1969-80.
49. Bai X, Chen L, Liu X, Tong Y, Wang L, Zhou M, et al. Adult hepatitis B virus vaccination coverage in China from 2011 to 2021: a systematic review. Vaccines. 2022;10(6):900.
50. Vietnam GSOo. Statistical Yearbook of Vietnam 2023. 2023.
51. Akosionu O, Virnig B, Call KT, Yuan J-M, Chanthanouvong S, Nguyen RH. The impact of gender differences in attitudes and beliefs concerning HBV vaccination and screening in the Lao community. Journal of immigrant and minority health. 2016;18:277-81.
52. Duodu PA, Darkwah E, Agbadi P, Duah HO, Nutor JJ. Prevalence and geo-clinicodemographic factors associated with hepatitis B vaccination among healthcare workers in five developing countries. BMC infectious diseases. 2022;22(1):599.
53. Dangerfeild K. Why Women Are Bearing the Brunt of COVID-19 Vaccine Side Effects.
54. Zhu D, Guo N, Wang J, Nicholas S, Wang Z, Zhang G, et al. Socioeconomic inequality in Hepatitis B vaccination of rural adults in China. Hum Vaccin Immunother. 2018;14(2):464-70.
55. Machmud PB, Führer A, Gottschick C, Mikolajczyk R. Barriers to and facilitators of hepatitis B vaccination among the adult population in Indonesia: a mixed methods study. Vaccines. 2023;11(2):398.
56. Kim TV, Pham TND, Do B, Dao DVB, Nguyen DX, Lee W, et al. Low HBV knowledge is associated with low HBV vaccination uptake in general adult population despite incentivization of HBV vaccination. BMC Infect Dis. 2024;24(1):470.
57. Aber JO, Bongomin F, Opio SL, Ochola E. Hepatitis B vaccine uptake and associated factors among adults of Gulu city in Northern Uganda: A community-based cross-sectional study. Plos one. 2024;19(3):e0297617.
58. Lailatul Barik A, Purwaningtyas RA, Astuti D. The effectiveness of traditional media (leaflet and poster) to promote health in a community setting in the digital era: A systematic review. 2019.
59. Kollamparambil U, Oyenubi A, Nwosu C. Mental distress, COVID19 vaccine distrust and vaccine hesitancy in South Africa: A causal mediation regression analysis. PLOS ONE. 2023;18(3):e0278218.
60. Yörük S, Türkmen H, Durgut A, Erbek M. Vaccine mistrust among family healthcare professionals and vaccine hesitancy in the communities they serve in Turkey in 2019: a cross-sectional study. Human Vaccines & Immunotherapeutics. 2020;16(12):3155-62.
61. Feghaly E, Hanna V, Mohamad NK, Assaf MA, Sebastian J, El Khatib S, et al. Trust in pharmaceuticals and vaccine hesitancy: exploring factors influencing COVID-19 immunization among Lebanese children aged 1 to 11 years. BMC Pediatr. 2023;23(1):570.
62. Yu L, Wang J, Wangen KR, Chen R, Maitland E, Nicholas S. Factors associated with adults' perceived need to vaccinate against hepatitis B in rural China. Human Vaccines & Immunotherapeutics. 2016;12(5):1149-54.
63. Ebrahimi OV, Johnson MS, Ebling S, Amundsen OM, Halsøy Ø, Hoffart A, et al. Risk, Trust, and Flawed Assumptions: Vaccine Hesitancy During the COVID-19 Pandemic. Front Public Health. 2021;9:700213.
64. Huynh HP. Examining four types of anti-vaccination attitudes prior to and during the COVID-19 pandemic. Current Psychology. 2023;42(32):28812-9.
65. Ebrahimi OV, Sandbakken EM, Moss SM, Johnson SU, Hoffart A, Bauermeister S, et al. Modifiable risk factors of vaccine hesitancy: insights from a mixed methods multiple population study combining machine learning and thematic analysis during the COVID-19 pandemic. BMC medicine. 2025;23(1):155.

Figure

Click here to access/download;Figure;Fig-1.tiff

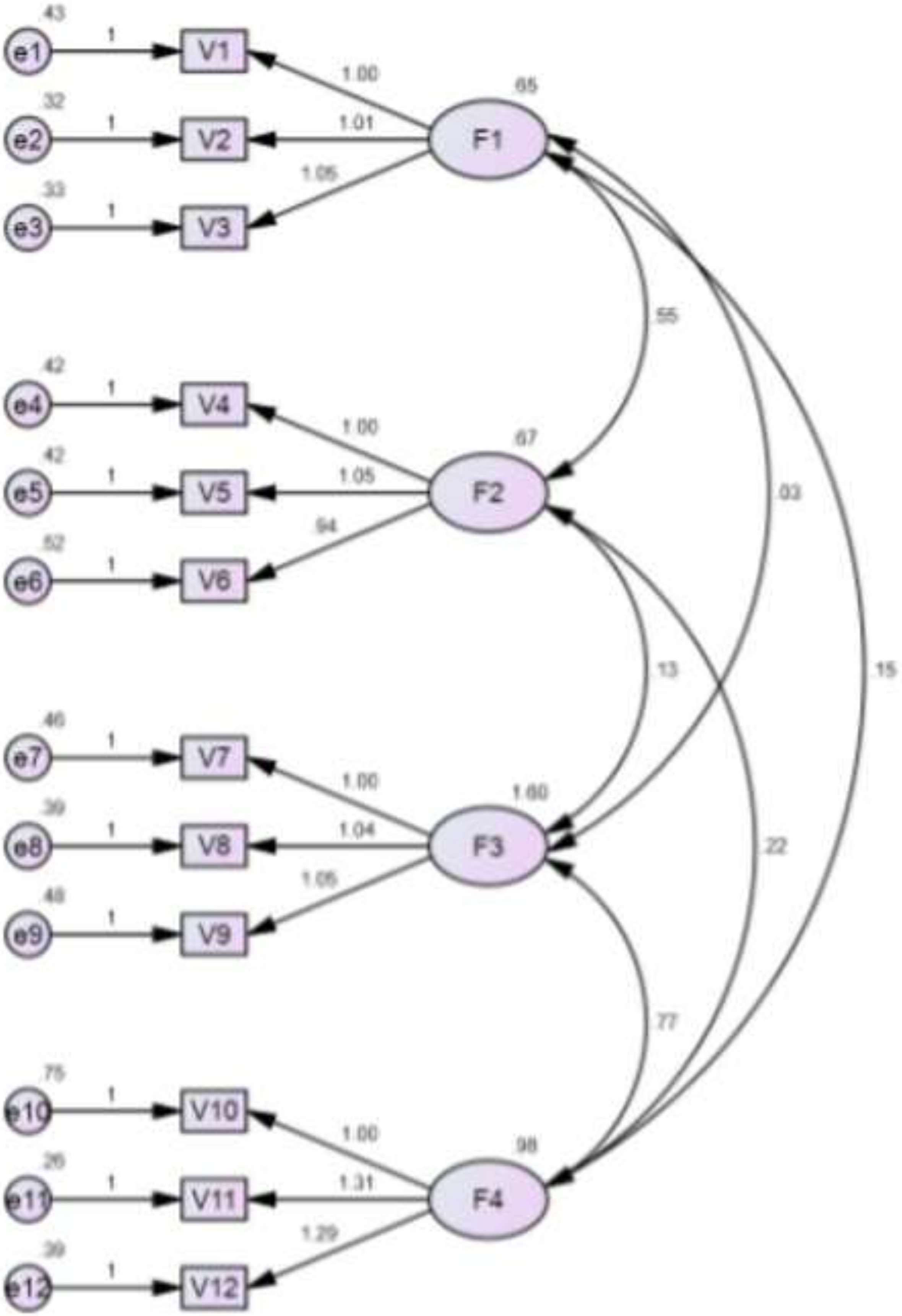

Figure

Click here to access/download;Figure;Fig-2.tiff

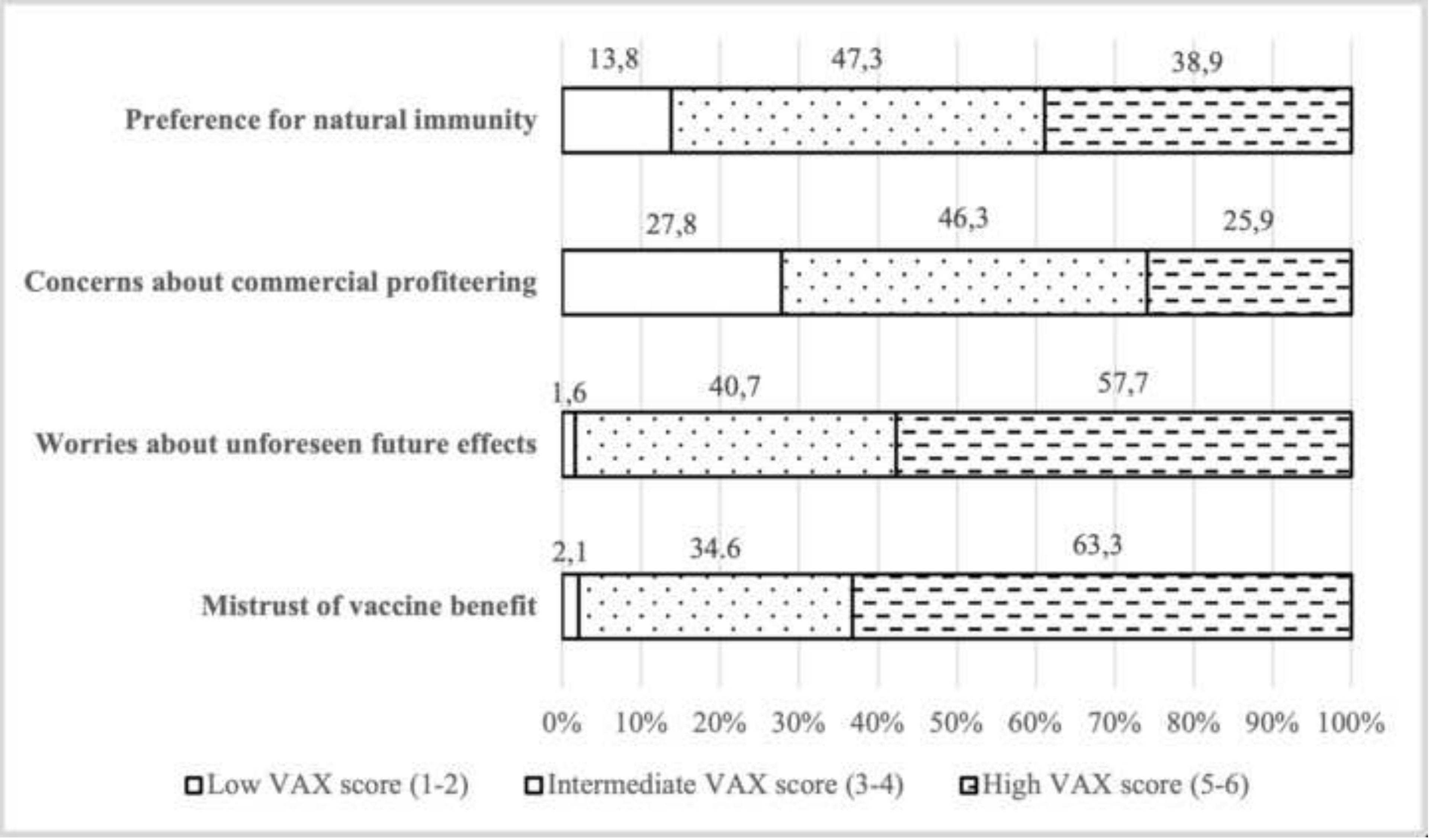

Click here to access/download;Figure;Fig-3.tiff

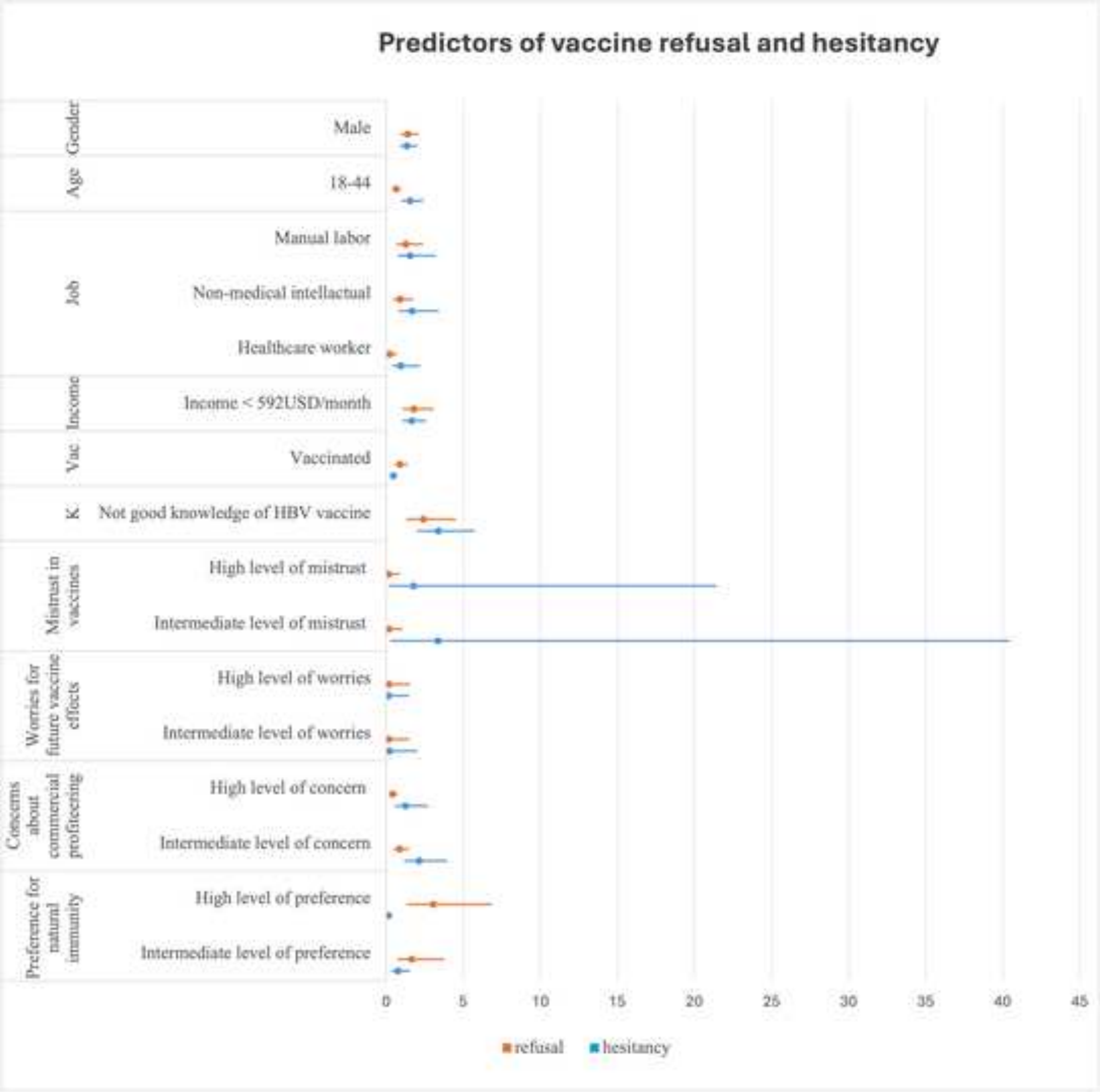

Click here to access/download
Supporting Information
Supporting information.docx

Click here to access/download
Supporting Information
Supporting file.1.docx

Willingness to accept the HBV vaccine and related factors: Administering the vaccination attitudes examination scale to Vietnamese adults

Nghi Ho Hoc[1], Tung Hoang[1], Cong Minh Le[2], Tram Thi Huyen Nguyen [1,*],

[1] Faculty of Pharmacy, University of Health Sciences, Vietnam National University Ho Chi Minh City, Ho Chi Minh city 700000, Vietnam.

[2] Center for Mental Health Research and Support, University of Social Sciences and Humanities, Vietnam National University Ho Chi Minh City, Ho Chi Minh City, 700000, Vietnam

***Corresponding author: Tram Thi Huyen Nguyen (PhD., BSc. Pharm.)**

[1] Faculty of Pharmacy, University of Health Sciences, Vietnam National University Ho Chi Minh City, Ho Chi Minh city 700000, Vietnam.

Add: YA1 Administrative Building, Hai Thuong Lan Ong Street, Dong Hoa Ward, Di An City, Binh Duong Province, Vietnam.

Email: nthtram@uhsvnu.edu.vn

Abstract

Vietnam is one of the countries in East Asian with a low hepatitis B vaccination coverage, posing a serious threat to the country's healthcare system. To mitigate the negative impacts of low herd immunity, it is essential to identify the population's level of vaccine hesitancy and refusal toward the HBV vaccine as well as the sociodemographic factors correlated with these behaviors. We conducted a cross-sectional survey of 926 adults in Vietnam from January to March 2025. When analyzing general vaccine intentions regardless of vaccination status, 65.6% of participants indicated acceptance, 19.3% hesitancy, and 15.1% refusal. A self-administered questionnaire and the vaccination attitudes examination (VAX) scale were used. Descriptive analysis was performed, and associations between variables were explored using a chi-square test. Multinominal logistical regression was conducted to identify the correlates of vaccine hesitancy and refusal. A $p < 0.05$ was considered indicative of statistical significance. The overall Cronbach's alpha was 0.963 (range = 0.812–0.919), and the McDonald's omega coefficients ranged from 0.820 to 0.920. The uniqueness values (0.115–0.422) indicated that substantial variances were explained by latent factors, with significant unstandardized loadings of 0.94 to 1.31 ($p < 0.001$). Knowledge about the HBV vaccine was positively correlated with mistrust of vaccine benefits ($r = 0.23$, $p < 0.01$), worries about future effects ($r = 0.19$, $p < 0.01$), preference for natural immunity ($r = 0.11$, $p < 0.01$), and total VAX score ($r = 0.19$, $p < 0.01$). Vaccine refusal was linked to age, occupation, income, knowledge, mistrust, commercial concerns, and preference for natural immunity, while hesitancy was associated with income, vaccination history, commercial concerns, and preference for natural immunity. The

VAX scale serves as a powerful and reliable instrument for assessing vaccine resistance and refusal in Vietnam. It not only helps identify key vaccine-related concerns but also supports continuous monitoring of public attitudes, which is essential for designing effective vaccination programs and enhancing public trust in vaccines.

Introduction

Hepatitis remains one of the most serious public health threats of the 21st century due to its severe long-term complications, such as liver cirrhosis, liver failure, liver cancer, and hepatocellular carcinoma, a problem that has prompted extensive global efforts to control and eliminate the disease [1]. Globally, approximately 254 million individuals were afflicted with hepatitis B virus (HBV), which was responsible for an estimated 1.1 million deaths, accounting for 83% of all viral hepatitis-related fatalities.The burden of chronic hepatitis B and C is highest among individuals aged 30 to 54 years, who represent half of all cases, while children under 18 years account for 12%. Men constitute 58% of the total infected population [1]. Moreover, even if the incidence of HBV infections has decreased globally, geographic disparities still exist. For example, the WHO stated that African and Southeast Asian regions are particularly affected, with these areas suffering from a 3% prevalence of the disease as well as bearing the burden of 60.5 million afflicted people and 180,000 HBV-related fatalities in 2019 [2].

For developing countries, conversely, achieving these goals remains particularly challenging. Vietnam, a middle-income nation in Southeast Asia with a population of 97 million, has an HBV prevalence of 7% to 8% [3], ranking 9th globally in terms of disease burden. In the country, as well, HBV infection is the leading cause of liver

cancer [4], with chronic hepatitis B accounting for up to 62.3% of cases at Cho Ray Hospital in Ho Chi Minh City [5].

Vaccination remains a key intervention in eliminating the HBV infection, consistent with the WHO's 2030 global elimination target. The hepatitis B vaccine is a safe and effective method of preventing HBV infection, with a birth dose and subsequent boosters providing nearly 100% protection [6]. Advancements in vaccine development also promise to curb HBV transmission by reducing mother-to-child transmission and enhancing vaccine immunogenicity in at-risk adult groups [7]. A one-year interruption in birth-dose and childhood HBV vaccination could cause 5.3 million additional chronic infections from 2020 to 2030, leading to 1 million more HBV-related deaths [6]. Vietnam has used HBV vaccination as part of its national immunization program since 2003, achieving the WHO target of 90% coverage for three doses and 50% coverage for birth doses by 2020 [8]. Nonetheless, vaccination rates among adults, those born before the implementation of the National Expanded Program on Immunization for children, remain low. This is largely due to Vietnam's National Action Plan for the Prevention and Control of Hepatitis B (2021–2025), which prioritizes children and high-risk groups rather than the broader adult population [9].

Meanwhile, vaccine hesitancy, defined by the SAGE Working Group as a delay or refusal to acquire vaccination despite the availability of services, has been listed by WHO as a top 10 global health threat since 2019 [10]. Such reluctance diminishes immunization rates, increasing the susceptibility of individuals and weakening herd immunity, which in turn, raise outbreak risks and may reverse progress against vaccine-preventable diseases [11]. Vaccine hesitancy has increased since the 2009 influenza

pandemic, as suggested in the literature [11]. However, the issue gained much greater attention and urgency during the COVID-19 pandemic, which caused widespread disruptions including prolonged lockdowns, increased misinformation, and significant interruptions to routine immunization services worldwide [12]. These factors have intensified the complexity of vaccine hesitancy, which is widely recognized as a complex and context-dependent issue, with attitudes ranging from full acceptance to outright rejection of vaccines. Notably, the reluctance to get vaccinated can occur even among individuals who acknowledge the importance of immunization [13]. This behavior is driven by several factors, including socioeconomic conditions, parental education, maternal healthcare access, and lack of knowledge. Broader influences encompass awareness, past experiences, perceived vaccination importance, social norms, religious or moral beliefs, distrust in vaccine manufacturers, and concerns regarding risk [13, 14]. Understanding the intricate interplay between the factors affecting vaccine acceptance is essential for public health practitioners, policymakers, and researchers to be able to optimize vaccination coverage and improve immunization strategies.

Such aims can be realized through the vaccination attitudes examination (VAX) scale, a robust instrument introduced by Martin and Petrie in 2017 to evaluate attitudes toward vaccination [15]. The scale has undergone thorough psychometric evaluation across various countries and has been translated into multiple languages, including Spanish, Romanian, Hebrew, Turkish, French, and Colombian [16-21]. Meanwhile, vaccine acceptance is largely influenced by the interplay between perceived disease risk and vaccine safety [22]. Individuals often prioritize avoiding disease exposure over

vaccination, particularly when personal risk is deemed low. Acceptance also varies across nations and cultural contexts, shaped by psychosocial factors and differing views on the advantages of vaccines [23]. These challenges highlight the importance of reliable psychometric tools, such as the VAX scale, in effectively assessing hesitancy-related factors. The VAX scale is designed to measure general attitudes towards vaccination rather than hesitancy related to a specific type of vaccine. This makes the VAX scale a versatile and valuable tool for assessing vaccine attitudes in diverse contexts, including during the COVID-19 pandemic.

On the basis of the studies discussed above, the current research was conducted to bridge the tremendous gap in our understanding of vaccination attitudes, specifically the degree of hesitancy and refusal toward the HBV vaccine and the independent factors influencing these behaviors among Vietnamese adults. By offering evidence-based insights to create focused interventions that successfully address vaccine hesitancy, the findings are anticipated to influence public health programming and initiatives. Furthermore, this study adds to the creation of a reliable and validated measure for assessing general vaccine attitudes, which may be used to assist continuous monitoring and targeted communication efforts to increase vaccination uptake in the Vietnamese population.

Materials and methods

Ethics statement

In this research, informed consent was secured from all participants, all of whom were aged 18 years and above. Before giving their agreement, participants were fully aware of the study's goals, methods, possible dangers, and benefits. Written consent was

acquired and appropriately recorded. The institutional ethics committee examined and approved the study protocol, which included the consent procedure.

Setting and research design

This study was conducted from January to March 2025. A cross-sectional design was implemented following the STROBE (Strengthening the Reporting of Observational Studies in Epidemiology) reporting guidelines.

Respondents were recruited through convenience sampling based on the following criteria: individuals aged 18 years or older, Vietnamese citizens, permanent residents of Ho Chi Minh City, able to understand the questionnaires, and willing to participate in the study. The selection process did not intentionally target any specific group, but individuals who were illiterate, were unable to understand Vietnamese, or provided incomplete survey responses were excluded from the analysis.

The required sample size was determined using the formula $\frac{Z^2_{\alpha-\alpha/2}p(1-p)}{d^2}$, where the standard normal variate ($Z_{1-\alpha/2}$) is 1.96 at a 95% confidence level (α = 0.05), the expected population proportion (p) is 0.5, and the margin of error (*d*) is 5% [4]. On the basis of this calculation, a minimum of 384 respondents was required to achieve adequate statistical power. To account for an anticipated 10% of incomplete responses, the target sample size was adjusted to 423, which was further expanded to 1,100 respondents to reduce sampling error.

Data collection and instruments

A structured self-administered questionnaire was developed on the basis of similar surveys and the relevant literature on the topic [15, 24, 25] before it was

modified to align with the study's objectives. The questions were refined to eliminate ambiguity, ensuring clarity and conciseness. The questionnaire demonstrated strong internal consistency, with a Cronbach's alpha (α) coefficient of 0.897. Following revisions, it exhibited good reliability and validity.

The final questionnaire consisted of three sections. The first encompassed five questions regarding sociodemographic characteristics, such age, gender, personal income, occupation, and vaccination status. The second section consisted of 10 questions about specific knowledge of HBV vaccines, and the final section comprised 12 questions covering vaccination intention and those in the VAX scale.

Data collection was conducted from January to March 2025 using either Google Forms or printed questionnaires. Our research collaborators approached individuals in public spaces, such as parks, cafés, and supermarkets, to distribute the questionnaire. Participation was voluntary, with informed consent forms acquired before proceeding with the survey. We maintained anonymity and confidentiality by refraining from collecting personally identifiable information. The respondents were told that they were free to withdraw at any time, but no incentives were offered to ensure an unbiased and diverse sample covering individuals of different socioeconomic backgrounds.

Questionnaire

Sociodemographic characteristics

The survey took on average of 20 minutes to complete. The following basic demographic details were derived: age (18–44 years, ≥45 years), gender (coded as 1 = male, 2 = female), personal income (in VND, with average monthly income categorized as follows: <VND 15 million (<US$ 605) and ≥VND 15 million (≥US$ 605),

occupation (with the following codes used: 1 = manual labor, 2 = nonmedical intellectual labor, 3 = healthcare worker, 4 = homemaker/retired/unemployed), vaccination status (coded as 1 = unvaccinated, 2 = vaccinated), and vaccine intention (coded as 1 = refused, 2 = hesitant, 3 = accepted).

Knowledge of the HBV vaccine

Ten items in the vaccine knowledge section were equally weighted and scored dichotomously. Each question had the response options "yes,' "no," and "do not know." The total knowledge score was calculated by summing the number of correct answers, with the maximum possible score being 10. The respondents scoring 5 or higher were classified as having good knowledge of the HBV vaccine, while those scoring below 5 were considered to have poor knowledge [26, 27].

Vaccination intention

A six-point Likert scale was used to assess the respondents' intention to receive the HBV vaccine. They were asked to answer the question "How do you rate your chances of getting the HBV vaccine?" The scale ranged from 1 to 6, with ratings of 1 to 2 indicating unwillingness or refusal, 3 to 4 reflecting hesitancy, and 5 to 6 representing a strong likelihood of vaccine acceptance.

Vaccination attitudes examination scale

The VAX scale [15] consists of 12 items intended to measure general attitudes toward vaccinations. It comprises four three-item subscales: (1) mistrust of vaccine benefits, (2) worries about unforeseen future effects, (3) concerns about commercial profiteering, and (4) preference for natural immunity. The respondents were asked to rate their agreement with each item using a six-point scale ranging from 1 (*totally*

disagree) to 6 (*totally agree*). The level of negative attitudes toward vaccination across all four VAX domains was into three groups: low (1–2), intermediate (3–4), and high (5–6). The final score was obtained by calculating the mean scores for each subscale as well as the mean total score. Low scores indicated positive vaccination views, whereas high scores represented negative perspectives [15].

Data processing and statistical analyses

Data processing

The data processing was grounded in a structured approach to ensure accuracy and reliability. The dataset was cleaned by removing incomplete responses and missing values. For surveys that were completed using printed questionnaires, data entry was conducted manually by trained research assistants, followed by double-checking and verification to minimize entry errors and ensure data quality. Outliers were then identified and excluded to prevent bias in the analysis by using a box plot and the Shapiro–Wilk test.

Statistical analyses

Data from the printed and online survey forms were exported to Microsoft Excel (version 2024) and analyzed using IBM Statistical Package for the Social Sciences (SPSS) for Windows (version 26.0), AMOS, and the psych package in R. Continuous variables were reported as means with standard deviations (*SD*s), while categorical variables were presented as frequencies and percentages. Chi-square (χ^2) tests were carried out to examine differences in vaccine uptake across categorical variables, with statistical significance set at $p < 0.05$. Additionally, multinominal analyses were performed to identify the independent factors associated with vaccine hesitancy and

refusal.

Confirmatory factor analysis (CFA) was performed using maximum likelihood parameter estimates with standard errors and mean-adjusted chi-square tests to validate the structural model. Model fit was evaluated on the basis of several indices with established cut-off criteria: chi-square test, comparative fit index (CFI), Tucker–Lewis index (TLI), root mean square error of approximation (RMSEA), and standardized root mean square residual (SRMR). The internal reliability of the VAX scale was assessed using Cronbach's α and McDonald's omega (ω) coefficients. Measurement invariance across genders (male vs. female) was tested via three nested models with increasing constraints: configural invariance, metric invariance, and scalar invariance. Measurement invariance was confirmed when the change in CFI (ΔCFI) remained below 0.01 [28].

We used multinomial logistic regression to estimate adjusted odds ratios (AOR) and 95% confidence intervals for hesitancy and refusal versus acceptance. Multinomial logistic regression was performed to identify the correlates of vaccine hesitancy and refusal. Vaccine acceptance was used as the reference outcome. The reference category for gender was female; for age, 45 years or older; for occupation, ohmemaker/retired/unemployed; for income, at least 605 USD per month; for vaccination status, not yet vaccinated; for HBV knowledge, good knowledge; and for VAX scores, the low VAX score group.The variables considered theoretically important, including demographic characteristics (e.g., age, sex, and socioeconomic status) and interpersonal factors (e.g., knowledge of hepatitis B vaccination), were entered into the model. Statistical significance was assessed using two-tailed tests, with

$p < 0.05$ considered indicative of a statistically significant association.

Ethical considerations

This study was approved by the Research Ethics Committee of the University of Health Sciences at Vietnam National University in Ho Chi Minh City (IRB No. 01/QD-HDDD). All procedures adhered to the ethical principles outlined in the Declaration of Helsinki. The authors affirm that all procedures in this study complied with the ethical standards of national and institutional committees on human research, as well as the 2008 version of the Helsinki Declaration. The survey was designed to maintain participant privacy, with no personally identifiable information collected. Participation was entirely voluntary and anonymous, with informed consent obtained and documented before the surveys were administered. All collected data were kept confidential and used exclusively for research purposes.

Risk of bias assessment

Quality assessment was performed using the STROBE checklist, which is designed to evaluate the quality and transparency of observational studies. The checklist includes key items related to study design, participants, variables, biases, statistical methods, results, and interpretations [29] (**Appendix**).

Results

Demographic characteristics of the participants

The sociodemographic characteristics of the participants are summarized in **Table 1**. There were a total of 926 participants, among whom 49.6% were male (*n* = 459), 35.3% (*n* = 327) were 45 years or older, and over half (64.7%, *n* = 599) were aged

18 to 44 years. more than 80% (*n* = 676) earned less than US$605 monthly. Occupationally, 42.3% (*n* = 392) were nonmedical intellectual laborers, 34.8% (*n* = 322) were manual laborers, 11.6% (*n* = 107) were healthcare workers, and 11.3% (*n* = 105) were homemakers/retired/unemployed. Among the participants, 41.4% (*n* = 383) were unvaccinated, whereas 58.6% (*n* = 543) had received the HBV vaccine. Regarding vaccination status, 58.6% (n = 543) of participants had been vaccinated, while 41.4% (n = 383) had not. When analyzing vaccination intentions, acceptance was 65.6%, hesitancy 19.3%, and refusal 15.1%.

Table 1. Sociodemographic characteristics of the study population (*N* = 926).

Variable	Number	Percentage (%)
**Gender**		
Male	459	49.6
Female	467	50.4
**Age (years)**		
18-44	599	64.7
≥ 45	327	35.3
**Occupation**		
Manual labor	322	34.8
Non-medical intellectual labor	392	42.3
Healthcare worker	107	11.6
Homemaker/Retired/Unemployed	105	11.3
**Income (US Dollar[1]/month)**		
< 605	676	73.0
≥ 605	250	27.0
**Vaccination status**		
Vaccinated	543	58.6
Not yet	383	41.4
**Vaccination intention among unvaccinated participants (n = 383)**		
Intent to vaccinate	275	71.8
No intention	108	28.2
**General vaccine intentions regardless vaccination status (n=926)**		
Accept	607	65.6
Refusal	140	15.1
Hesitant	179	19.3

[1]US$1 = VND24,786 (*source:* Vietnamese Ministry of Finance: exchange rate for foreign currencies in March 2025)

Table 2 presents respondents' knowledge about the Hepatitis B (HBV) vaccine, including its effectiveness, dosing, duration of protection, necessity of post-vaccination testing, and safety. The data indicate high awareness in certain areas, with 86.4% of participants correctly identifying that an effective vaccine exists (K1) and knowing where to get vaccinated (K10). Additionally, a majority (80.2%) recognized that the vaccine is safe (K9), and 75.2% understood that a complete dose is 95% effective (K3). However, while knowledge about HBV vaccine efficacy and accessibility is good, misconceptions about transmission risks and vaccine safety remain. Only 63.3% correctly answered that a fully recovered patient cannot infect others (K5), suggesting uncertainty about HBV transmission. Furthermore, 31.6% of respondents believed that the HBV vaccine may not cause harmful side effects (K8), highlighting safety concerns that could impact vaccine acceptance.

Table 2. Knowledge about HBV vaccine (n=926)

**Items**	**Varibles**	**Correct response, n (%)**	**Incorrect response, n (%)**
K1	There is effective vaccine for hepatitis B	800 (86.4)	126 (13.6)
K2	Hepatitis B vaccine dosing 0, 1, 6 months: 3 doses	679 (73.3)	247 (26.7)
K3	Complete dose of Hepatitis B vaccine is 95% effective	696 (75.2)	230 (24.8)
K4	It provides protection for at least 20 years	647 (69.9)	279 (30.1)
K5	A patient who has fully recovered cannot infect others	586 (63.3)	340 (36.7)
K6	Post-Hepatitis B vaccination test is necessary	680 (73.4)	246 (26.6)
K7	For protection against Hepatitis B, one needs antibody titre of >10 IU/m	652 (70.4)	274 (29.6)
K8	The HBV vaccine may cause harmful side effects for recipients	633 (68.4)	293 (31.6)
K9	The HBV vaccine is safe for recipients	743 (80.2)	183 (19.8)

K10	You know where to get the HBV vaccination	800 (86.4)	126 (13.6)

Table 3 presents the VAX scale data. As previously stated, the mean scores ranged from 3.14 (Item 9) to 4.49 (Item 1). The internal dependability of the majority of the items was indicated by the adjusted item-total correlations exceeding 0.40. Items V1, V2, and V3 contributed less to the scale, as their correlations fell below this threshold.

Table 3. Descriptive statistics of the VAX scale

Item	Min	Max	Mean	Std. Deviation	Skewness	Kurtosis	Item-total correlation
V1	1	6	4.49	1.04	-1.02	0.77	0.37
V2	1	6	4.43	0.99	-0.97	0.83	0.38
V3	1	6	4.39	1.02	-0.87	0.44	0.36
V4	1	6	4.35	1.04	-0.81	0.25	0.40
V5	1	6	4.33	1.07	-0.82	0.23	0.46
V6	1	6	4.29	1.05	-0.73	0.04	0.47
V7	1	6	3.29	1.43	-0.14	-1.10	0.53
V8	1	6	3.24	1.45	-0.11	-1.17	0.55
V9	1	6	3.14	1.50	-0.02	-1.27	0.50
V10	1	6	3.80	1.31	-0.58	-0.63	0.62
V11	1	6	3.67	1.38	-0.50	-0.87	0.63
V12	1	6	3.67	1.41	-0.52	-0.90	0.61

Preliminary analysis

Model fit was significant ($\chi^2(32) = 287.40$, $p < 0.001$), indicating that the final model provided a better fit than the intercept-only model. Goodness-of-fit tests showed an adequate fit ($\chi^2(904) = 818.02$, $p = 0.981$), although Pearson χ^2 was significant. Pseudo-R^2 values suggested moderate explanatory power (Cox & Snell = 0.267; Nagelkerke = 0.322; McFadden = 0.176). Likelihood ratio tests indicated that age, occupation, income, vaccination status, HBV knowledge, and three VAX subscales (F1, F3, F4) significantly contributed to the model (all $p < 0.05$). Multicollinearity was

examined using Variance Inflation Factors (VIF), all predictors showed VIF values ranging from 1.34 to 2.10, these results indicate that multicollinearity was not a concern in the regression model.

Parallel factor analysis confirmed the validity of the four-factor structure of the VAX scale, with a strong fit ($\chi^2(24, N = 926)$ at 41.61, a $p = 0.01$, a TLI = 0.99, and an RMSEA = 0.028 (95% confidence interval [CI]: 0.01–0.04). The model explained 70.04% of the cumulative variance in the data. A KMO = 0.85 indicated sampling adequacy, and Bartlett's test showed substantial item correlations ($\chi^2(66, N = 926) = 7050.23$, $p < .001$), permitting component analysis (see Table 4).

Table 4. Factor loadings, explained variances, and reliability.

**Items**	**F1**	**F2**	**F3**	**F4**	**Uniqueness (PAF)**
V1	-0.010				0.419
V2	-0.012				0.284
V3	0.034				0.315
V4		0.318			0.414
V5		0.090			0.345
V6		-0.106			0.419
V7			-0.053		0.209
V8			0.012		0.190
V9			0.073		0.207
V10				0.159	0.422
V11				-0.058	0.115
V12				-0.058	0.213
Cronbach's alpha (α)	0.848	0.812	0.919	0.890	
McDonald's omega (ω)	0.850	0.820	0.920	0.900	

F1 = mistrust of vaccine benefit; F2 = worries about unforeseen future effects; F3 = concerns about commercial profiteering; F4 = preference for natural immunity.

The CFA based on the maximum likelihood χ^2 S-B estimator for nonnormal data validated the VAX scale's four-component structure. The results suggested a strong fit—$\chi^2(49, N = 926) = 198.454$, $p < 0.001$, CFI = 0.98, TLI = 0.97, RMSEA = 0.06 (90% CI: 0.05–0.07); internal consistency was satisfactory, with Cronbach's α ranging from 0.812 to 0.919 and McDonald's ω ranging from 0.820 to 0.920 across subscales,

demonstrating satisfactory internal consistency. Most of the items significantly contributed to latent components, but those with high uniqueness values (>0.40) pointed to weaker correlations or ambiguity.

The CFA validation of the VAX scale structure also verified the scale's constructs (see Fig 1).

Fig 1. CFA results: Path diagram and standardized estimated parameters for the Vietnamese VAX scale.

The interfactor correlations were inconsistent, with the strongest correlation between F1 and F4 (r = 0.77) and the weakest between F1 and F2 (r = 0.03), indicating a variety of attitudinal dimensions on vaccine attitudes. Measurement invariance across genders showed no differences, implying that the male and female participants understood the VAX scale in the same manner (see Supplementary Table 1). Strong associations, such as those between F1–F2 (r = 0.61, p = 0.01) and VAX_Total-F4(r = 0.782, p = 0.01), indicated F4's substantial contribution to unfavorable vaccination beliefs, as evidenced by the Pearson correlations between the subscales and VAX_Total.

Age was associated with favorable vaccination views, reflecting decreased mistrust (r = –0.10, p < 0.01), worries about future effects (r = –0.09, p = 0.05), profiteering concern (r = –0.07, $p < 0.01$) and concern about commercial profiteering (r = –0.07, p < 0.05). Employed individuals exhibited increased vaccine hesitancy compared with unemployed/retired individuals, even under negative associations across categories (e.g., mistrust r = –0.11, p = 0.01). Getting vaccinated was negatively associated with VAX_Total (r = –0.15, p = 0.01) and particular worries (e.g., profiteering: r = –0.14, p = 0.01), reflecting favorable attitudes among those receiving

the vaccine. The intention to receive vaccination was substantially linked to income ($r = 0.12$, $p < 0.01$) and VAX_Total ($r = 0.14$, $p < 0.01$), albeit with respondents exhibiting mild suspicion and fears (e.g., F1: $r = 0.18$, $p = 0.01$). Knowledge of HBV vaccination was positively correlated with F1 ($r = 0.23$, $p = 0.01$), F2 ($r = 0.19$, $p = 0.01$), F4 ($r = 0.11$, $p = 0.01$), and VAX_Total ($r = 0.19$, $p = 0.01$), denoting that strong knowledge is related to increased fears and cautious attitudes toward vaccination. These results show the complex interaction between demographic attributes, knowledge of vaccines, and vaccination attitudes in the Vietnamese context (see Fig 2).

Fig 2. Perceptions of vaccination.

Among the respondents highly suspicious of vaccine benefits (high group), 63.3% expressed concerns about vaccine efficacy compared with those exhibiting low suspicion (low group, 2.1%). Concerns regarding unexpected consequences were more frequent in the high group (57.7%) than in the low group (1.6%). Commercial profiteering concerns were fairly distributed across the sample, with 46.3% of participants in the intermediate group, 27.8%, and 25.9% exhibiting moderately strong, low, and strong concerns, respectively. Ultimately, the moderate group (47.3%) favored natural immunity the most, followed by the high (38.9%) and low (13.8%) groups. Of the participants, 65.6% were ready to receive vaccination, 19.3% remained dubious, and 15.1% were reluctant.

Comparison of intention on the basis of VAX subscale mean scores

A one-way ANOVA was conducted to compare the mean scores on the VAX subscales of the refusal, hesitant, and willing groups for HBV vaccination. With modest effect sizes, the willing group scored far higher on F1 (mistrust of vaccine advantages) and F2 (worries about future consequences) than the hesitant or refusal groups ($p <$

0.001). With a $p < 0.001$, the refusal group scored higher on F4 (preference for natural immunity). The groups' F3 (commercial profiteering concerns) scores had no significant differences (see Supplementary Table 2).

Predictors of HBV vaccine hesitancy and refusal

The results indicate that individuals with a high level of mistrust were 85.3% less likely to refuse the vaccine but 78.8% more likely to exhibit hesitancy compared to those with low level of mistrust. Similarly, those with a high level of worries were 82.2% less likely to refuse the vaccine and 85.0% less likely to be hesitant than individuals with low levels of worries. Among participants with a high level of concern about commercial profiteering, vaccine refusal decreased by 59.3%, while hesitancy increased by 41.6% compared to those with low level of concern. In contrast, individuals with a high preference for natural immunity were more than twice as likely to refuse the vaccine and 84.4% less likely to be hesitant compared to those with a low preference (see Fig 3).

Fig 3. Predictors of HBV vaccine hesitancy and refusal using multinominal logistic regression analysis.

Predictors of refusal to vaccinate against HBV

We found that the refusal to receive the HBV vaccine was higher among the participants with personal incomes of less than US$605 per month (AOR = 1.82, 95% CI: 1.08–3.07) and those with poor knowledge of the HBV vaccine (AOR = 2.42, 95% CI: 1.30–4.53). Such refusal was lower among the healthcare workers (AOR = 0.23, 95% CI: 0.07–0.72) and participants aged 18 to 44 years (AOR = 0.64, 95% CI: 0.41–0.99). The refusal to get vaccinated was also greater among the participants who strongly favored natural immunity (AOR = 3.06, 95% CI: 1.37–6.84). It was lower

among the respondents who were substantially concerned about commercial profiteering (AOR = 0.41, 95% CI: 0.23–0.73) but lower among those who mistrusted vaccine benefits (AOR = 0.15, 95% CI: 0.03–0.86).

Predictors of hesitancy to vaccinate against HBV

We identified the predictors of hesitancy and acceptance of the HBV vaccine. As mentioned earlier, hesitancy was observed in the participants with personal incomes of less than US$605 dollars (AOR = 1.66, 95% CI: 1.06–2.60) and those with poor knowledge of the HBV vaccine (AOR = 3.39, 95% CI: 2.01–5.71). Such reluctance was greater among the respondents displaying moderate concerns about commercial profiteering (AOR = 2.14, 95% CI: 1.16–3.95). In contrast, hesitancy was lower among the participants highly preferring natural immunity (AOR = 0.16, 95% CI: 0.07–0.37) (**Fig 3**).

Discussion

Evidence points to although most people are aware of the HBV vaccination's efficacy and accessibility, there are still considerable misconceptions about transmission hazards and vaccine safety. This knowledge gap could lead to increased anxiety as individuals become aware more about the vaccine but are still unsure about possible adverse effects or misjudge how HBV spreads. These misunderstandings may impact their choice for different ways to protect themselves from HBV infection. Furthermore, there are some pieces of information that deal with technical aspects and side effects of vaccines (such as antibody titers and post-vaccination testing), which can be complicated and hard to grasp, leading people to misunderstand or be confused. It is possible that individuals who have a deeper acquaintance with these features will

develop a stronger awareness of rare adverse effects and the complexity involved in vaccine-induced immunity, which may lead to increased concerns about unanticipated future implications. The current pattern indicates a measured reluctance to trust without first gathering relevant information.

The study demonstrates that the mean VAX scores were 3.0 or higher, suggesting moderate to high skepticism toward vaccination. However, vaccine acceptance remained reasonably high, at 65.6%. This discrepancy implies that vaccination acceptance is not directly predicted by attitudes as assessed by the VAX scale. This could be explained by a number of factors, including perceived dangers of not getting vaccinated, the immediacy of the health threat, cultural and social influences, and the accessibility and affordability of vaccines. For instance, many people decided for vaccination during the COVID-19 pandemic in spite of concerns over its efficacy or safety. It is crucial to remember that the VAX scale evaluates skepticism or trust in vaccines rather than acceptance of them. Consequently, vaccination adoption is impacted by a complex interaction of factors beyond attitude alone, even when VAX scores indicate widespread hesitation.

Our findings support the factorial structure of the VAX scale: mistrust of vaccine benefits (F1), worries about unforeseen future effects (F2), concerns about commercial profiteering (F3), and preference for natural immunity (F4). This four-factor solution aligns with the original VAX structure proposed by Martin and Petrie [15] and is consistent with findings from other cross-cultural validations, such as those conducted in Romania [17], Italy [25], and Turkey [19]. The results also support the reliability and validity of the VAX scale in the Vietnamese context, a culturally distinct setting,

suggesting that the scale maintains its psychometric robustness across diverse cultural backgrounds.

Vaccine hesitancy remains one of the major barriers to the elimination of hepatitis B. Numerous studies have assessed the acceptance of the HBV vaccine and have discovered a reluctance to receive vaccination, even in countries with high immunization rates. Common concerns include potential side effects (e.g., multiple sclerosis) [30-32], a perceived low risk of infection in newborns, and a greater fear of vaccine-related harm, such as infant death, than of the disease itself[33-35]. Other contributing factors are the lack of strong recommendations from healthcare providers [36-38], limited media communication [39, 40], low education levels, insufficient knowledge about HBV infection risks [41], and a general perception that the vaccine is unnecessary [42], among other issues.

In an Italian sample, high levels of vaccine hesitancy were observed among healthcare workers with low educational attainment, with such hesitancy frequently associated with fears of developing multiple sclerosis, as well as beliefs linking aluminum-based adjuvants in vaccines to Alzheimer's disease [43]. Racial or ethnic disparities in vaccine acceptance have also been reported. Vulnerable populations, such as pregnant women [44], hemodialysis patients [45], people living with HIV [46], and men who have sex with men, are particularly affected by these attitudes [47].

The configuration, metric, and scalar levels of measurement invariance were all validated in the examination of the factorial structure during the gender-based multigroup analyses. The consistent underlying structure, item loadings, and intercepts across gender groups of the VAX scale validated its effectiveness in evaluating vaccine

attitudes equivalently for men and women. Consistent with results of Bruno et al., our findings confirmed the suitability of the scale for gender-based comparisons [25]. Such uniformity and cross-group comparability offer strong support for further studies on gender variations in self-reported vaccine reluctance with conceptual consistency.

Our study found a 65.6% acceptance rate of hepatitis B vaccination, which is lower than that found in the Indonesian context (84.5%), where many participants were healthcare professionals with better awareness of health and access to insurance coverage of vaccination expenses [48]. On the other hand, our rate exceeds that derived in a Chinese study, in which only 20.67% of unvaccinated individuals regarded the hepatitis B vaccine necessary, mostly due to safety and efficacy matters [49]. These cross-country variations underscore the influence of sociocultural, informational, and systemic factors—such as health knowledge, access to vaccination services, and trust in healthcare institutions—that shape vaccine acceptance. Understanding these differences is critical for developing tailored public health interventions. Our findings contribute to the literature by highlighting how contextual factors impact vaccine tolerance across diverse populations.

With a projected population of 8.9 million by mid-2023, Ho Chi Minh City, Vietnam's most populous and economically vibrant city, offers a unique socioeconomic environment that might help explain its high hepatitis B vaccination rates [50]. As a major financial, commercial, educational, and technological hub, the city has undergone rapid urbanization and significant healthcare improvements through substantial investments in infrastructure and services. These developments have enhanced public access to healthcare information and services, increasing awareness of the importance

of vaccination. Additionally, coordinated efforts among local authorities, NGOs, and healthcare providers have been pivotal in building public trust and encouraging participation in vaccination campaigns.

Despite these favorable conditions and ongoing public health initiatives, our findings reveal that the hepatitis B vaccination acceptance rate is lower than might be expected for a city with such advancements. This discrepancy suggests the presence of persistent barriers, such as vaccine hesitancy, misinformation, or unequal access among certain population groups. These factors highlight the complexity of vaccine acceptance and indicate that socioeconomic progress and campaign efforts alone may not be sufficient to achieve optimal vaccination coverage. Therefore, a deeper understanding of these contextual challenges is necessary to tailor more effective interventions and policies to increase vaccine uptake in Ho Chi Minh City.

Relationship between demographic characteristics and vaccine hesitancy and refusal

Contrary to earlier studies showing gender differences in vaccine uptake, gender was not a significant predictor of hepatitis B vaccine refusal in the present research. For example, a study among Laotian Americans found that women are far more likely to receive vaccination (adjusted OR = 4.0, 95% CI: 1.2–19), whereas men tend to believe in the preventability and treatability of HBV [51]. Similarly, studies on healthcare workers in five developing nations found that female workers are predisposed to complete the hepatitis B vaccine series, albeit the findings varied by setting [52]. Gender variations were also discovered in relation to other vaccines, with women found to be more sensitive to side effects or more strongly affected by issues such as pregnancy

[53]. These contradictions imply that sociocultural standards, healthcare availability, and vocational positions influence the effects of gender on vaccination acceptance. The absence of a gender effect in our study may reflect specific sociocultural factors in Vietnam. Traditional gender roles, coupled with relatively equal healthcare access and vocational opportunities in urban settings, might reduce gender disparities in vaccination acceptance. This contrasts with findings from other contexts, highlighting the importance of localized sociocultural and structural influences.

In this study, income level was a major determinant of the reluctance or refusal to get vaccinated. Low earners were around 1.8 times more likely than high-income individuals to refuse or hesitate with respect to vaccination. This corresponds with prior research on social inequalities in immunization rates, such as studies in rural China highlighting poverty as a primary factor in hepatitis B vaccination disparities [54]. In Vietnam, economic hardship intersects with limited access to trustworthy health information and lower institutional trust, which may intensify vaccine hesitancy among low-income groups. Moreover, unlike some low-income countries where increased government vaccine delivery effectively boosts coverage, structural and sociocultural barriers in Vietnam require tailored public health strategies. These contextual and population-specific factors not only reinforce the global pattern linking low income to vaccine hesitancy but also emphasize the need for interventions addressing both financial and informational obstacles to improve vaccination uptake in Vietnam.

In interpreting the findings on vaccine hesitancy and refusal, it is important to clarify how the VAX variable captures attitudes toward hepatitis B vaccination. The VAX measure reflects participants' general views about vaccination, regardless of

whether they have previously been vaccinated. Therefore, even individuals who have already received one or more doses may still report “accept,” “hesitant,” or “refusal” depending on their current attitude. Based on this framework, the refusal group likely includes those who do not intend to vaccinate (108 participants) as well as some previously vaccinated individuals who could refuse additional doses or oppose recommending vaccination. The hesitant group may include individuals who have initiated vaccination but remain uncertain about completing the series. Meanwhile, the accept group consists of those who intend to vaccinate (275 participants) and those who are already vaccinated. Distinguishing between actual vaccination status and future vaccination intention helps resolve the seeming inconsistency in the results and provides a clearer understanding of how attitudes toward vaccination vary across demographic groups.

Individuals with inadequate knowledge regarding HBV vaccination exhibit vaccine refusal or hesitancy that is two to three times greater than that among those with a sufficient understanding of the issue. Research conducted in Indonesia uncovered that a core obstacle to immunization is ignorance of its advantages [55]. Similar trends were noted in Vietnam and Uganda, where little knowledge of HBV and its transmission diminishes the acceptability of vaccination [56, 57]. Efforts should thus prioritize low-awareness communities, with easily available, culturally acceptable resources used as part of campaigns. Particularly in rural or underprivileged regions, tools such as illustrated fliers, pamphlets, and short educational movies with clear graphics should be extensively available in local health clinics, schools, marketplaces, and mobile health units [58]. Involving local healthcare professionals, leaders, and influencers can also

help build confidence and increase community participation. These strategies can increase health literacy, thereby reducing hesitation and encouraging the equitable distribution of HBV vaccines.

In this study, psychological and attitudinal factors clearly affected HBV vaccination reluctance and rejection. First, individuals with a high level of mistrust in vaccine benefits were significantly less likely to refuse the vaccine (by 84.3%) but more likely to exhibit hesitancy (by 78.8%) compared to those with lower levels of mistrust. This suggests that mistrust may not necessarily lead to outright rejection but rather to uncertainty and cautious indecision. In other words, mistrustful individuals may delay or question vaccination without completely refusing it, Kollamparambil et al. (2023), for example, demonstrated that vaccine distrust was the strongest predictor of vaccine hesitancy in South Africa, highlighting that individuals with high levels of distrust are more likely to vacillate or postpone vaccination than to reject it completely [59].

In the context of Vietnam, where hepatitis B infection remains highly prevalent, vaccination is recognized as an essential preventive measure. Therefore, even though some individuals express greater worries, they appear less likely to refuse or hesitate about receiving the vaccine. This may reflect a pragmatic understanding that the benefits of vaccination outweigh potential risks, particularly given the serious consequences of HBV infection. In other words, despite existing worries, the perceived necessity and urgency of protection against HBV encourage people to proceed with vaccination rather than avoid it.

Regarding concerns about commercial profiteering, participants who believed that vaccine promotion was mainly motivated by business or profit were less likely to

completely refuse the vaccine but more likely to hesitate. Their hesitation may come from a lack of trust rather than opposition to the vaccine itself. In practice, this indicates that improving transparency in vaccine distribution, providing clear information about pricing, and emphasizing public health benefits instead of commercial aspects could help reduce such doubts and encourage higher vaccine acceptance.

In contrast, individuals with a strong preference for natural immunity were more than twice as likely to refuse vaccination and 84.4% less likely to be hesitant. This suggests that those who believe natural infection provides stronger or more "authentic" protection than vaccines tend to make firm decisions against vaccination rather than remain uncertain. In contexts like Vietnam, where public awareness of HBV risk is increasing, such attitudes may pose a challenge for vaccine promotion because they represent a deep-seated worldview rather than a simple lack of information. Therefore, public health communication should not only address misinformation but also emphasize how vaccination safely strengthens, rather than replaces, natural immunity.

Underlining the intricacy of hesitation, a Turkish study among healthcare professionals identified mistrust, fears of injury or autism, media disinformation, and religious concerns as grounds for rejection [60]. In the present work, the participants displaying considerable concerns about vaccines were 60% less likely to decline vaccination, while those with moderate anxiety about this issue were 1.5 times more likely to hesitate[61]. Similar patterns were observed among individuals expressing concerns about commercial profiteering. Furthermore, our findings suggest that individuals with strong opinions tend to make more definitive decisions regarding vaccination, whether acceptance or refusal, compared to those with less pronounced

views [62]. This highlights that vaccine decision-making is influenced not only by the presence of concerns but also by the clarity and strength of personal attitudes.

The respondents who preferred natural immunity were three times more likely to reject HBV vaccination but were 85% less likely to display hesitation [63-65]. Consistent with the results derived in the context of France [31], our findings demonstrated that concerns about unanticipated future impacts had no appreciable influence on rejection or hesitation. These outcomes emphasize the importance of addressing the psychological as well as socioeconomic factors of vaccination behavior. Public health messages should be customized to address issues such as commercial uncertainty or natural immunity preferences to build trust by means of evidence-based, sympathetic communication.

Vaccine refusal and hesitation ultimately result from a confluence of psychological and sociodemographic elements. The results can inform future studies and enable public health experts to create sensible plans. Important activities include addressing personal issues, offering correct information on vaccine safety and efficacy, fighting false information, and involving medical professionals in education campaigns. Vaccine acceptance and confidence in healthcare systems can be improved even more by customizing communication to match community-specific issues of age, income, and education levels.

Strengths and limitations of the study

This study presents several strengths. Notably, the VAX scale's reliability and validity in the Vietnamese context are guaranteed by the fact that it is the first to do a psychometric validation of the scale among Vietnamese adults, confirming its original

four-factor structure and proving measurement invariance across genders. The study also finds actionable correlations of vaccine reluctance and refusal, such as income, hepatitis B vaccination knowledge, natural immunity beliefs, and commercial interest concerns. Last but not least, these results establish precise programmatic goals to raise adult hepatitis B vaccination rates, giving legislators important direction for creating efficient vaccination laws, improving program execution, and customizing public health initiatives. Limitations include the results' applicability to the larger Vietnamese population, particularly rural populations, may be limited by the use of convenience sampling that was concentrated on metropolitan regions. Using self-reported data could lead to social desirability bias or reporting bias. Furthermore, no causal conclusions can be made about the associations found because of the cross-sectional study methodology. Lastly, residual confounding from unmeasured factors cannot be completely ruled out even after controlling for a number of covariates.Future research should adopt longitudinal designs, diversify data collection methods, and randomize item order to reduce consistency bias from consecutive item presentation.

Conclusion

Despite favorable conditions and ongoing public health efforts in Ho Chi Minh City, the acceptance of the hepatitis B vaccine remains lower than expected, indicating the presence of persistent barriers such as vaccine hesitancy, misinformation, and unequal access. Our research emphasizes that vaccine decision-making is influenced by the strength and lucidity of personal attitudes toward vaccination, in addition to existing concerns. The VAX scale has been demonstrated to be a valuable instrument for accurately evaluating these attitudes within the Vietnamese population in this context.

By identifying specific factors contributing to hesitancy through this scale, public health interventions can be better tailored to address these challenges and ultimately improve vaccination rates.

Abbreviation

HBV*:* Hepatitis B Virus**, CDC*:*** Centers for Disease Control and Prevention**, CFA**: Confirmatory factor analysis**, DALYs:** disability-adjusted life years, **HCV:** Hepatitis C Virus, , ***HCMC:*** Ho Chi Minh City**, NGOs**: non-governmental organizations, ***OR:*** Odds Ratio, **KAP*:*** Knowledge Attitude and Practice**, SES*:*** Socioeconomic status, **VAX:** Vaccination Attitudes Examination, **WHO*:*** World Health Organization.

Supplementary materials

Supplement Table 1. Test of the invariance of VAX scale across gender
Supplement Table 2. One-way Anova results of the difference among the total score of the VAX and intention to get HBV vaccine.
Supplement Table 3. Pearson's correlation, means, standard deviation (SD) for VAX subscales, and total score

Author contributions

T.N. contributed to conception the research idea and creating the research design T.H and N.H contributed to the acquisition of data and analysis. C.L. and T.N. contributed to drafting the manuscript's intellectual content. All authors had full access to all the data in the study and take the responsibility for the integrity of the data and the accuracy of the data analysis. All the authors have read and agree to the published version of the manuscript.

Financial Disclosure

This research is funded by Vietnam National University Ho Chi Minh City (VNU-HCM) under grant number 36-2025-44-02. The funders had no role in study design, data collection and analysis, decision to publish, or preparation of the manuscript.

Institutional Review Board Statement: This study was conducted in accordance with the Declaration of Helsinki and approved by the Institutional Ethics Committee of University of Health Sciences, Vietnam National University (Ethics No. IRB No. 01/QD-HDDD). No Animals were used in the studies that were the basis of this research. All human procedures were carried out in accordance with the ethical standards of the committee responsible for human experimentation (institutional and national) and with the Helsinki Declaration of 1975, as revised in 2013.

Informed Consent Statement: Informed consent was obtained from all subjects involved in the study.

Data availability Statement: All relevant data are within the paper and its Supporting Information files.

Acknowledgments

We thank the respondents from pharmacists who gave us valuable inputs in the questionnaire development phase that helped us to improve the questionnaire and adapt it for the local population.

Conflict of Interest: The authors declare no conflict of interest.

APPENDIX

Appendix 1. STROBE Statement- Checklist of items that should be included in reports of *cross-sectional studies*

REFERENCES

1. Organization WH. Hepatitis B 2024 [cited 2024 Oct 2]. Available from: https://www.who.int/news-room/fact-sheets/detail/hepatitis-b.
2. Devarbhavi H, Asrani SK, Arab JP, Nartey YA, Pose E, Kamath PS. Global burden of liver disease: 2023 update. Journal of hepatology. 2023;79(2):516-37.
3. Pham TND, Le DH, Dao DVB, Phan LTB, Pham TTT, Nguyen TB, et al. Establishing baseline framework for hepatitis B virus micro-elimination in Ho Chi Minh City, Vietnam - A community-based seroprevalence study. Lancet Reg Health West Pac. 2023;30:100620.
4. Global prevalence, treatment, and prevention of hepatitis B virus infection in 2016: a modelling study. Lancet Gastroenterol Hepatol. 2018;3(6):383-403.
5. Nguyen-Dinh SH, Do A, Pham TND, Dao DY, Nguy TN, Chen MS, Jr. High burden of hepatocellular carcinoma and viral hepatitis in Southern and Central Vietnam: Experience of a large tertiary referral center, 2010 to 2016. World J Hepatol. 2018;10(1):116-23.
6. de Villiers MJ, Nayagam S, Hallett TB. The impact of the timely birth dose vaccine on the global elimination of hepatitis B. Nature communications. 2021;12(1):6223.
7. El-Sayed MH, Feld JJ. Vaccination at the forefront of the fight against hepatitis B and C. Nature Reviews Gastroenterology & Hepatology. 2022;19(2):87-8.
8. Komada K, Ichimura Y, Shimada M, Funato M, Do HT, Le HX, et al. Impact of hepatitis B vaccination programs in Vietnam evaluated by estimating HBsAg prevalence. Journal of Virus Eradication. 2022;8(4):100309.
9. Health VMo. Issuance of the national action plan for viral hepatitis prevention and management 2021-2025. 4531/QD-BYT. 2021.
10. Organization WH. Ten threats to global health in 2019 2019 [Available from: https://www.who.int/news-room/spotlight/ten-threats-to-global-health-in-2019?utm_source=chatgpt.com.
11. Larson HJ, Jarrett C, Eckersberger E, Smith DM, Paterson P. Understanding vaccine hesitancy around vaccines and vaccination from a global perspective: a systematic review of published literature, 2007–2012. Vaccine. 2014;32(19):2150-9.
12. Causey K, Fullman N, Sorensen RJD, Galles NC, Zheng P, Aravkin A, et al. Estimating global and regional disruptions to routine childhood vaccine coverage during the COVID-19 pandemic in 2020: a modelling study. Lancet. 2021;398(10299):522-34.
13. Jiang H, Wei C. How to address vaccine hesitancy? Lessons from national hepatitis B immunization program in China. Frontiers in Public Health. 2024;12:1286801.
14. Yaqub O, Castle-Clarke S, Sevdalis N, Chataway J. Attitudes to vaccination: a critical review. Soc Sci Med. 2014;112:1-11.

15. Martin LR, Petrie KJ. Understanding the dimensions of anti-vaccination attitudes: The vaccination attitudes examination (VAX) scale. Annals of Behavioral Medicine. 2017;51(5):652-60.
16. Paredes B, Cárdaba MÁ, Cuesta U, Martinez L. Validity of the Spanish version of the vaccination attitudes examination scale. Vaccines. 2021;9(11):1237.
17. Huza G. The psychometric properties of a Romanian version of the vaccination attitudes examination (VAX) scale. International Journal of HIV/AIDS Prevention, Education and Behavioural Science. 2020;6(1):25.
18. Shacham M, Greenblatt-Kimron L, Hamama-Raz Y, Martin LR, Peleg O, Ben-Ezra M, Mijiritsky E. Increased COVID-19 Vaccination Hesitancy and Health Awareness amid COVID-19 Vaccinations Programs in Israel. Int J Environ Res Public Health. 2021;18(7).
19. Yildiz E, Gungormus Z, Dayapoglu N. Assessment of validity and reliability of the turkish version of the vaccination attitudes examination (VAX) scale. Int J Caring Sci. 2021;14(1):261-9.
20. Eisenblaetter M, Madiouni C, Laraki Y, Capdevielle D, Raffard S. Adaptation and Validation of a French Version of the Vaccination Attitudes Examination (VAX) Scale. Vaccines (Basel). 2023;11(5).
21. Espejo B, Martín-Carbonell M, Romero-Acosta KC, Fernández-Daza M, Paternina Y. Evidence of Validity and Measurement Invariance by Gender of the Vaccination Attitudes Examination (VAX) Scale in Colombian University Students. J Clin Med. 2022;11(16).
22. Kim J, Han K, Chung SJ, Kim C. Psychometric validation of the Korean versions of the Vaccine Hesitancy Scale and Vaccination Attitudes Examination Scale. Vaccine. 2023;41(32):4685-92.
23. Cheng L, Kong J, Xie X, Zhang F. A psychometric assessment of a novel scale for evaluating vaccination attitudes amidst a major public health crisis. Scientific Reports. 2024;14(1):10250.
24. Biella M, Orrù G, Ciacchini R, Conversano C, Marazziti D, Gemignani A. Anti-Vaccination Attitude and Vaccination Intentions Against Covid-19: A Retrospective Cross-Sectional Study Investigating the Role of Media Consumption. Clin Neuropsychiatry. 2023;20(4):252-63.
25. Bruno F, Laganà V, Pistininzi R, Tarantino F, Martin L, Servidio R. Validation and psychometric properties of the Italian Vaccination Attitudes Examination (VAX-I) scale. Curr Psychol. 2022:1-11.
26. Shrestha DB, Khadka M, Khadka M, Subedi P, Pokharel S, Thapa BB. Hepatitis B vaccination status and knowledge, attitude, and practice regarding Hepatitis B among preclinical medical students of a medical college in Nepal. PloS one. 2020;15(11):e0242658.
27. Bhattarai S, Gyawali M, Sapkota S, Karki D, Lamsal S. Knowledge, attitude and practice of hepatitis B vaccination among clinical medical students at a medical college in Nepal. Europasian Journal of Medical Sciences. 2020;2(2):17-23.
28. Cheung GW, Rensvold RB. Evaluating goodness-of-fit indexes for testing measurement invariance. Structural equation modeling. 2002;9(2):233-55.
29. Von Elm E, Altman DG, Egger M, Pocock SJ, Gøtzsche PC, Vandenbroucke JP. The Strengthening the Reporting of Observational Studies in Epidemiology (STROBE) statement: guidelines for reporting observational studies. The lancet. 2007;370(9596):1453-7.
30. Gobert C, Semaille P, Van der Schueren T, Verger P, Dauby N. Prevalence and determinants of vaccine hesitancy and vaccines recommendation discrepancies among general practitioners in French-speaking parts of Belgium. Vaccines. 2021;9(7):771.
31. Moyroud L, Hustache S, Goirand L, Hauzanneau M, Epaulard O. Negative perceptions of hepatitis B vaccination among attendees of an urban free testing center for sexually

transmitted infections in France. Human Vaccines & Immunotherapeutics. 2017;13(5):998-1004.
32. Erdoğan F. Could vaccine hesitancy be a global health threat after pandemics?: A observational study at early phase of COVID-19 pandemic in 2020. 2022.
33. Gilmartin CE, Daley AJ, Leung L. The hepatitis B birth-dose immunisation: Exploring parental refusal. Australian and New Zealand Journal of Obstetrics and Gynaecology. 2020;60(1):93-100.
34. Rozbroj T, Lyons A, Lucke J. Vaccine-hesitant and vaccine-refusing parents’ reflections on the way parenthood changed their attitudes to vaccination. Journal of Community Health. 2020;45(1):63-72.
35. Yu W, Liu D, Zheng J, Liu Y, An Z, Rodewald L, et al. Loss of confidence in vaccines following media reports of infant deaths after hepatitis B vaccination in China. International Journal of Epidemiology. 2016;45(2):441-9.
36. Agrinier N, Le Maréchal M, Fressard L, Verger P, Pulcini C. Discrepancies between general practitioners' vaccination recommendations for their patients and practices for their children. Clinical Microbiology and Infection. 2017;23(5):311-7.
37. Killian M, Detoc M, Berthelot P, Charles R, Gagneux-Brunon A, Lucht F, et al. Vaccine hesitancy among general practitioners: evaluation and comparison of their immunisation practice for themselves, their patients and their children. European Journal of Clinical Microbiology & Infectious Diseases. 2016;35:1837-43.
38. Verger P, Fressard L, Collange F, Gautier A, Jestin C, Launay O, et al. Vaccine hesitancy among general practitioners and its determinants during controversies: a national cross-sectional survey in France. EBioMedicine. 2015;2(8):891-7.
39. Bar-Lev S, Reichman S, Barnett-Itzhaki Z. Prediction of vaccine hesitancy based on social media traffic among Israeli parents using machine learning strategies. Israel journal of health policy research. 2021;10:1-8.
40. Powell GA, Zinszer K, Verma A, Bahk C, Madoff L, Brownstein J, Buckeridge D. Media content about vaccines in the United States and Canada, 2012–2014: An analysis using data from the Vaccine Sentimeter. Vaccine. 2016;34(50):6229-35.
41. Afolabi IB, Aremu AB, Maidoki LA, Atulomah NO. Predictors of Hepatitis B Virus Infection Vaccine Hesitancy Among Pregnant Women Attending Antenatal Care at Lubaga Hospital, Kampala, Uganda. Int J Womens Health. 2022;14:1093-104.
42. Francisco PM, Donalisio MR, Gabriel Fde J, Barros MB. Hepatitis B vaccination in adolescents living in Campinas, São Paulo, Brazil. Rev Bras Epidemiol. 2015;18(3):552-67.
43. de Araújo TM, Souza FO, Pinho PS, Werneck GL. Beliefs and Sociodemographic and Occupational Factors Associated with Vaccine Hesitancy among Health Workers. Vaccines (Basel). 2022;10(12).
44. Silva T, Gomes CS, Carmo ASD, Mendes LL, Rezende EM, Velasquez-Melendez G, Matozinhos FP. Spatial analysis of vaccination against Hepatitis B in pregnant women in an urban Brazilian area. Cien Saude Colet. 2021;26(3):1173-82.
45. Guimarães MNC, Facincani T, Santos SSD. Hepatitis B status in hemodialysis patients. Arq Gastroenterol. 2017;54(4):356-8.
46. Pinto Neto L, Vieira JV, Ronchi NR. Vaccination coverage in a cohort of HIV-infected patients receiving care at an AIDS outpatient clinic in Espírito Santo, Brazil. Braz J Infect Dis. 2017;21(5):515-9.
47. Xiridou M, Adam P, Meiberg A, Visser M, Matser A, de Wit J, Op de Coul E. The impact of the COVID-19 pandemic on hepatitis B virus vaccination and transmission among men who have sex with men: A mathematical modelling study. Vaccine. 2022;40(33):4889-96.

48. Machmud PB, Mikolajczyk R, Gottschick C. Understanding hepatitis B vaccination willingness in the adult population in Indonesia: a survey among outpatient and healthcare workers in community health centers. Journal of Public Health. 2023;31(12):1969-80.
49. Bai X, Chen L, Liu X, Tong Y, Wang L, Zhou M, et al. Adult hepatitis B virus vaccination coverage in China from 2011 to 2021: a systematic review. Vaccines. 2022;10(6):900.
50. Vietnam GSOo. Statistical Yearbook of Vietnam 2023. 2023.
51. Akosionu O, Virnig B, Call KT, Yuan J-M, Chanthanouvong S, Nguyen RH. The impact of gender differences in attitudes and beliefs concerning HBV vaccination and screening in the Lao community. Journal of immigrant and minority health. 2016;18:277-81.
52. Duodu PA, Darkwah E, Agbadi P, Duah HO, Nutor JJ. Prevalence and geo-clinicodemographic factors associated with hepatitis B vaccination among healthcare workers in five developing countries. BMC infectious diseases. 2022;22(1):599.
53. Dangerfeild K. Why Women Are Bearing the Brunt of COVID-19 Vaccine Side Effects.
54. Zhu D, Guo N, Wang J, Nicholas S, Wang Z, Zhang G, et al. Socioeconomic inequality in Hepatitis B vaccination of rural adults in China. Hum Vaccin Immunother. 2018;14(2):464-70.
55. Machmud PB, Führer A, Gottschick C, Mikolajczyk R. Barriers to and facilitators of hepatitis B vaccination among the adult population in Indonesia: a mixed methods study. Vaccines. 2023;11(2):398.
56. Kim TV, Pham TND, Do B, Dao DVB, Nguyen DX, Lee W, et al. Low HBV knowledge is associated with low HBV vaccination uptake in general adult population despite incentivization of HBV vaccination. BMC Infect Dis. 2024;24(1):470.
57. Aber JO, Bongomin F, Opio SL, Ochola E. Hepatitis B vaccine uptake and associated factors among adults of Gulu city in Northern Uganda: A community-based cross-sectional study. Plos one. 2024;19(3):e0297617.
58. Lailatul Barik A, Purwaningtyas RA, Astuti D. The effectiveness of traditional media (leaflet and poster) to promote health in a community setting in the digital era: A systematic review. 2019.
59. Kollamparambil U, Oyenubi A, Nwosu C. Mental distress, COVID19 vaccine distrust and vaccine hesitancy in South Africa: A causal mediation regression analysis. PLOS ONE. 2023;18(3):e0278218.
60. Yörük S, Türkmen H, Durgut A, Erbek M. Vaccine mistrust among family healthcare professionals and vaccine hesitancy in the communities they serve in Turkey in 2019: a cross-sectional study. Human Vaccines & Immunotherapeutics. 2020;16(12):3155-62.
61. Feghaly E, Hanna V, Mohamad NK, Assaf MA, Sebastian J, El Khatib S, et al. Trust in pharmaceuticals and vaccine hesitancy: exploring factors influencing COVID-19 immunization among Lebanese children aged 1 to 11 years. BMC Pediatr. 2023;23(1):570.
62. Yu L, Wang J, Wangen KR, Chen R, Maitland E, Nicholas S. Factors associated with adults' perceived need to vaccinate against hepatitis B in rural China. Human Vaccines & Immunotherapeutics. 2016;12(5):1149-54.
63. Ebrahimi OV, Johnson MS, Ebling S, Amundsen OM, Halsøy Ø, Hoffart A, et al. Risk, Trust, and Flawed Assumptions: Vaccine Hesitancy During the COVID-19 Pandemic. Front Public Health. 2021;9:700213.
64. Huynh HP. Examining four types of anti-vaccination attitudes prior to and during the COVID-19 pandemic. Current Psychology. 2023;42(32):28812-9.
65. Ebrahimi OV, Sandbakken EM, Moss SM, Johnson SU, Hoffart A, Bauermeister S, et al. Modifiable risk factors of vaccine hesitancy: insights from a mixed methods multiple population study combining machine learning and thematic analysis during the COVID-19 pandemic. BMC medicine. 2025;23(1):155.

Willingness to accept the HBV vaccine and related factors: Administering the vaccination attitudes examination scale to Vietnamese adults

Reviewer 1:

Summary of the Study

This cross-sectional study, conducted with 926 adults in Ho Chi Minh City, Vietnam, aimed to investigate the prevalence and correlates of hepatitis B virus (HBV) vaccine hesitancy and refusal. Utilizing a self-administered questionnaire and the validated Vaccination Attitudes Examination (VAX) scale, the researchers assessed participants' sociodemographic characteristics, HBV knowledge, and vaccination intentions. The study successfully validated the four-factor structure of the VAX scale in the Vietnamese context and identified key predictors: vaccine refusal was linked to lower income, poorer knowledge, younger age, and a preference for natural immunity, while hesitancy was associated with lower income, poorer knowledge, and concerns about commercial profiteering. A counterintuitive finding was that higher HBV knowledge was positively correlated with increased mistrust and worries about vaccines. The authors conclude that the VAX scale is a valuable tool for identifying vaccine resistance in Vietnam and that targeted public health communication addressing these specific psychological and socioeconomic factors is crucial to improving HBV vaccination coverage.

Overall Recommendation: Major Revisions

This manuscript addresses a timely and important public health issue in Vietnam. The topic of HBV vaccine hesitancy is significant, and the study's ambition to validate a standardized tool (the VAX scale) while identifying specific predictors of hesitancy and refusal is commendable. The sample size is substantial, and the statistical analyses, including CFA and multinomial logistic regression, are appropriate. However, the manuscript suffers from several critical flaws, including factual impossibilities, internal contradictions, and underdeveloped interpretations of key findings, which preclude publication in its current form. Major revisions are required to address the points detailed below.

Major Comments

1. **Major Internal Contradiction: Vaccine Intention vs. Status.** There is a glaring and irreconcilable contradiction in the reporting of the primary outcome.

- The **Abstract** states: "65.6% (n = 607) refused vaccination, 19.3% (n = 179) were hesitant, and 15.1% (n = 140) accepted the vaccine."
- **Table 1** states the exact opposite for the "Vaccine intentions" category: "Refusal 140 (15.1%), Hesitant 179 (19.3%), Accept 607 (65.6%)." This is a fundamental error that makes interpretation of the results impossible. Which is correct? Furthermore, the manuscript reports that 58.6% of participants were already vaccinated. How can a previously vaccinated person "refuse" or "accept" the vaccine in terms of future intention? The authors must clarify the distinction between "vaccination status" (already vaccinated) and "vaccination intention" (for the unvaccinated), correct the numbers, and ensure the text and tables are perfectly aligned. The entire results and discussion sections may need to be rewritten based on the correct data.

à **Respone to Reviewer:**

The variable VAX reflects the general attitude towards hepatitis B vaccination, encompassing both individuals who have already been vaccinated and those who have not. Therefore, participants who have been vaccinated may still select “accept” or “hesitant,” which represents their stance on vaccination overall—for example, regarding booster doses or recommending vaccination to others.

- Vaccination status:

Vaccinated: 543 participants (58.6%)

Not vaccinated: 383 participants (41.4%)

- Among the unvaccinated group (n = 383):

Intend to vaccinate: 275 participants (71.8%)

Do not intend to vaccinate: 108 participants (28.2%)

- Regarding the VAX variable (attitude towards vaccination) regardless of their vaccination status:

Accept: 607 participants (65.6%)

Hesitant: 179 participants (15.1%)

Refusal: 140 participants (19.3%)

- Our interpretation is as follows:

+ The Refusal group likely includes those who do not intend to vaccinate (108) as well as some vaccinated individuals who might refuse additional doses or are against recommending vaccination to others.

+ The Hesitant group may include vaccinated individuals who have not completed the full vaccine series (e.g., fewer than 3 doses) and are hesitant about completing it.

+ The Accept group comprises those who intend to vaccinate (275) and those already vaccinated.

This distinction avoids confusion between actual vaccination status and future vaccination intention. We will revise the manuscript to clearly differentiate these concepts and ensure consistency between the text and tables.

2. **Underdeveloped Interpretation of Counterintuitive Finding.** The study reports that "Knowledge about the HBV vaccine was positively correlated with mistrust of vaccine benefits ($r = 0.23$, $p < 0.01$), worries about future effects ($r = 0.19$, $p < 0.01$)..." This is a highly unusual and important finding that challenges conventional wisdom. However, the authors do not explore it. Why might more knowledgeable individuals be more mistrustful? The discussion should offer potential explanations. For instance:

- Does the "knowledge" scale actually measure exposure to misinformation or a nuanced understanding of risks and benefits?
- Does increased knowledge lead to a greater awareness of rare side effects or the complexities of vaccine development, thus fostering informed skepticism rather than blind trust?
- Is this an artifact of the specific questions asked? This finding is a potential highlight of the paper but is currently presented without the deep analysis it deserves.

à **Response to Reviewer: Revised in the discussion section, line 402 - 414**

3. **Methodological Limitations and Generalizability.** The study uses convenience sampling in public spaces in Ho Chi Minh City, Vietnam's most populous and economically advanced urban center. The authors acknowledge this but do not sufficiently temper their conclusions. The findings regarding vaccine hesitancy in this specific urban, relatively affluent (compared to rural areas) population may not be generalizable to the rest of Vietnam, especially rural regions where HBV burden and healthcare access may differ significantly. The discussion and conclusion should explicitly state this limitation and avoid framing the findings as representative of the entire country.

à **Response to Reviewer: Revised in the Strengths and limitation section.**

4. **Clarity and Consistency in Reporting Results.** The reporting of the regression results is confusing. For example, the text states, "individuals who considerably mistrusted vaccine benefits were 84.3% less likely to refuse the HBV vaccine, they were 78.8% more likely to be hesitant." This is a complex relationship that needs to be stated with absolute clarity. Furthermore, the AORs reported in the text (e.g., AOR = 0.15 for mistrust predicting refusal) seem to contradict the narrative ("less likely to refuse"). The authors must carefully review all statistical interpretations, ensure the narrative matches the AORs, and present these findings in a way that is easy for the reader to understand. Figure 3 and 4 are helpful but must be consistent with the text.

à **Response to Reviewer: The Discussion section has been revised to provide a clearer explanation of these contrasting effects, lined 549-580. The AORs have been re-checked**

and the narrative has been revised to ensure consistency between the statistical results and their interpretation. For example, the statement regarding mistrust (AOR = 0.15 for refusal) now clearly indicates that individuals with higher mistrust were less likely to refuse the vaccine compared with those with lower mistrust. All other AOR interpretations have also been reviewed for clarity.

Minor Comments

- **Abstract:**
- The standardized loadings are reported as "0.94 to 2.31". Factor loadings above 1.0 are highly unusual in standard CFA and may indicate an error (e.g., a standardized loading from a different model, or a typo). Please verify and correct this value.
  à **Response to Reviewer: Revised in the Abstract, line 42, page 2**

- The abstract must be corrected to reflect the accurate (and non-contradictory) findings for vaccine intention.
  à **Response to Reviewer: Revised in Abstract, line 32-34, page 2**

- **Introduction:**
- The introduction is comprehensive but quite lengthy. It could be condensed to more quickly focus on the specific gap in the Vietnamese context that this study aims to fill.
  à **Response to Reviewer: Revised in Introduction, line 124-132**

- **Methods:**
- **Questionnaire:** The description of the knowledge scale is brief. Providing 1-2 example items would help the reader understand what constitutes "knowledge."
  à **Response to Reviewer: detailed in Table 2., page 14**

- **VAX Scale Scoring:** The text states, "Low scores indicated positive vaccination views." However, the mean scores for all items in Table 2 are above the midpoint of 3.0, suggesting a generally negative attitude in the sample, which seems at odds with the high acceptance rate reported in Table 1 (65.6%). This discrepancy should be addressed.
  à **Response to Reviewer: Thank you for your insightful comment. We have included this explanation in the revised manuscript's discussion section to clarify the relationship between vaccine attitudes and vaccination acceptance in our study context, line 415-425.**

- **Table 1:** There is a minor inconsistency in the income conversion. The table footnote states "$1 = VND24,786," making <$605 the correct cutoff. However, the text states the cutoff is <$592. Please ensure consistency.
  à **Response to Reviewer: Revised in introduction section, line 270, page 13**

- **Results:**
- **Figure 1:** The caption refers to the "Italian VAX scale." This is a clear copy-paste error from another manuscript and must be corrected to "Vietnamese VAX scale."
  à **Response to Reviewer: Revised in result section, line 327, page 17**

- **Figure 2:** The caption is helpful, but the figure itself is not described in the results text. Please integrate a description of what this figure shows into the results section.
  à **Response to Reviewer: Revised in introduction section, line 206, page 10**

- **Discussion:**

- The discussion spends considerable time on the impact of COVID-19. While relevant context, it should be streamlined to focus more on interpreting the *specific* findings of this study, especially the perplexing knowledge-mistrust relationship and the specific predictors identified.
  à **Response to Reviewer: Revised in discussion section, line 422-425, page 21**

- The section "Relationship between demographic characteristics and vaccine hesitancy and refusal" would be stronger if it directly grappled with the contradiction between vaccination status and intention.
  à **Response to Reviewer: Revised the Discussion section to explicitly address the apparent contradiction between vaccination status and vaccination intention, line 520-533, page 26-27.**

- **Conclusion:**
- The conclusion should be revised to reflect the corrected findings and more narrowly tailored recommendations based on the study's (urban) context.
  à **Response to Reviewer: Revised conclusion section, line 628-636, page 31**

  This study has the potential to make a valuable contribution to public health research in Vietnam. However, it requires substantial revision to resolve critical errors, clarify its findings, and deepen its analysis. I would be happy to reconsider the manuscript after these points have been thoroughly addressed.

Reviewer #2: I think they should make a structural summary
In relation to the entire manuscript take into account the following:

A) Results internal consistency & wording
- Intention distribution is reversed in one place. Replace the incorrect sentence with the correct direction (acceptance 65.6%, hesitancy 19.3%, refusal 15.1%).
à **Response to Reviewer: revised Table 1, page 13**

-Interpretation of odds ratios is flipped in several sentences (e.g., healthcare workers; age 18–45). Correct all instances where AOR < 1 is described as an increase.
à **Response to Reviewer: we have corrected 2 instances where AOR < 1 was previously misinterpreted as an increase; these now correctly indicate reduced odds/less likely.**

-'P-value typos / impossible thresholds (e.g., p < 0.00). Use p < 0.01 or the exact p-value.
B) Abstract & methods language
à **Response to Reviewer: revised, line 338, page 18**

-Grammar: "a cross-sectional survey of 926 adults was conducted."
à **Response to Reviewer: revised in abstract, line 31-32, page 2**

Terminology: use "multinomial logistic regression" consistently and specify reference outcome and categories.
à **Response to Reviewer: revised in results section**

C) Psychometrics & figure captions
-Change caption reference from "Italian VAX scale" to "Vietnamese VAX scale."
à **Response to Reviewer: Revised in result section, line 327, page 17**

-Report Cronbach's α and McDonald's ω by subscale and total; group CFA indices (χ^2, CFI, TLI, RMSEA, SRMR) in one sentence.
à **Response to Reviewer: revised in results section, line 317-321, page 17**

-For EFA/parallel analysis, consider polychoric correlations and WLSMV estimation for ordinal Likert data.
à **Response to Reviewer:** Thank you for your valuable methodological recommendation. We fully agree that using polychoric correlations and WLSMV estimation is an excellent approach for EFA/CFA with ordinal Likert-type data. However, in this study, we chose to apply maximum likelihood–based procedures in SPSS primarily to maintain consistency with previous related research in our field and to ensure comparability of results. Future studies will consider implementing polychoric correlations and WLSMV estimation using software such as Mplus or R/lavaan to further enhance methodological rigor.

D) Tables, units, and thresholds
-Standardize the income threshold (e.g., USD $605 or $592) across Abstract, Methods, Tables, and Results, consistent with the exchange rate.
à **Response to Reviewer: Revised**

-Keep variable coding out of the narrative; use readable labels in tables and provide coding details in Supplementary Methods.

à **Response to Reviewer: Revised**

E) Results logic checks
-Maintain consistent interpretation: natural immunity preference → higher refusal; commercial profiteering concerns → higher hesitancy at moderate levels but lower refusal at high levels.
à **Response to Reviewer: Revised**

F) Statistical/reporting upgrades expected by Q1 journals

-Declare the reference outcome (suggest "acceptance"), reference categories, covariate selection strategy (a priori + p-based), multicollinearity checks (e.g., VIF), goodness-of-fit, and sensitivity analyses.
à **Response to Reviewer: Revised in methods section, line 238-244, page 12 and revised in results section, line 300-308, page 16**

-Report effect sizes with 95% CIs and exact p-values (three decimals).
à **Response to Reviewer: revised**

G) References
-Clean the reference list; remove out-of-scope citations (e.g., albumin in cirrhosis; ARDS ventilation). Ensure all references pertain to HBV epidemiology, immunization policy, vaccine hesitancy, or VAX validations. Language & Style Edits (examples)
à **Response to Reviewer: revised**

-Vietnam is one of the countries in East Asia with low hepatitis B vaccination coverage, which poses a serious public-health challenge.
à **Response to Reviewer: revised**

-We conducted a cross-sectional survey of 926 adults…
à **Response to Reviewer: revised**

-We used multinomial logistic regression to estimate adjusted odds ratios (AOR) and 95% confidence intervals for hesitancy and refusal versus acceptance.
à **Response to Reviewer: revised**

- Acceptance was 65.6%, hesitancy 19.3%, and refusal 15.1%.
à **Response to Reviewer: revised, line 274-279, page 13-14**

- Figure 1. CFA results: path diagram and standardized parameters for the Vietnamese VAX scale…
Optional but Valuable Additions
à **Response to Reviewer: revised**

-What this study adds (3 bullets): (i) first psychometric validation of VAX in Vietnamese adults; (ii) actionable correlates of hesitancy/refusal (income, knowledge, natural immunity, commercial concerns); (iii) programmatic targets for adult HBV vaccination.
à **Response to Reviewer: revised, line 549-557, page 28**

-Limitations: convenience sampling (urban), self-report, cross-sectional design (no causality), residual confounding.

à **Response to Reviewer: revised, line 609-626, page 30-31**

- Transparency & reproducibility: brief analysis plan; deposit de-identified dataset and code in OSF/Zenodo with a DOI and link in Data Availability.
à **Response to Reviewer: We have provided the requested information in accordance with the journal's guidelines.**

Reviewer #3: The authors addressed a very important topic that is becoming increasingly worrisome, especially since COVID-19 pandemic to-date; vaccine hesitancy. This is particularly timely as one of the world leaders in, the United States, is fighting its own battle against those trying to break public confidence in the hepatitis B vaccine birth dose.

Abstract:
The authors need to highlight the implication of this original work to public health.
Response to Reviewer: revised in Abstract section, line 48-52, page 3.

Introduction:
The references and data need some updates using most recent publications like the WHO Hepatitis Report 2024 (https://www.who.int/publications/i/item/9789240091672) that reference 2022 data instead of 2019, and Global Health Sector Strategies 2022-2030 (https://www.who.int/teams/global-hiv-hepatitis-and-stis-programmes/strategies/global-health-sector-strategies).
Response to Reviewer: revised in Introduction section, line 57-62, page 3.

There are a few instances where the authors write HBV without "infection" like page 4 – line #6, and line #10.
à **Response to Reviewer: revised in Introduction section, line 70, 73, page 4.**

Page #4 – line# 13, the sentence starting with Innovations in 2021, ….. it is not clear to me whether the authors are referring to innovations in vaccine manufacturing or innovations in vaccination strategies.

It will help the reader to understand access to hepatitis B vaccine to adults if the authors explain how adults can receive their vaccine (if they choose to). Do they need to pay out of pocket, or this is completely covered by the government? This will also come in handy as the authors explain the impact of income.

There seems to be some references missing like on page #4 – line# 13 (stating that hepatitis B vaccine offers nearly 100% protection). The same for page #5, lines #16, 18, and 21 (when referring to vaccine hesitancy has increased since 2009 influenza pandemic.
à **Response to Reviewer: revised in Introduction section, line 74-97, page 4-5.**

Also, on page, the authors reference 2009 influenza pandemic, despite the availability of a wealth of literature on vaccine hesitancy with COVID-19 pandemic and the COVId-19 vaccine. The authors highlight that in the discussion but not in the intro.
à **Response to Reviewer: revised in Introduction section, line 94-99, page 5.**

Despite the fact that VAX scale has been used in many countries and cultures, the authors didn't clarify whether the VAX scale has been used to assess vaccine hesitance for different types of vaccines? It might be helpful to provide this information to the reader to better understand strength of the scale.

à **Response to Reviewer: revised in Introduction section, line 120-123, page 6.**

On page #7, at the end of the introduction, the authors don't really discuss the impact their research will have on public health and on public health programming to address vaccine hesitancy among adults in Vietnam.
à **Response to Reviewer: revised in Introduction section, line 124-132, page 6.**

Methods:
Setting and Research Design:
The first line is missing "and older" after 18 years.
à Response to Reviewer: The phrase "and older" after 18 years is already included in the text, so no revision was necessary.

Questionnaire → Sociodemographic questions: on page #9, can you please explain why the authors have included the question about vaccine intention under the sociodemographic section of the survey?
à Response to Reviewer: Thank you for your question. We included the vaccine intention question in the sociodemographic section because we consider it as an important individual characteristic related to vaccination behavior. Placing it there allows us to treat vaccine intention as an independent variable to analyze its impact on vaccination attitudes measured by the VAX scale.

Data Processing:
- The authors didn't explain how the data entry took place for those surveys that were filled using printed questionnaires.
  à **Response to Reviewer: revised in Method section, line 215-217, page 10-11.**
- The authors also didn't discuss how they handled missing data statistically to make sure that these missing data are not affecting the results.
  à **Response to Reviewer: Thank you for your comment**. We addressed missing data by removing all incomplete responses and entries with missing values before conducting the analysis. Therefore, no imputation methods were applied, and the dataset used for analysis contains only complete cases. This approach ensures that missing data do not affect the results, although it may reduce the sample size.

Statistical Analysis:
There is a missing "f" after (SPSS) for Windows. The "f" is missing.
à **Response to Reviewer: revised, line 222, page 11**
Discussion:
The authors provided much details during the results section on the validity of the VAX scale, and presented swiftly the bi-variate and multivariate analysis results, but they didn't take the time to explain the implications of these results to public health programming in the Vietnamese context.

There are a few missing references from the discussion section, especially when the authors are discussing the socio-economic-political environment in Ho Chi Minh City, and when they were comparing acceptance rates of the city's population with other cities that have similar socio-economic-political environment.

The display of relationship between income and hesitancy – and comparing this to a country like the U.S. may not be the best comparison, where healthcare access is strictly tied to income and the majority of the healthcare system is privately-owned. The situation in Vietnam should be compared to countries with similar healthcare system set-up where the public (governmental) sector plays an important role.

à **Response to Reviewer: Thank you for your valuable feedback. To clarify and avoid any confusion, we have removed the comparison with the U.S. from the discussion.**

On page #22, where the authors are discussing strategies to increase awareness about hepatitis B and the benefits of the vaccine, it would be great if they could add references to studies that have shown that these strategies have worked in similar contexts.
à **Response to Reviewer: revised, line 543, page 27**

On page #23 – line #9, profiteering is missing a "g".
à **Response to Reviewer: revised, line 587, page 29**

Conclusion: The conclusion needs to be tied to the results and the discussion. The authors never highlighted that respondents were asked about the trusted messenger, or their trust in the local health authorities. a conclusion that builds on the study findings will make this manuscript stronger.
à **Response to Reviewer: revised, line 628-636, page 31-32**

Overall: the write-up needs a little strengthening in a few spots, and a good proof-read will identify several typos, missed spacing, and inconsistent font.

Journal Requirements:

1. Please provide additional details regarding participant consent. In the ethics statement in the Methods and online submission information, please ensure that you have specified (1) whether consent was informed and (2) what type you obtained (for instance, written or verbal, and if verbal, how it was documented and witnessed). If your study included minors, state whether you obtained consent from parents or guardians. If the need for consent was waived by the ethics committee, please include this information.

If you are reporting a retrospective study of medical records or archived samples, please ensure that you have discussed whether all data were fully anonymized before you accessed them and/or whether the IRB or ethics committee waived the requirement for informed consent. If patients provided informed written consent to have data from their medical records used in research, please include this information.

2. Please amend your detailed Financial Disclosure statement. This is published with the article. It must therefore be completed in full sentences and contain the exact wording you wish to be published.

i. State the initials, alongside each funding source, of each author to receive each grant. For example: "This work was supported by the National Institutes of Health (####### to AM; ###### to CJ) and the National Science Foundation (###### to AM)."

3. Please ensure that your Ethics Statement is available in its entirety at the beginning of your Methods section, under a subheading 'Ethics Statement'.

4. Please upload a copy of Figure 4 which you refer to in your text on page 17. Or, if the figure is no longer to be included as part of the submission please remove all reference to it within the text.

5. We note that your Data Availability Statement is currently as follows: "All relevant data are within the paper and its Supporting Information files."

Please confirm at this time whether or not your submission contains all raw data required to replicate the results of your study. Authors must share the "minimal data set" for their submission. PLOS defines the minimal data set to consist of the data required to replicate all study findings reported in the article, as well as related metadata and methods (https://journals.plos.org/plosone/s/data-availability#loc-minimal-data-set-definition).

For example, authors should submit the following data:

- The values behind the means, standard deviations and other measures reported;

- The values used to build graphs;

- The points extracted from images for analysis.

Authors do not need to submit their entire data set if only a portion of the data was used in the reported study.

If your submission does not contain these data, please either upload them as Supporting Information files or deposit them to a stable, public repository and provide us with the relevant URLs, DOIs, or accession numbers. For a list of recommended repositories, please see https://journals.plos.org/plosone/s/recommended-repositories.

If there are ethical or legal restrictions on sharing a de-identified data set, please explain them in detail (e.g., data contain potentially sensitive information, data are owned by a third-party organization, etc.) and who has imposed them (e.g., an ethics committee). Please also provide contact information for a data access committee, ethics committee, or other institutional body to which data requests may be sent. If data are owned by a third party, please indicate how others may request data access.

6. Some material included in your submission may be copyrighted. According to PLOS's copyright policy, authors who use figures or other material (e.g., graphics, clipart, maps) from another author or copyright holder must demonstrate or obtain permission to publish this material under the Creative Commons Attribution 4.0 International (CC BY 4.0) License used by PLOS journals. Please closely review the details of PLOS's copyright requirements here: PLOS Licenses and Copyright. If you need to request permissions from a copyright holder, you may use PLOS's Copyright Content Permission form.

Please respond directly to this email or email the journal office and provide any known details concerning your material's license terms and permissions required for reuse, even if you have not yet obtained copyright permissions or are unsure of your material's copyright compatibility.

Potential Copyright Issues:

We do not publish any copyright or trademark symbols that usually accompany proprietary names, eg (R), (C), or TM (e.g. next to drug or reagent names). Therefore please remove all instances of trademark/copyright symbols throughout the text, including 'Google Forms®' on page 8.

If the reviewer comments include a recommendation to cite specific previously published works, please review and evaluate these publications to determine whether they are relevant and should be cited. There is no requirement to cite these works unless the editor has indicated otherwise.

Additional Editor Comments (if provided):
