# Supplementary material for: Willingness to accept the HBV vaccine and related factors: Administering the vaccination attitudes examination scale to Urban Vietnamese adults
Source: PLOS Glob Public Health. 2026 Jul 22;6(7):e0006886. doi: 10.1371/journal.pgph.0006886 (PMC13390869; doi:10.1371/journal.pgph.0006886)
Supplement: Attachment — Submitted filename: PGPH-D-25-02314_reviewer comments_03.27.2026.docx [file pgph.0006886.s010.docx]

The authors addressed many of the previous comments, these are other comments:

**General nots:**

There is still a need for proofreading. I highlighted a few things.

It also seems that the author have removed some of the text that was in the first submission without proper revision to properly connect the text so that it flows smoothly. It feels interrupted.

**Introduction:**

Page #6: The authors removed a big section on global goals of viral hepatitis elimination and the progress to-date. It is their choice of course, but the starting sentence in paragraph two now needs modification.

**Page #8: This is a comment from the first round:** It will help the reader to understand access to hepatitis B vaccine to adults if the authors explain how adults can receive their vaccine (if they choose to). Do they need to pay out of pocket, or this is completely covered by the government? This will also come in handy as the authors explain the impact of income.

**Methods:**

**Questionnaire:**

- **Page #12: missing word:** “The level of negative attitudes toward vaccination across all four VAX domains was *arranged* into three groups:”

**Statistical Analysis:**

- **Page #13: missing data:** The authors addressed outliers but not missing data. How have missing data been handled statistically to make sure that these missing data are not affecting the results.
- Please reference (cite) the statistical packages.

**Discussion:**

The authors took time to explain the public health implications of VAX four-domain results within the Vietnamese context, and they added references to support their interpretation, however, there seems a need for re-organization of flow in the discussion section. The authors provide their interpretation, then repeat the ideas to provide reference from the literature. I recommend re-arranging the text to flow smoothly from one domain (with its supporting reference) to the next.

**Overall:** the write-up still needs a little strengthening in a few spots, and a good proof-read.
