# Supplementary material for: Willingness to accept the HBV vaccine and related factors: Administering the vaccination attitudes examination scale to Urban Vietnamese adults
Source: PLOS Glob Public Health. 2026 Jul 22;6(7):e0006886. doi: 10.1371/journal.pgph.0006886 (PMC13390869; doi:10.1371/journal.pgph.0006886)
Supplement: Attachment — Submitted filename: HBV VACCINE KNOWLEDGE.docx [file pgph.0006886.s005.docx]

**Willingness to accept the HBV vaccine and related factors: Administering the vaccination attitudes examination scale to Vietnamese adults**

**Summary of the Study**

This cross-sectional study, conducted with 926 adults in Ho Chi Minh City, Vietnam, aimed to investigate the prevalence and correlates of hepatitis B virus (HBV) vaccine hesitancy and refusal. Utilizing a self-administered questionnaire and the validated Vaccination Attitudes Examination (VAX) scale, the researchers assessed participants' sociodemographic characteristics, HBV knowledge, and vaccination intentions. The study successfully validated the four-factor structure of the VAX scale in the Vietnamese context and identified key predictors: vaccine refusal was linked to lower income, poorer knowledge, younger age, and a preference for natural immunity, while hesitancy was associated with lower income, poorer knowledge, and concerns about commercial profiteering. A counterintuitive finding was that higher HBV knowledge was positively correlated with increased mistrust and worries about vaccines. The authors conclude that the VAX scale is a valuable tool for identifying vaccine resistance in Vietnam and that targeted public health communication addressing these specific psychological and socioeconomic factors is crucial to improving HBV vaccination coverage.

**Overall Recommendation: Major Revisions**

This manuscript addresses a timely and important public health issue in Vietnam. The topic of HBV vaccine hesitancy is significant, and the study's ambition to validate a standardized tool (the VAX scale) while identifying specific predictors of hesitancy and refusal is commendable. The sample size is substantial, and the statistical analyses, including CFA and multinomial logistic regression, are appropriate. However, the manuscript suffers from several critical flaws, including factual impossibilities, internal contradictions, and underdeveloped interpretations of key findings, which preclude publication in its current form. Major revisions are required to address the points detailed below.

**Major Comments**

1. **Major Internal Contradiction: Vaccine Intention vs. Status.** There is a glaring and irreconcilable contradiction in the reporting of the primary outcome.
   - The **Abstract** states: "65.6% (n = 607) refused vaccination, 19.3% (n = 179) were hesitant, and 15.1% (n = 140) accepted the vaccine."
   - **Table 1** states the exact opposite for the "Vaccine intentions" category: "Refusal 140 (15.1%), Hesitant 179 (19.3%), Accept 607 (65.6%)." This is a fundamental error that makes interpretation of the results impossible. Which is correct? Furthermore, the manuscript reports that 58.6% of participants were already vaccinated. How can a previously vaccinated person "refuse" or "accept" the vaccine in terms of future intention? The authors must clarify the distinction between "vaccination status" (already vaccinated) and "vaccination intention" (for the unvaccinated), correct the numbers, and ensure the text and tables are perfectly aligned. The entire results and discussion sections may need to be rewritten based on the correct data.
2. **Underdeveloped Interpretation of Counterintuitive Finding.** The study reports that "Knowledge about the HBV vaccine was positively correlated with mistrust of vaccine benefits (r = 0.23, p < 0.01), worries about future effects (r = 0.19, p < 0.01)..." This is a highly unusual and important finding that challenges conventional wisdom. However, the authors do not explore it. Why might more knowledgeable individuals be more mistrustful? The discussion should offer potential explanations. For instance:
   - Does the "knowledge" scale actually measure exposure to misinformation or nuanced understanding of risks and benefits?
   - Does increased knowledge lead to a greater awareness of rare side effects or the complexities of vaccine development, thus fostering informed skepticism rather than blind trust?
   - Is this an artifact of the specific questions asked? This finding is a potential highlight of the paper but is currently presented without the deep analysis it deserves.
3. **Methodological Limitations and Generalizability.** The study uses convenience sampling in public spaces in Ho Chi Minh City, Vietnam's most populous and economically advanced urban center. The authors acknowledge this but do not sufficiently temper their conclusions. The findings regarding vaccine hesitancy in this specific urban, relatively affluent (compared to rural areas) population may not be generalizable to the rest of Vietnam, especially rural regions where HBV burden and healthcare access may differ significantly. The discussion and conclusion should explicitly state this limitation and avoid framing the findings as representative of the entire country.
4. **Clarity and Consistency in Reporting Results.** The reporting of the regression results is confusing. For example, the text states, "individuals who considerably mistrusted vaccine benefits were 84.3% less likely to refuse the HBV vaccine, they were 78.8% more likely to be hesitant." This is a complex relationship that needs to be stated with absolute clarity. Furthermore, the AORs reported in the text (e.g., AOR = 0.15 for mistrust predicting refusal) seem to contradict the narrative ("less likely to refuse"). The authors must carefully review all statistical interpretations, ensure the narrative matches the AORs, and present these findings in a way that is easy for the reader to understand. Figure 3 and 4 are helpful but must be consistent with the text.

**Minor Comments**

- **Abstract:**
  - The standardized loadings are reported as "0.94 to 2.31". Factor loadings above 1.0 are highly unusual in standard CFA and may indicate an error (e.g., a standardized loading from a different model, or a typo). Please verify and correct this value.
  - The abstract must be corrected to reflect the accurate (and non-contradictory) findings for vaccine intention.
- **Introduction:**
  - The introduction is comprehensive but quite lengthy. It could be condensed to more quickly focus on the specific gap in the Vietnamese context that this study aims to fill.
- **Methods:**
  - **Questionnaire:** The description of the knowledge scale is brief. Providing 1-2 example items would help the reader understand what constitutes "knowledge."
  - **VAX Scale Scoring:** The text states, "Low scores indicated positive vaccination views." However, the mean scores for all items in Table 2 are above the midpoint of 3.0, suggesting a generally negative attitude in the sample, which seems at odds with the high acceptance rate reported in Table 1 (65.6%). This discrepancy should be addressed.
  - **Table 1:** There is a minor inconsistency in the income conversion. The table footnote states "$1 = VND24,786," making <$605 the correct cutoff. However, the text states the cutoff is <$592. Please ensure consistency.
- **Results:**
  - **Figure 1:** The caption refers to the "Italian VAX scale." This is a clear copy-paste error from another manuscript and must be corrected to "Vietnamese VAX scale."
  - **Figure 2:** The caption is helpful, but the figure itself is not described in the results text. Please integrate a description of what this figure shows into the results section.
- **Discussion:**
  - The discussion spends considerable time on the impact of COVID-19. While relevant context, it should be streamlined to focus more on interpreting the *specific* findings of this study, especially the perplexing knowledge-mistrust relationship and the specific predictors identified.
  - The section "Relationship between demographic characteristics and vaccine hesitancy and refusal" would be stronger if it directly grappled with the contradiction between vaccination status and intention.
- **Conclusion:**
  - The conclusion should be revised to reflect the corrected findings and more narrowly tailored recommendations based on the study's (urban) context.

This study has the potential to make a valuable contribution to public health research in Vietnam. However, it requires substantial revision to resolve critical errors, clarify its findings, and deepen its analysis. I would be happy to reconsider the manuscript after these points have been thoroughly addressed.
