# Supplementary material for: Willingness to accept the HBV vaccine and related factors: Administering the vaccination attitudes examination scale to Urban Vietnamese adults
Source: PLOS Glob Public Health. 2026 Jul 22;6(7):e0006886. doi: 10.1371/journal.pgph.0006886 (PMC13390869; doi:10.1371/journal.pgph.0006886)
Supplement: Attachment — Submitted filename: PGPH-D-25-02314_reviewer comments.docx [file pgph.0006886.s006.docx]

The authors addressed a very important topic that is becoming increasingly worrisome, especially since COVID-19 pandemic to-date; vaccine hesitancy. This is particularly timely as one of the world leaders in, the United States, is fighting its own battle against those trying to break public confidence in the hepatitis B vaccine birth dose.

The data collection method is sound and has been utilized several times, however, some improvements can help make this manuscript stronger.

**Abstract:**

The authors need to highlight the implication of this original work to public health.

**Introduction:**

The references and data need some updates using most recent publications like the WHO Hepatitis Report 2024 (<https://www.who.int/publications/i/item/9789240091672>) that reference 2022 data instead of 2019, and Global Health Sector Strategies 2022-2030 (<https://www.who.int/teams/global-hiv-hepatitis-and-stis-programmes/strategies/global-health-sector-strategies>).

There are a few instances where the authors write HBV without “infection” like page 4 – line #6, and line #10.

Page #4 – line# 13, the sentence starting with Innovations in 2021, ….. it is not clear to me whether the authors are referring to innovations in vaccine manufacturing or innovations in vaccination strategies.

It will help the reader to understand access to hepatitis B vaccine to adults if the authors explain how adults can receive their vaccine (if they choose to). Do they need to pay out of pocket, or this is completely covered by the government? This will also come in handy as the authors explain the impact of income.

There seems to be some references missing like on page #4 – line# 13 (stating that hepatitis B vaccine offers nearly 100% protection). The same for page #5, lines #16, 18, and 21 (when referring to vaccine hesitancy has increased since 2009 influenza pandemic.

Also, on page, the authors reference 2009 influenza pandemic, despite the availability of a wealth of literature on vaccine hesitancy with COVID-19 pandemic and the COVId-19 vaccine. The authors highlight that in the discussion but not in the intro.

Despite the fact that VAX scale has been used in many countries and cultures, the authors didn’t clarify whether the VAX scale has been used to assess vaccine hesitance for different types of vaccines? It might be helpful to provide this information to the reader to better understand strength of the scale.

On page #7, at the end of the introduction, the authors don’t really discuss the impact their research will have on public health and on public health programming to address vaccine hesitancy among adults in Vietnam.

**Methods:**

**Setting and Research Design:**

The first line is missing “and older” after 18 years.

**Questionnaire 🡪 Sociodemographic questions:** on page #9, can you please explain why the authors have included the question about vaccine intention under the sociodemographic section of the survey?

**Data Processing:**

- The authors didn’t explain how the data entry took place for those surveys that were filled using printed questionnaires.
- The authors also didn’t discuss how they handled missing data statistically to make sure that these missing data are not affecting the results.

**Statistical Analysis:**

There is a missing “f” after (SPSS) for Windows. The “f” is missing.

**Discussion:**

The authors provided much details during the results section on the validity of the VAX scale, and presented swiftly the bi-variate and multivariate analysis results, but they didn’t take the time to explain the implications of these results to public health programming in the Vietnamese context.

There are a few missing references from the discussion section, especially when the authors are discussing the socio-economic-political environment in Ho Chi Minh City, and when they were comparing acceptance rates of the city’s population with other cities that have similar socio-economic-political environment.

The display of relationship between income and hesitancy – and comparing this to a country like the U.S. may not be the best comparison, where healthcare access is strictly tied to income and the majority of the healthcare system is privately-owned. The situation in Vietnam should be compared to countries with similar healthcare system set-up where the public (governmental) sector plays an important role.

On page #22, where the authors are discussing strategies to increase awareness about hepatitis B and the benefits of the vaccine, it would be great if they could add references to studies that have shown that these strategies have worked in similar contexts.

On page #23 – line #9, profiteering is missing a “g”.

Conclusion: The conclusion needs to be tied to the results and the discussion. The authors never highlighted that respondents were asked about the trusted messenger, or their trust in the local health authorities. a conclusion that builds on the study findings will make this manuscript stronger.

**Overall:** the write-up needs a little strengthening in a few spots, and a good proof-read will identify several typos, missed spacing, and inconsistent font.
