# Supplementary material for: Willingness to accept the HBV vaccine and related factors: Administering the vaccination attitudes examination scale to Urban Vietnamese adults
Source: PLOS Glob Public Health. 2026 Jul 22;6(7):e0006886. doi: 10.1371/journal.pgph.0006886 (PMC13390869; doi:10.1371/journal.pgph.0006886)
Supplement: Attachment — Submitted filename: Response to Reviewers.docx [file pgph.0006886.s011.docx]

**Willingness to accept the HBV vaccine and related factors: Administering the vaccination attitudes examination scale to Vietnamese adults**

**Reviewer 1:**

**Summary of the Study**

This cross-sectional study, conducted with 926 adults in Ho Chi Minh City, Vietnam, aimed to investigate the prevalence and correlates of hepatitis B virus (HBV) vaccine hesitancy and refusal. Utilizing a self-administered questionnaire and the validated Vaccination Attitudes Examination (VAX) scale, the researchers assessed participants' sociodemographic characteristics, HBV knowledge, and vaccination intentions. The study successfully validated the four-factor structure of the VAX scale in the Vietnamese context and identified key predictors: vaccine refusal was linked to lower income, poorer knowledge, younger age, and a preference for natural immunity, while hesitancy was associated with lower income, poorer knowledge, and concerns about commercial profiteering. A counterintuitive finding was that higher HBV knowledge was positively correlated with increased mistrust and worries about vaccines. The authors conclude that the VAX scale is a valuable tool for identifying vaccine resistance in Vietnam and that targeted public health communication addressing these specific psychological and socioeconomic factors is crucial to improving HBV vaccination coverage.

**Overall Recommendation: Major Revisions**

This manuscript addresses a timely and important public health issue in Vietnam. The topic of HBV vaccine hesitancy is significant, and the study's ambition to validate a standardized tool (the VAX scale) while identifying specific predictors of hesitancy and refusal is commendable. The sample size is substantial, and the statistical analyses, including CFA and multinomial logistic regression, are appropriate. However, the manuscript suffers from several critical flaws, including factual impossibilities, internal contradictions, and underdeveloped interpretations of key findings, which preclude publication in its current form. Major revisions are required to address the points detailed below.

**Major Comments**

1. **Major Internal Contradiction: Vaccine Intention vs. Status.** There is a glaring and irreconcilable contradiction in the reporting of the primary outcome.
   - The **Abstract** states: "65.6% (n = 607) refused vaccination, 19.3% (n = 179) were hesitant, and 15.1% (n = 140) accepted the vaccine."
   - **Table 1** states the exact opposite for the "Vaccine intentions" category: "Refusal 140 (15.1%), Hesitant 179 (19.3%), Accept 607 (65.6%)." This is a fundamental error that makes interpretation of the results impossible. Which is correct? Furthermore, the manuscript reports that 58.6% of participants were already vaccinated. How can a previously vaccinated person "refuse" or "accept" the vaccine in terms of future intention? The authors must clarify the distinction between "vaccination status" (already vaccinated) and "vaccination intention" (for the unvaccinated), correct the numbers, and ensure the text and tables are perfectly aligned. The entire results and discussion sections may need to be rewritten based on the correct data.

à **Respone to Reviewer:**

The variable VAX reflects the general attitude towards hepatitis B vaccination, encompassing both individuals who have already been vaccinated and those who have not. Therefore, participants who have been vaccinated may still select “accept” or “hesitant,” which represents their stance on vaccination overall—for example, regarding booster doses or recommending vaccination to others.

**- Vaccination status:**

Vaccinated: 543 participants (58.6%)

Not vaccinated: 383 participants (41.4%)

**- Among the unvaccinated group (n = 383):**

Intend to vaccinate: 275 participants (71.8%)

Do not intend to vaccinate: 108 participants (28.2%)

**- Regarding the VAX variable (attitude towards vaccination) regardless of their vaccination status:**

Accept: 607 participants (65.6%)

Hesitant: 179 participants (15.1%)

Refusal: 140 participants (19.3%)

**- Our interpretation is as follows:**

+ The Refusal group likely includes those who do not intend to vaccinate (108) as well as some vaccinated individuals who might refuse additional doses or are against recommending vaccination to others.

+ The Hesitant group may include vaccinated individuals who have not completed the full vaccine series (e.g., fewer than 3 doses) and are hesitant about completing it.

+ The Accept group comprises those who intend to vaccinate (275) and those already vaccinated.

This distinction avoids confusion between actual vaccination status and future vaccination intention. We will revise the manuscript to clearly differentiate these concepts and ensure consistency between the text and tables.

2. **Underdeveloped Interpretation of Counterintuitive Finding.** The study reports that "Knowledge about the HBV vaccine was positively correlated with mistrust of vaccine benefits (r = 0.23, p < 0.01), worries about future effects (r = 0.19, p < 0.01)..." This is a highly unusual and important finding that challenges conventional wisdom. However, the authors do not explore it. Why might more knowledgeable individuals be more mistrustful? The discussion should offer potential explanations. For instance:
   - Does the "knowledge" scale actually measure exposure to misinformation or a nuanced understanding of risks and benefits?
   - Does increased knowledge lead to a greater awareness of rare side effects or the complexities of vaccine development, thus fostering informed skepticism rather than blind trust?
   - Is this an artifact of the specific questions asked? This finding is a potential highlight of the paper but is currently presented without the deep analysis it deserves.

à **Response to Reviewer: Revised in the discussion section, line 402 - 414**

3. **Methodological Limitations and Generalizability.** The study uses convenience sampling in public spaces in Ho Chi Minh City, Vietnam's most populous and economically advanced urban center. The authors acknowledge this but do not sufficiently temper their conclusions. The findings regarding vaccine hesitancy in this specific urban, relatively affluent (compared to rural areas) population may not be generalizable to the rest of Vietnam, especially rural regions where HBV burden and healthcare access may differ significantly. The discussion and conclusion should explicitly state this limitation and avoid framing the findings as representative of the entire country.

à **Response to Reviewer: Revised in the Strengths and limitation section.**

4. **Clarity and Consistency in Reporting Results.** The reporting of the regression results is confusing. For example, the text states, "individuals who considerably mistrusted vaccine benefits were 84.3% less likely to refuse the HBV vaccine, they were 78.8% more likely to be hesitant." This is a complex relationship that needs to be stated with absolute clarity. Furthermore, the AORs reported in the text (e.g., AOR = 0.15 for mistrust predicting refusal) seem to contradict the narrative ("less likely to refuse"). The authors must carefully review all statistical interpretations, ensure the narrative matches the AORs, and present these findings in a way that is easy for the reader to understand. Figure 3 and 4 are helpful but must be consistent with the text.

à **Response to Reviewer: The Discussion section has been revised to provide a clearer explanation of these contrasting effects, lined 549-580. The AORs have been re-checked and the narrative has been revised to ensure consistency between the statistical results and their interpretation. For example, the statement regarding mistrust (AOR = 0.15 for refusal) now clearly indicates that individuals with higher mistrust were less likely to refuse the vaccine compared with those with lower mistrust. All other AOR interpretations have also been reviewed for clarity.**

**Minor Comments**

- **Abstract:**
  - The standardized loadings are reported as "0.94 to 2.31". Factor loadings above 1.0 are highly unusual in standard CFA and may indicate an error (e.g., a standardized loading from a different model, or a typo). Please verify and correct this value.

à **Response to Reviewer: Revised in the Abstract, line 42, page 2**

- The abstract must be corrected to reflect the accurate (and non-contradictory) findings for vaccine intention.

à **Response to Reviewer: Revised in Abstract, line 32-34, page 2**

- **Introduction:**
  - The introduction is comprehensive but quite lengthy. It could be condensed to more quickly focus on the specific gap in the Vietnamese context that this study aims to fill.

à **Response to Reviewer: Revised in Introduction, line 124-132**

- **Methods:**
  - **Questionnaire:** The description of the knowledge scale is brief. Providing 1-2 example items would help the reader understand what constitutes "knowledge."

à **Response to Reviewer: detailed in Table 2., page 14**

- **VAX Scale Scoring:** The text states, "Low scores indicated positive vaccination views." However, the mean scores for all items in Table 2 are above the midpoint of 3.0, suggesting a generally negative attitude in the sample, which seems at odds with the high acceptance rate reported in Table 1 (65.6%). This discrepancy should be addressed.

à **Response to Reviewer: Thank you for your insightful comment. We have included this explanation in the revised manuscript’s discussion section to clarify the relationship between vaccine attitudes and vaccination acceptance in our study context, line 415-425.**

- **Table 1:** There is a minor inconsistency in the income conversion. The table footnote states "$1 = VND24,786," making <$605 the correct cutoff. However, the text states the cutoff is <$592. Please ensure consistency.

à **Response to Reviewer: Revised in introduction section, line 270, page 13**

- **Results:**
  - **Figure 1:** The caption refers to the "Italian VAX scale." This is a clear copy-paste error from another manuscript and must be corrected to "Vietnamese VAX scale."

à **Response to Reviewer: Revised in result section, line 327, page 17**

- **Figure 2:** The caption is helpful, but the figure itself is not described in the results text. Please integrate a description of what this figure shows into the results section.

à **Response to Reviewer: Revised in introduction section, line 206, page 10**

- **Discussion:**
  - The discussion spends considerable time on the impact of COVID-19. While relevant context, it should be streamlined to focus more on interpreting the *specific* findings of this study, especially the perplexing knowledge-mistrust relationship and the specific predictors identified.

à **Response to Reviewer: Revised in discussion section, line 422-425, page 21**

- The section "Relationship between demographic characteristics and vaccine hesitancy and refusal" would be stronger if it directly grappled with the contradiction between vaccination status and intention.

à **Response to Reviewer: Revised the Discussion section to explicitly address the apparent contradiction between vaccination status and vaccination intention, line 520-533, page 26-27.**

- **Conclusion:**
  - The conclusion should be revised to reflect the corrected findings and more narrowly tailored recommendations based on the study's (urban) context.

à **Response to Reviewer: Revised conclusion section, line 628-636, page 31**

This study has the potential to make a valuable contribution to public health research in Vietnam. However, it requires substantial revision to resolve critical errors, clarify its findings, and deepen its analysis. I would be happy to reconsider the manuscript after these points have been thoroughly addressed.

**Reviewer #2:** I think they should make a structural summary
In relation to the entire manuscript take into account the following:

A) Results internal consistency & wording

- Intention distribution is reversed in one place. Replace the incorrect sentence with the correct direction (acceptance 65.6%, hesitancy 19.3%, refusal 15.1%).

à **Response to Reviewer: revised Table 1, page 13**

-Interpretation of odds ratios is flipped in several sentences (e.g., healthcare workers; age 18–45). Correct all instances where AOR < 1 is described as an increase.

à **Response to Reviewer: we have corrected 2 instances where AOR < 1 was previously misinterpreted as an increase; these now correctly indicate reduced odds/less likely.**

-'P-value typos / impossible thresholds (e.g., p < 0.00). Use p < 0.01 or the exact p-value.

B) Abstract & methods language

à **Response to Reviewer: revised, line 338, page 18**

-Grammar: “a cross-sectional survey of 926 adults was conducted.”

à **Response to Reviewer: revised in abstract, line 31-32, page 2**

Terminology: use “multinomial logistic regression” consistently and specify reference outcome and categories.

à **Response to Reviewer: revised in results section**

C) Psychometrics & figure captions

-Change caption reference from “Italian VAX scale” to “Vietnamese VAX scale.”

à **Response to Reviewer: Revised in result section, line 327, page 17**

-Report Cronbach’s α and McDonald’s ω by subscale and total; group CFA indices (χ², CFI, TLI, RMSEA, SRMR) in one sentence.

à **Response to Reviewer: revised in results section, line 317-321, page 17**

-For EFA/parallel analysis, consider polychoric correlations and WLSMV estimation for ordinal Likert data.

à **Response to Reviewer:** Thank you for your valuable methodological recommendation. We fully agree that using polychoric correlations and WLSMV estimation is an excellent approach for EFA/CFA with ordinal Likert-type data. However, in this study, we chose to apply maximum likelihood–based procedures in SPSS primarily to maintain consistency with previous related research in our field and to ensure comparability of results. Future studies will consider implementing polychoric correlations and WLSMV estimation using software such as Mplus or R/lavaan to further enhance methodological rigor.

D) Tables, units, and thresholds

-Standardize the income threshold (e.g., USD $605 or $592) across Abstract, Methods, Tables, and Results, consistent with the exchange rate.

à **Response to Reviewer: Revised**

-Keep variable coding out of the narrative; use readable labels in tables and provide coding details in Supplementary Methods.

à **Response to Reviewer: Revised**

E) Results logic checks

-Maintain consistent interpretation: natural immunity preference → higher refusal; commercial profiteering concerns → higher hesitancy at moderate levels but lower refusal at high levels.

à **Response to Reviewer: Revised**

F) Statistical/reporting upgrades expected by Q1 journals

-Declare the reference outcome (suggest “acceptance”), reference categories, covariate selection strategy (a priori + p-based), multicollinearity checks (e.g., VIF), goodness-of-fit, and sensitivity analyses.

à **Response to Reviewer: Revised in methods section, line 238-244, page 12 and revised in results section, line 300-308, page 16**

-Report effect sizes with 95% CIs and exact p-values (three decimals).

à **Response to Reviewer: revised**

G) References

-Clean the reference list; remove out-of-scope citations (e.g., albumin in cirrhosis; ARDS ventilation). Ensure all references pertain to HBV epidemiology, immunization policy, vaccine hesitancy, or VAX validations. Language & Style Edits (examples)

à **Response to Reviewer: revised**

-Vietnam is one of the countries in East Asia with low hepatitis B vaccination coverage, which poses a serious public-health challenge.

à **Response to Reviewer: revised**

-We conducted a cross-sectional survey of 926 adults…

à **Response to Reviewer: revised**

-We used multinomial logistic regression to estimate adjusted odds ratios (AOR) and 95% confidence intervals for hesitancy and refusal versus acceptance.

à **Response to Reviewer: revised**

- Acceptance was 65.6%, hesitancy 19.3%, and refusal 15.1%.

à **Response to Reviewer: revised, line 274-279, page 13-14**

- Figure 1. CFA results: path diagram and standardized parameters for the Vietnamese VAX scale…
Optional but Valuable Additions

à **Response to Reviewer: revised**

-What this study adds (3 bullets): (i) first psychometric validation of VAX in Vietnamese adults; (ii) actionable correlates of hesitancy/refusal (income, knowledge, natural immunity, commercial concerns); (iii) programmatic targets for adult HBV vaccination.

à **Response to Reviewer: revised, line 549-557, page 28**

-Limitations: convenience sampling (urban), self-report, cross-sectional design (no causality), residual confounding.

à **Response to Reviewer: revised, line 609-626, page 30-31**

- Transparency & reproducibility: brief analysis plan; deposit de-identified dataset and code in OSF/Zenodo with a DOI and link in Data Availability.

à **Response to Reviewer: We have provided the requested information in accordance with the journal’s guidelines.**

**Reviewer #3:** The authors addressed a very important topic that is becoming increasingly worrisome, especially since COVID-19 pandemic to-date; vaccine hesitancy. This is particularly timely as one of the world leaders in, the United States, is fighting its own battle against those trying to break public confidence in the hepatitis B vaccine birth dose.

**Abstract:**

The authors need to highlight the implication of this original work to public health.

**Response to Reviewer: revised in Abstract section, line 48-52, page 3.**

**Introduction:**

The references and data need some updates using most recent publications like the WHO Hepatitis Report 2024 (<https://www.who.int/publications/i/item/9789240091672>) that reference 2022 data instead of 2019, and Global Health Sector Strategies 2022-2030 (<https://www.who.int/teams/global-hiv-hepatitis-and-stis-programmes/strategies/global-health-sector-strategies>).

**Response to Reviewer: revised in Introduction section, line 57-62, page 3.**

There are a few instances where the authors write HBV without “infection” like page 4 – line #6, and line #10.

à **Response to Reviewer: revised in Introduction section, line 70, 73, page 4.**

Page #4 – line# 13, the sentence starting with Innovations in 2021, ….. it is not clear to me whether the authors are referring to innovations in vaccine manufacturing or innovations in vaccination strategies.

It will help the reader to understand access to hepatitis B vaccine to adults if the authors explain how adults can receive their vaccine (if they choose to). Do they need to pay out of pocket, or this is completely covered by the government? This will also come in handy as the authors explain the impact of income.

There seems to be some references missing like on page #4 – line# 13 (stating that hepatitis B vaccine offers nearly 100% protection). The same for page #5, lines #16, 18, and 21 (when referring to vaccine hesitancy has increased since 2009 influenza pandemic.

à **Response to Reviewer: revised in Introduction section, line 74-97, page 4-5.**

Also, on page, the authors reference 2009 influenza pandemic, despite the availability of a wealth of literature on vaccine hesitancy with COVID-19 pandemic and the COVId-19 vaccine. The authors highlight that in the discussion but not in the intro.

à **Response to Reviewer: revised in Introduction section, line 94-99, page 5.**

Despite the fact that VAX scale has been used in many countries and cultures, the authors didn’t clarify whether the VAX scale has been used to assess vaccine hesitance for different types of vaccines? It might be helpful to provide this information to the reader to better understand strength of the scale.

à **Response to Reviewer: revised in Introduction section, line 120-123, page 6.**

On page #7, at the end of the introduction, the authors don’t really discuss the impact their research will have on public health and on public health programming to address vaccine hesitancy among adults in Vietnam.

à **Response to Reviewer: revised in Introduction section, line 124-132, page 6.**

**Methods:**

**Setting and Research Design:**

The first line is missing “and older” after 18 years.

à Response to Reviewer: The phrase “and older” after 18 years is already included in the text, so no revision was necessary.

**Questionnaire 🡪 Sociodemographic questions:** on page #9, can you please explain why the authors have included the question about vaccine intention under the sociodemographic section of the survey?

à Response to Reviewer: Thank you for your question. We included the vaccine intention question in the sociodemographic section because we consider it as an important individual characteristic related to vaccination behavior. Placing it there allows us to treat vaccine intention as an independent variable to analyze its impact on vaccination attitudes measured by the VAX scale.

**Data Processing:**

- The authors didn’t explain how the data entry took place for those surveys that were filled using printed questionnaires.

à **Response to Reviewer: revised in Method section, line 215-217, page 10-11.**

- The authors also didn’t discuss how they handled missing data statistically to make sure that these missing data are not affecting the results.

à **Response to Reviewer: Thank you for your comment**. We addressed missing data by removing all incomplete responses and entries with missing values before conducting the analysis. Therefore, no imputation methods were applied, and the dataset used for analysis contains only complete cases. This approach ensures that missing data do not affect the results, although it may reduce the sample size.

**Statistical Analysis:**

There is a missing “f” after (SPSS) for Windows. The “f” is missing.

à **Response to Reviewer: revised, line 222, page 11**

**Discussion:**

The authors provided much details during the results section on the validity of the VAX scale, and presented swiftly the bi-variate and multivariate analysis results, but they didn’t take the time to explain the implications of these results to public health programming in the Vietnamese context.

There are a few missing references from the discussion section, especially when the authors are discussing the socio-economic-political environment in Ho Chi Minh City, and when they were comparing acceptance rates of the city’s population with other cities that have similar socio-economic-political environment.

The display of relationship between income and hesitancy – and comparing this to a country like the U.S. may not be the best comparison, where healthcare access is strictly tied to income and the majority of the healthcare system is privately-owned. The situation in Vietnam should be compared to countries with similar healthcare system set-up where the public (governmental) sector plays an important role.

à **Response to Reviewer: Thank you for your valuable feedback. To clarify and avoid any confusion, we have removed the comparison with the U.S. from the discussion.**

On page #22, where the authors are discussing strategies to increase awareness about hepatitis B and the benefits of the vaccine, it would be great if they could add references to studies that have shown that these strategies have worked in similar contexts.

à **Response to Reviewer: revised, line 543, page 27**

On page #23 – line #9, profiteering is missing a “g”.

à **Response to Reviewer: revised, line 587, page 29**

Conclusion: The conclusion needs to be tied to the results and the discussion. The authors never highlighted that respondents were asked about the trusted messenger, or their trust in the local health authorities. a conclusion that builds on the study findings will make this manuscript stronger.

à **Response to Reviewer: revised, line 628-636, page 31-32**

**Overall:** the write-up needs a little strengthening in a few spots, and a good proof-read will identify several typos, missed spacing, and inconsistent font.

**Journal Requirements:**

1. Please provide additional details regarding participant consent. In the ethics statement in the Methods and online submission information, please ensure that you have specified (1) whether consent was informed and (2) what type you obtained (for instance, written or verbal, and if verbal, how it was documented and witnessed). If your study included minors, state whether you obtained consent from parents or guardians. If the need for consent was waived by the ethics committee, please include this information.

If you are reporting a retrospective study of medical records or archived samples, please ensure that you have discussed whether all data were fully anonymized before you accessed them and/or whether the IRB or ethics committee waived the requirement for informed consent. If patients provided informed written consent to have data from their medical records used in research, please include this information.

2. Please amend your detailed Financial Disclosure statement. This is published with the article. It must therefore be completed in full sentences and contain the exact wording you wish to be published.
 
i. State the initials, alongside each funding source, of each author to receive each grant. For example: "This work was supported by the National Institutes of Health (####### to AM; ###### to CJ) and the National Science Foundation (###### to AM)."

3. Please ensure that your Ethics Statement is available in its entirety at the beginning of your Methods section, under a subheading 'Ethics Statement'.

4. Please upload a copy of Figure 4 which you refer to in your text on page 17. Or, if the figure is no longer to be included as part of the submission please remove all reference to it within the text.

5. We note that your Data Availability Statement is currently as follows: “All relevant data are within the paper and its Supporting Information files.”
 
 Please confirm at this time whether or not your submission contains all raw data required to replicate the results of your study. Authors must share the “minimal data set” for their submission. PLOS defines the minimal data set to consist of the data required to replicate all study findings reported in the article, as well as related metadata and methods (<https://journals.plos.org/plosone/s/data-availability#loc-minimal-data-set-definition>).
 
 For example, authors should submit the following data:
 
 - The values behind the means, standard deviations and other measures reported;

 - The values used to build graphs;

 - The points extracted from images for analysis.

Authors do not need to submit their entire data set if only a portion of the data was used in the reported study.

 If your submission does not contain these data, please either upload them as Supporting Information files or deposit them to a stable, public repository and provide us with the relevant URLs, DOIs, or accession numbers. For a list of recommended repositories, please see <https://journals.plos.org/plosone/s/recommended-repositories>.
 
 If there are ethical or legal restrictions on sharing a de-identified data set, please explain them in detail (e.g., data contain potentially sensitive information, data are owned by a third-party organization, etc.) and who has imposed them (e.g., an ethics committee). Please also provide contact information for a data access committee, ethics committee, or other institutional body to which data requests may be sent. If data are owned by a third party, please indicate how others may request data access.

6. Some material included in your submission may be copyrighted. According to PLOS’s copyright policy, authors who use figures or other material (e.g., graphics, clipart, maps) from another author or copyright holder must demonstrate or obtain permission to publish this material under the Creative Commons Attribution 4.0 International (CC BY 4.0) License used by PLOS journals. Please closely review the details of PLOS’s copyright requirements here: PLOS Licenses and Copyright. If you need to request permissions from a copyright holder, you may use PLOS's Copyright Content Permission form.

Please respond directly to this email or email the journal office and provide any known details concerning your material's license terms and permissions required for reuse, even if you have not yet obtained copyright permissions or are unsure of your material's copyright compatibility.

Potential Copyright Issues:

We do not publish any copyright or trademark symbols that usually accompany proprietary names, eg (R), (C), or TM (e.g. next to drug or reagent names). Therefore please remove all instances of trademark/copyright symbols throughout the text, including ‘Google Forms®’ on page 8.

If the reviewer comments include a recommendation to cite specific previously published works, please review and evaluate these publications to determine whether they are relevant and should be cited. There is no requirement to cite these works unless the editor has indicated otherwise. 

Additional Editor Comments (if provided):
