# Supplementary material for: Willingness to accept the HBV vaccine and related factors: Administering the vaccination attitudes examination scale to Urban Vietnamese adults
Source: PLOS Glob Public Health. 2026 Jul 22;6(7):e0006886. doi: 10.1371/journal.pgph.0006886 (PMC13390869; doi:10.1371/journal.pgph.0006886)
Supplement: Attachment — Submitted filename: Response to Reviewers.docx [file pgph.0006886.s013.docx]

**Response to Reviewers**

**Reviewer 1:**

The authors addressed many of the previous comments, these are other comments:

**General nots:**

There is still a need for proofreading. I highlighted a few things.

It also seems that the author have removed some of the text that was in the first submission without proper revision to properly connect the text so that it flows smoothly. It feels interrupted.

**Introduction:**

Page #6: The authors removed a big section on global goals of viral hepatitis elimination and the progress to-date. It is their choice of course, but the starting sentence in paragraph two now needs modification.

Response: We have revised paragraph 2 to reintroduce the global goals for viral hepatitis elimination. The paragraph now begins with the statement: “The WHO Global Health Sector Strategy on Viral Hepatitis aims to eliminate viral hepatitis as a public health threat by 2030, targeting a 90% reduction in incidence and a 65% reduction in mortality [3]. However, achieving these goals remains particularly challenging for developing countries”

**Page #8: This is a comment from the first round:** It will help the reader to understand access to hepatitis B vaccine to adults if the authors explain how adults can receive their vaccine (if they choose to). Do they need to pay out of pocket, or this is completely covered by the government? This will also come in handy as the authors explain the impact of income.

Response: We have added an explanation regarding access to hepatitis B vaccination for adults in the Vietnamese context. “In the Vietnamese context, while the National Expanded Program on Immunization has provided free HBV vaccines for children since 2003, the National Action Plan for 2021–2025 does not prioritize the broader adult population. Consequently, most adults must access vaccination through voluntary services and pay out-of-pocket expenses. This financial requirement makes income a major determinant of vaccine uptake, as individuals with lower economic status may face significant barriers in accessing non-subsidized immunization services.”

**Methods:**

**Questionnaire:**

- **Page #12: missing word:** “The level of negative attitudes toward vaccination across all four VAX domains was *arranged* into three groups:”

Response: We have corrected the missing word in the sentence on page 12

**Statistical Analysis:**

- **Page #13: missing data:** The authors addressed outliers but not missing data. How have missing data been handled statistically to make sure that these missing data are not affecting the results.

Response: We have corrected the missing data in the sentence on page 13

- Please reference (cite) the statistical packages.

Response: We have revised the manuscript to include references to the statistical packages used. “Data were analyzed using IBM Statistical Package for the Social Sciences (SPSS) for Windows, version 26.0 (IBM Corp., Armonk, NY, USA), AMOS version 24.0, and the 'psych' package in R version 4.3.1 (R Foundation for Statistical Computing).”

**Discussion:**

The authors took time to explain the public health implications of VAX four-domain results within the Vietnamese context, and they added references to support their interpretation, however, there seems a need for re-organization of flow in the discussion section. The authors provide their interpretation, then repeat the ideas to provide reference from the literature. I recommend re-arranging the text to flow smoothly from one domain (with its supporting reference) to the next.

**Overall:** the write-up still needs a little strengthening in a few spots, and a good proof-read.

**Reviewer 2:**

1. Contradictions Regarding Predictors of Refusal and Hesitancy (Critical) There are severe discrepancies between the Abstract, the Results section, and the Discussion regarding the direction of the associations between VAX subscales (mistrust, commercial concerns) and vaccine refusal.

• Abstract vs. Results:

o The Abstract states: "Vaccine refusal was linked to... mistrust..."

o The Results section states: "Refusal was lower among the respondents who were substantially concerned about commercial profiteering... but lower among those who mistrusted vaccine benefits (AOR = 0.15)."

o An AOR of 0.15 implies that those with high mistrust are 85% less likely to refuse the vaccine compared to the reference group. This contradicts the abstract and the fundamental premise of the study (that negative attitudes lead to refusal).

• Interpretation of Odds Ratios: The authors also report that individuals with high commercial concerns had decreased refusal (AOR 0.41). It is counter-intuitive that higher mistrust and higher commercial concerns would lead to lower odds of refusal. The authors must double-check the coding of the outcome variable (Reference category: Acceptance) and the predictor variables.

o Did the authors reverse the coding for the "High" vs. "Low" VAX groups in the regression analysis?

o Alternatively, does the "Refusal" group in the regression actually represent "Acceptance"?

• Action: The authors need to perform a rigorous audit of their multinomial logistic regression output, recode variables if necessary to ensure logical consistency (e.g., High Mistrust should logically increase the odds of refusal/hesitancy), and correct the Abstract and Results accordingly.

Response: "We sincerely thank the Reviewer for this critical observation regarding the logical consistency of our findings. Following your suggestion, we performed a rigorous audit of our multinomial logistic regression output and confirmed that the coding was accurate, with 'Acceptance' as the reference category for the outcome and the 'Low VAX score' group as the reference for predictors. We have revised the Abstract, Results (Section 3.4), and Discussion.

2. Generalizability and Sampling Method

• The title claims the study applies to "Vietnamese adults," but the sampling method was strictly convenience-based in "public spaces, such as parks, cafés, and supermarkets" in Ho Chi Minh City (HCMC).

• HCMC is the most developed economic hub in Vietnam. Attitudes toward healthcare and vaccines there are not representative of rural areas, the Mekong Delta, or Northern highlands.

• Action: The title should be modified to reflect the specific population studied (e.g., "Adults in Ho Chi Minh City" or "Urban Vietnamese Adults"). The limitations section must explicitly discuss how urban convenience sampling biases the results.

Response: We sincerely thank Reviewer 2 for the valuable comments and constructive suggestions. We have carefully revised the manuscript accordingly by modifying the title to specify the study population as urban adults in Ho Chi Minh City, and by explicitly discussing the limitations related to convenience sampling and generalizability in the Limitations section.

3. The "Knowledge Paradox"

• The study reports a positive correlation between HBV knowledge and negative attitudes (mistrust, worries about future effects, preference for natural immunity). The authors suggest this is because "individuals who have a deeper acquaintance... develop a stronger awareness of rare adverse effects."

• While "informed skepticism" is a valid phenomenon, the strength of the positive correlation (for mistrust) is notable. The "Knowledge" section of the questionnaire (Items K1-K10) appears to measure factual knowledge.

• Action: The authors should verify the content of the knowledge questions. Do any questions inadvertently reinforce negative myths (e.g., asking about side effects in a leading way)? If the questions are purely factual, the discussion needs to expand significantly on why health education in this population is correlating with increased hesitancy, rather than the standard assumption that knowledge increases compliance.

Response: Regarding the “knowledge paradox,” we have verified that the knowledge questions are purely factual and expanded the Discussion to provide a more detailed explanation of why higher factual knowledge may correlate with increased hesitancy in this population.

4. Internal Consistency and Redundancy

• The Cronbach’s alpha for the VAX scale is reported as 0.963, which is exceptionally high. While this indicates reliability, values above 0.95 often suggest item redundancy, where multiple items are measuring the exact same concept without adding new information.

• Action: The authors should review the inter-item correlations. If redundancy is found, a shorter version of the scale might be more efficient for future public health use.

Response: We sincerely apologize for the confusion caused by a typographical error in our previous submission. We have re-verified our statistical output and confirmed that the **overall Cronbach’s alpha for the VAX scale is 0.833**, not 0.963 as previously stated.

Specific Comments

Abstract

• Inconsistency: As noted above, please align the statement "Vaccine refusal was linked to... mistrust" with the regression results presented later in the text.

Introduction

• Tone: The introduction is well-structured but could benefit from briefly mentioning specific cultural factors in Vietnam that might influence natural immunity preferences (e.g., Traditional Medicine usage).

Response: We thank the reviewer for the insightful suggestion. We have revised the Introduction to briefly include specific cultural factors in Vietnam, such as the widespread use of Traditional Medicine, that may influence public preferences for natural immunity.

Materials and Methods

• Author Order: There is a discrepancy in the author order between the manuscript header ("Order of Authors: Tram Thi Huyen Nguyen, Tung Hoang...") and the title page in the document text ("Nghi Ho Hoc, Tung Hoang..."). Please ensure the author list is consistent and meets the journal's criteria for authorship.

Response: We apologize for the discrepancy in the author order between the manuscript header and title page. We have carefully reviewed and corrected the manuscript and submission metadata to ensure consistency. The final author order is: Nghi Ho Hoc, Tung Hoang, Cong Minh Le, and Tram Thi Huyen Nguyen, reflecting their contributions and meeting the journal’s authorship criteria.

Results

• Table 1: The income categorization cutoff is US$605. It would be helpful to contextually explain what this threshold represents (e.g., minimum wage, average income) to assist international readers in interpreting "low income."

Response: In Vietnam, significant regional income disparities exist. Urban centers like Ho Chi Minh City and Hanoi have notably higher living costs and salaries, with many positions offering monthly wages between 15 million and 21 million VND (approximately $600–$840 USD). In contrast, rural areas, especially for low-skill jobs, typically see average monthly incomes around 5–7 million VND (roughly $200–$300 USD). This gap justifies using a cutoff near $605 USD to distinguish “low income” within the urban context of our study, reflecting the lower bound of typical urban salaries. Such classification helps international readers contextualize income levels relative to Vietnam’s economic landscape and regional wage variations

• Correlations: The text states: "Knowledge... was positively correlated with mistrust... (r = 0.23)." Later, it states: "Employed individuals exhibited increased vaccine hesitancy... negative associations across categories (e.g., mistrust r = –0.11)." Please clarify which variables are being correlated in the second instance to avoid confusion.

Response: Thank you for the valuable comment. We have revised the manuscript to clarify the nuanced role of mistrust in reducing vaccine refusal but increasing hesitancy.

• Regression Interpretation: The results state: "Individuals with a high level of mistrust were 85.3% less likely to refuse the vaccine but 78.8% more likely to exhibit hesitancy." This suggests that mistrust moves people from "Refusal" to "Hesitancy"? This is a nuanced finding. If accurate, it implies that total rejection (refusal) is driven by other factors (like natural immunity preference), while mistrust keeps people in the "hesitant" camp. This is a fascinating insight but requires clearer explanation.

Response: Thank you for the valuable comment. We have revised the manuscript.

Discussion

• Discrepancy: The discussion states: "refusal was lower among... those who mistrusted vaccine benefits." It then attempts to explain why mistrust leads to refusal. This section contradicts the quantitative findings reported in the Results section. The entire discussion regarding predictors needs to be rewritten to match the (corrected) regression outputs.

Response: "We sincerely thank the Reviewer for identifying this critical logical inconsistency. We have performed a rigorous audit of our multinomial logistic regression outputs and confirmed that the coding and results are accurate. The observed AOR of 0.15 for mistrust (F1) in relation to refusal represents a nuanced finding: in the Vietnamese context, high mistrust does not drive outright rejection but instead moves individuals into the 'hesitant' camp (AOR = 1.78). We have entirely rewritten the Discussion section (under the 'Relationship between demographic characteristics...' heading) to align with these corrected interpretations.

• Comparison: The comparison with the Indonesian study (84.5% acceptance) and Chinese study (20.67% necessity) is good. Adding a comparison with recent HBV acceptance studies in similar Southeast Asian contexts (e.g., Thailand or Philippines) would strengthen the regional relevance.

Response: Thank you for the valuable comment. We have revised the manuscript.

Conclusion

• The conclusion is generally appropriate but should be tempered by the sampling limitations. It claims the VAX scale is effective for "Vietnam" generally; rephrase to indicate it is effective for the sampled population (urban adults), pending further validation in rural areas.

Response: Thank you for the valuable comment. We have revised the manuscript.

Conclusion

This manuscript addresses an important topic with a potentially large sample size. However, the contradictions between the abstract and results regarding the direction of associations (particularly for mistrust and commercial concerns) undermine the validity of the findings. A major revision is required to correct the statistical reporting, resolve the "future date" error, and align the title with the sampling scope. Once these inconsistencies are addressed, the paper will make a solid contribution to the literature on vaccine hesitancy in Vietnam.

* We sincerely thank the reviewers for their valuable and insightful comments. Their feedback has greatly helped us improve the quality of our manuscript and has provided us with important learning opportunities. We truly appreciate the time and effort invested in reviewing our work, which has contributed significantly to enhancing this study.
