# Supplementary material for: Willingness to accept the HBV vaccine and related factors: Administering the vaccination attitudes examination scale to Urban Vietnamese adults
Source: PLOS Glob Public Health. 2026 Jul 22;6(7):e0006886. doi: 10.1371/journal.pgph.0006886 (PMC13390869; doi:10.1371/journal.pgph.0006886)
Supplement: Attachment — Submitted filename: Review of Manuscript-willingness.docx [file pgph.0006886.s012.docx]

Review of Manuscript: PGPH-D-25-02314R2

Willingness to accept the HBV vaccine and related factors: Administering the vaccination attitudes examination scale to Vietnamese adults

General Assessment

This manuscript presents a cross-sectional study validating the Vaccination Attitudes Examination (VAX) scale among Vietnamese adults and assessing factors associated with Hepatitis B (HBV) vaccine acceptance. The study addresses a critical public health issue in a region with high HBV burden. The manuscript is generally well-structured, and the inclusion of advanced psychometric testing (CFA, measurement invariance) adds significant value.

However, there are several issues regarding the timeline of the study, the scope of the population (Urban vs. National), and some internal inconsistencies in the statistical interpretation that need to be addressed before publication.

Major Comments

1. Title and Scope of Population The title refers to "Vietnamese adults," and the abstract discusses the "Vietnamese population" generally. However, the sampling method was restricted to Ho Chi Minh City (HCMC).

Recommendation: To accurately reflect the methodology and avoid overgeneralization, the title should be revised to specify the population, e.g., *"Willingness to accept the HBV vaccine among urban Vietnamese adults"* or *"...among adults in Ho Chi Minh City."* The discussion correctly notes that rural validation is needed, so the title should acknowledge this urban limitation.

2. Interpretation of "Knowledge Paradox" vs. Regression Results There is a complex relationship between knowledge, attitudes (VAX score), and behavior (Refusal/Hesitancy) that needs to be clarified to avoid confusing the reader.

- The Paradox: The authors note that knowledge is *positively* correlated with the VAX score (negative attitude). Meaning: Higher knowledge

Higher skepticism/mistrust.

- The Regression: The results state that "Poor knowledge" predicts *refusal* (AOR = 2.42). Meaning: Lower knowledge

Higher refusal.

- Reconciliation: While the authors attempt to explain this as a "knowledge paradox," the connection between these two findings could be sharper.
  - It appears that *poor knowledge* leads to *outright refusal* (perhaps due to lack of perceived need/awareness).
  - Conversely, *higher knowledge* is associated with *skepticism/mistrust* (High VAX), which correlates with *hesitancy* rather than outright refusal.
  - Recommendation: In the Discussion, explicitly articulate that while increasing knowledge reduces outright refusal, it may simultaneously increase skepticism or "critical awareness" that manifests as hesitancy or higher VAX scores. This distinction is vital for public health messaging (education alone may not solve hesitancy).

**Specific Comments & Minor Revisions**

Abstract

- The abstract summarizes the findings well but could be more specific about the "Knowledge paradox" finding in the results section, as this is a unique contribution.
- Ensure the conclusion aligns with the "Urban" limitation.

Methods

- Typo: Under "Statistical analyses," in the description of multinomial logistic regression reference categories, there is a typo: *"for occupation, ohmemaker/retired/unemployed"*. This should be corrected to *"homemaker"*.
- Sampling: The recruitment in public spaces (cafes, parks) is practical but inherently biased toward individuals with leisure time and higher mobility. This should be explicitly stated as a limitation in the Discussion section.

Results

- Regression Interpretation: The text states, *"individuals with a high level of mistrust were 85.3% less likely to refuse the vaccine but 78.8% more likely to exhibit hesitancy."* This is a counter-intuitive and important finding (Mistrust leads to hesitancy, not refusal). Ensure this is highlighted as a key takeaway—mistrust makes people pause, while preference for natural immunity drives them to refuse.

Discussion

- The discussion on the "Knowledge Paradox" is interesting but requires careful wording to avoid implying that ignorance is better. The conclusion should emphasize that while knowledge is necessary to prevent refusal, it must be paired with trust-building to prevent hesitancy.
- Comparison with other countries: The comparisons with Indonesia, China, and Thailand are good context. Ensure the differences in healthcare systems (e.g., Thailand's Universal Coverage Scheme) are linked to the findings regarding income as a barrier in Vietnam.

Conclusion

The manuscript is a strong contribution to the field of vaccine hesitancy in Vietnam. The psychometric validation of the VAX scale is robust. Once the title is adjusted for scope, and the interplay between knowledge and attitudes is clarified, the paper will be suitable for publication.
