# Supplementary material for: Willingness to accept the HBV vaccine and related factors: Administering the vaccination attitudes examination scale to Urban Vietnamese adults
Source: PLOS Glob Public Health. 2026 Jul 22;6(7):e0006886. doi: 10.1371/journal.pgph.0006886 (PMC13390869; doi:10.1371/journal.pgph.0006886)
Supplement: Peer review history

# Peer review: Decision Letter 0

**DOI:** 10.1371/journal.pgph.0006886.r001

**Authors:** Max Carlos Ramírez-Soto

28 Oct 2025

PGPH-D-25-02314

Willingness to accept the HBV vaccine and related factors: Administering the vaccination attitudes examination scale to Vietnamese adults

PLOS Global Public Health

Dear Dr. Nguyen,

Thank you for submitting your manuscript to PLOS Global Public Health. After careful consideration, we feel that it has merit but does not fully meet PLOS Global Public Health’s publication criteria as it currently stands. Therefore, we invite you to submit a revised version of the manuscript that addresses the points raised during the review process.

Please submit your revised manuscript by Dec 12 2025 11:59PM. If you will need more time than this to complete your revisions, please reply to this message or contact the journal office at globalpubhealth@plos.org. When you're ready to submit your revision, log on to https://www.editorialmanager.com/pgph/ and select the 'Submissions Needing Revision' folder to locate your manuscript file.

Please include the following items when submitting your revised manuscript:

- A rebuttal letter that responds to each point raised by the editor and reviewer(s). You should upload this letter as a separate file labeled 'Response to Reviewers'.
- A marked-up copy of your manuscript that highlights changes made to the original version. You should upload this as a separate file labeled 'Revised Manuscript with Track Changes'.
- An unmarked version of your revised paper without tracked changes. You should upload this as a separate file labeled 'Manuscript'.

Guidelines for resubmitting your figure files are available below the reviewer comments at the end of this letter.

We look forward to receiving your revised manuscript.

Kind regards,

Max Carlos Ramírez-Soto, BSc, MPH, PhD, FRSPH, FECMM

Academic Editor

PLOS Global Public Health

Journal Requirements:

1. Please provide additional details regarding participant consent. In the ethics statement in the Methods and online submission information, please ensure that you have specified (1) whether consent was informed and (2) what type you obtained (for instance, written or verbal, and if verbal, how it was documented and witnessed). If your study included minors, state whether you obtained consent from parents or guardians. If the need for consent was waived by the ethics committee, please include this information.

If you are reporting a retrospective study of medical records or archived samples, please ensure that you have discussed whether all data were fully anonymized before you accessed them and/or whether the IRB or ethics committee waived the requirement for informed consent. If patients provided informed written consent to have data from their medical records used in research, please include this information.

2. Please amend your detailed Financial Disclosure statement. This is published with the article. It must therefore be completed in full sentences and contain the exact wording you wish to be published.

i. State the initials, alongside each funding source, of each author to receive each grant. For example: "This work was supported by the National Institutes of Health (####### to AM; ###### to CJ) and the National Science Foundation (###### to AM)."

3. Please ensure that your Ethics Statement is available in its entirety at the beginning of your Methods section, under a subheading 'Ethics Statement'.

4. Please upload a copy of Figure 4 which you refer to in your text on page 17. Or, if the figure is no longer to be included as part of the submission please remove all reference to it within the text.

5. We note that your Data Availability Statement is currently as follows: “All relevant data are within the paper and its Supporting Information files.”

Please confirm at this time whether or not your submission contains all raw data required to replicate the results of your study. Authors must share the “minimal data set” for their submission. PLOS defines the minimal data set to consist of the data required to replicate all study findings reported in the article, as well as related metadata and methods (https://journals.plos.org/plosone/s/data-availability#loc-minimal-data-set-definition).

For example, authors should submit the following data:

- The values behind the means, standard deviations and other measures reported;

- The values used to build graphs;

- The points extracted from images for analysis.

Authors do not need to submit their entire data set if only a portion of the data was used in the reported study.

If your submission does not contain these data, please either upload them as Supporting Information files or deposit them to a stable, public repository and provide us with the relevant URLs, DOIs, or accession numbers. For a list of recommended repositories, please see https://journals.plos.org/plosone/s/recommended-repositories.

If there are ethical or legal restrictions on sharing a de-identified data set, please explain them in detail (e.g., data contain potentially sensitive information, data are owned by a third-party organization, etc.) and who has imposed them (e.g., an ethics committee). Please also provide contact information for a data access committee, ethics committee, or other institutional body to which data requests may be sent. If data are owned by a third party, please indicate how others may request data access.

6. Some material included in your submission may be copyrighted. According to PLOS’s copyright policy, authors who use figures or other material (e.g., graphics, clipart, maps) from another author or copyright holder must demonstrate or obtain permission to publish this material under the Creative Commons Attribution 4.0 International (CC BY 4.0) License used by PLOS journals. Please closely review the details of PLOS’s copyright requirements here: PLOS Licenses and Copyright. If you need to request permissions from a copyright holder, you may use PLOS's Copyright Content Permission form.

Please respond directly to this email or email the journal office and provide any known details concerning your material's license terms and permissions required for reuse, even if you have not yet obtained copyright permissions or are unsure of your material's copyright compatibility.

Potential Copyright Issues:

We do not publish any copyright or trademark symbols that usually accompany proprietary names, eg (R), (C), or TM (e.g. next to drug or reagent names). Therefore please remove all instances of trademark/copyright symbols throughout the text, including ‘Google Forms®’ on page 8.

If the reviewer comments include a recommendation to cite specific previously published works, please review and evaluate these publications to determine whether they are relevant and should be cited. There is no requirement to cite these works unless the editor has indicated otherwise.

Additional Editor Comments (if provided):

[Note: HTML markup is below. Please do not edit.]

Reviewers' comments:

Reviewer's Responses to Questions

**Comments to the Author**

1. Does this manuscript meet PLOS Global Public Health’s publication criteria? Is the manuscript technically sound, and do the data support the conclusions? The manuscript must describe methodologically and ethically rigorous research with conclusions that are appropriately drawn based on the data presented.

Reviewer #1: No

Reviewer #2: Partly

Reviewer #3: Yes

**********

2. Has the statistical analysis been performed appropriately and rigorously?

Reviewer #1: No

Reviewer #2: No

Reviewer #3: Yes

**********

3. Have the authors made all data underlying the findings in their manuscript fully available (please refer to the Data Availability Statement at the start of the manuscript PDF file)?

The PLOS Data policy requires authors to make all data underlying the findings described in their manuscript fully available without restriction, with rare exception. The data should be provided as part of the manuscript or its supporting information, or deposited to a public repository. For example, in addition to summary statistics, the data points behind means, medians and variance measures should be available. If there are restrictions on publicly sharing data—e.g. participant privacy or use of data from a third party—those must be specified.

Reviewer #1: Yes

Reviewer #2: Yes

Reviewer #3: Yes

**********

4. Is the manuscript presented in an intelligible fashion and written in standard English?

PLOS Global Public Health does not copyedit accepted manuscripts, so the language in submitted articles must be clear, correct, and unambiguous. Any typographical or grammatical errors should be corrected at revision, so please note any specific errors here.

Reviewer #1: Yes

Reviewer #2: Yes

Reviewer #3: Yes

**********

5. Review Comments to the Author

Please use the space provided to explain your answers to the questions above. You may also include additional comments for the author, including concerns about dual publication, research ethics, or publication ethics. (Please upload your review as an attachment if it exceeds 20,000 characters)

Reviewer #1: 1. Major Internal Contradiction: Vaccine Intention vs. Status. There is a glaring and irreconcilable contradiction in the reporting of the primary outcome.

• The Abstract states: "65.6% (n = 607) refused vaccination, 19.3% (n = 179) were hesitant, and 15.1% (n = 140) accepted the vaccine."

• Table 1 states the exact opposite for the "Vaccine intentions" category: "Refusal 140 (15.1%), Hesitant 179 (19.3%), Accept 607 (65.6%)." This is a fundamental error that makes interpretation of the results impossible. Which is correct? Furthermore, the manuscript reports that 58.6% of participants were already vaccinated. How can a previously vaccinated person "refuse" or "accept" the vaccine in terms of future intention? The authors must clarify the distinction between "vaccination status" (already vaccinated) and "vaccination intention" (for the unvaccinated), correct the numbers, and ensure the text and tables are perfectly aligned. The entire results and discussion sections may need to be rewritten based on the correct data.

2. Underdeveloped Interpretation of Counterintuitive Finding. The study reports that "Knowledge about the HBV vaccine was positively correlated with mistrust of vaccine benefits (r = 0.23, p < 0.01), worries about future effects (r = 0.19, p < 0.01)..." This is a highly unusual and important finding that challenges conventional wisdom. However, the authors do not explore it. Why might more knowledgeable individuals be more mistrustful? The discussion should offer potential explanations. For instance:

• Does the "knowledge" scale actually measure exposure to misinformation or nuanced understanding of risks and benefits?

• Does increased knowledge lead to a greater awareness of rare side effects or the complexities of vaccine development, thus fostering informed skepticism rather than blind trust?

• Is this an artifact of the specific questions asked? This finding is a potential highlight of the paper but is currently presented without the deep analysis it deserves.

3. Methodological Limitations and Generalizability. The study uses convenience sampling in public spaces in Ho Chi Minh City, Vietnam's most populous and economically advanced urban center. The authors acknowledge this but do not sufficiently temper their conclusions. The findings regarding vaccine hesitancy in this specific urban, relatively affluent (compared to rural areas) population may not be generalizable to the rest of Vietnam, especially rural regions where HBV burden and healthcare access may differ significantly. The discussion and conclusion should explicitly state this limitation and avoid framing the findings as representative of the entire country.

4. Clarity and Consistency in Reporting Results. The reporting of the regression results is confusing. For example, the text states, "individuals who considerably mistrusted vaccine benefits were 84.3% less likely to refuse the HBV vaccine, they were 78.8% more likely to be hesitant." This is a complex relationship that needs to be stated with absolute clarity. Furthermore, the AORs reported in the text (e.g., AOR = 0.15 for mistrust predicting refusal) seem to contradict the narrative ("less likely to refuse"). The authors must carefully review all statistical interpretations, ensure the narrative matches the AORs, and present these findings in a way that is easy for the reader to understand. Figure 3 and 4 are helpful but must be consistent with the text.

Minor Comments

• Abstract:

• The standardized loadings are reported as "0.94 to 2.31". Factor loadings above 1.0 are highly unusual in standard CFA and may indicate an error (e.g., a standardized loading from a different model, or a typo). Please verify and correct this value.

• The abstract must be corrected to reflect the accurate (and non-contradictory) findings for vaccine intention.

• Introduction:

• The introduction is comprehensive but quite lengthy. It could be condensed to more quickly focus on the specific gap in the Vietnamese context that this study aims to fill.

• Methods:

• Questionnaire: The description of the knowledge scale is brief. Providing 1-2 example items would help the reader understand what constitutes "knowledge."

• VAX Scale Scoring: The text states, "Low scores indicated positive vaccination views." However, the mean scores for all items in Table 2 are above the midpoint of 3.0, suggesting a generally negative attitude in the sample, which seems at odds with the high acceptance rate reported in Table 1 (65.6%). This discrepancy should be addressed.

• Table 1: There is a minor inconsistency in the income conversion. The table footnote states "$1 = VND24,786," making <$605 the correct cutoff. However, the text states the cutoff is <$592. Please ensure consistency.

• Results:

• Figure 1: The caption refers to the "Italian VAX scale." This is a clear copy-paste error from another manuscript and must be corrected to "Vietnamese VAX scale."

• Figure 2: The caption is helpful, but the figure itself is not described in the results text. Please integrate a description of what this figure shows into the results section.

• Discussion:

• The discussion spends considerable time on the impact of COVID-19. While relevant context, it should be streamlined to focus more on interpreting the specific findings of this study, especially the perplexing knowledge-mistrust relationship and the specific predictors identified.

• The section "Relationship between demographic characteristics and vaccine hesitancy and refusal" would be stronger if it directly grappled with the contradiction between vaccination status and intention.

• Conclusion:

• The conclusion should be revised to reflect the corrected findings and more narrowly tailored recommendations based on the study's (urban) context.

Reviewer #2: I think they should make a structural summary

In relation to the entire manuscript take into account the following:

A) Results internal consistency & wording

-Intention distribution is reversed in one place. Replace the incorrect sentence with the correct direction (acceptance 65.6%, hesitancy 19.3%, refusal 15.1%).

-Interpretation of odds ratios is flipped in several sentences (e.g., healthcare workers; age 18–45). Correct all instances where AOR < 1 is described as an increase.

-'P-value typos / impossible thresholds (e.g., p < 0.00). Use p < 0.01 or the exact p-value.

B) Abstract & methods language

-Grammar: “a cross-sectional survey of 926 adults was conducted.”

Terminology: use “multinomial logistic regression” consistently and specify reference outcome and categories.

C) Psychometrics & figure captions

-Change caption reference from “Italian VAX scale” to “Vietnamese VAX scale.”

-Report Cronbach’s α and McDonald’s ω by subscale and total; group CFA indices (χ², CFI, TLI, RMSEA, SRMR) in one sentence.

-For EFA/parallel analysis, consider polychoric correlations and WLSMV estimation for ordinal Likert data.

D) Tables, units, and thresholds

-Standardize the income threshold (e.g., USD $605 or $592) across Abstract, Methods, Tables, and Results, consistent with the exchange rate.

-Keep variable coding out of the narrative; use readable labels in tables and provide coding details in Supplementary Methods.

E) Results logic checks

-Maintain consistent interpretation: natural immunity preference → higher refusal; commercial profiteering concerns → higher hesitancy at moderate levels but lower refusal at high levels.

F) Statistical/reporting upgrades expected by Q1 journals

-Declare the reference outcome (suggest “acceptance”), reference categories, covariate selection strategy (a priori + p-based), multicollinearity checks (e.g., VIF), goodness-of-fit, and sensitivity analyses.

-Report effect sizes with 95% CIs and exact p-values (three decimals).

G) References

-Clean the reference list; remove out-of-scope citations (e.g., albumin in cirrhosis; ARDS ventilation). Ensure all references pertain to HBV epidemiology, immunization policy, vaccine hesitancy, or VAX validations.

Language & Style Edits (examples)

-Vietnam is one of the countries in East Asia with low hepatitis B vaccination coverage, which poses a serious public-health challenge.

-We conducted a cross-sectional survey of 926 adults…

-We used multinomial logistic regression to estimate adjusted odds ratios (AOR) and 95% confidence intervals for hesitancy and refusal versus acceptance.

- Acceptance was 65.6%, hesitancy 19.3%, and refusal 15.1%.

- Figure 1. CFA results: path diagram and standardized parameters for the Vietnamese VAX scale…

Optional but Valuable Additions

-What this study adds (3 bullets): (i) first psychometric validation of VAX in Vietnamese adults; (ii) actionable correlates of hesitancy/refusal (income, knowledge, natural immunity, commercial concerns); (iii) programmatic targets for adult HBV vaccination.

-Limitations: convenience sampling (urban), self-report, cross-sectional design (no causality), residual confounding.

- Transparency & reproducibility: brief analysis plan; deposit de-identified dataset and code in OSF/Zenodo with a DOI and link in Data Availability.

Reviewer #3: The authors addressed a very important topic that is becoming increasingly worrisome, especially since COVID-19 pandemic to-date; vaccine hesitancy. This is particularly timely as one of the world leaders in, the United States, is fighting its own battle against those trying to break public confidence in the hepatitis B vaccine birth dose.

The data collection method is sound and has been utilized several times, however, some improvements can help make this manuscript stronger. Please see my comments in the attached document.

**********

6. PLOS authors have the option to publish the peer review history of their article (what does this mean?). If published, this will include your full peer review and any attached files.

**Do you want your identity to be public for this peer review?**  If you choose “no”, your identity will remain anonymous but your review may still be made public.

For information about this choice, including consent withdrawal, please see our Privacy Policy.

Reviewer #1: **Yes:** PATRICK OKOTI ALUORA

Reviewer #2: No

Reviewer #3: **Yes:** Yasmin Ibrahim

**********

[NOTE: If reviewer comments were submitted as an attachment file, they will be attached to this email and accessible via the submission site. Please log into your account, locate the manuscript record, and check for the action link "View Attachments". If this link does not appear, there are no attachment files.]

-->Figure Resubmissions:

While revising your submission, we strongly recommend that you use PLOS’s NAAS tool (https://ngplosjournals.pagemajik.ai/artanalysis) to test your figure files. NAAS can convert your figure files to the TIFF file type and meet basic requirements (such as print size, resolution), or provide you with a report on issues that do not meet our requirements and that NAAS cannot fix.

After uploading your figures to PLOS’s NAAS tool - https://ngplosjournals.pagemajik.ai/artanalysis, NAAS will process the files provided and display the results in the "Uploaded Files" section of the page as the processing is complete. If the uploaded figures meet our requirements (or NAAS is able to fix the files to meet our requirements), the figure will be marked as "fixed" above. If NAAS is unable to fix the files, a red "failed" label will appear above. When NAAS has confirmed that the figure files meet our requirements, please download the file via the download option, and include these NAAS processed figure files when submitting your revised manuscript.

Attachment Submitted filename: HBV VACCINE KNOWLEDGE.docx

Attachment Submitted filename: PGPH-D-25-02314_reviewer comments.docx

# Author comment: Author response to Decision Letter 1

**DOI:** 10.1371/journal.pgph.0006886.r002

9 Dec 2025

Attachment Submitted filename: Response to Reviewers.docx

# Peer review: Decision Letter 1

**DOI:** 10.1371/journal.pgph.0006886.r003

**Authors:** Max Carlos Ramírez-Soto

8 Apr 2026

PGPH-D-25-02314R1

Willingness to accept the HBV vaccine and related factors: Administering the vaccination attitudes examination scale to Vietnamese adults

PLOS Global Public Health

Dear Dr. Nguyen,

Thank you for submitting your manuscript to PLOS Global Public Health. After careful consideration, we feel that it has merit but does not fully meet PLOS Global Public Health’s publication criteria as it currently stands. Therefore, we invite you to submit a revised version of the manuscript that addresses the points raised during the review process.

Please submit your revised manuscript by May 23 2026 11:59PM. If you will need more time than this to complete your revisions, please reply to this message or contact the journal office at globalpubhealth@plos.org. When you're ready to submit your revision, log on to https://www.editorialmanager.com/pgph/ and select the 'Submissions Needing Revision' folder to locate your manuscript file.

Please include the following items when submitting your revised manuscript:

- A letter that responds to each point raised by the editor and reviewer(s). You should upload this letter as a separate file labeled 'Response to Reviewers'.
- A marked-up copy of your manuscript that highlights changes made to the original version. You should upload this as a separate file labeled 'Revised Manuscript with Track Changes'.
- An unmarked version of your revised paper without tracked changes. You should upload this as a separate file labeled 'Manuscript'.

Guidelines for resubmitting your figure files are available below the reviewer comments at the end of this letter.

As the corresponding author, your ORCID iD is verified in the submission system and will appear in the published article. PLOS supports the use of ORCID, and we encourage all coauthors to register for an ORCID iD and use it as well. Please encourage your coauthors to verify their ORCID iD within the submission system before final acceptance, as unverified ORCID iDs will not appear in the published article. *Only*  the individual author can complete the verification step; PLOS staff *cannot*  verify ORCID iDs on behalf of authors.

We look forward to receiving your revised manuscript.

Kind regards,

Max Carlos Ramírez-Soto, BSc, MPH, PhD, FRSPH, FECMM

Academic Editor

PLOS Global Public Health

Journal Requirements:

If the reviewer comments include a recommendation to cite specific previously published works, please review and evaluate these publications to determine whether they are relevant and should be cited. There is no requirement to cite these works unless the editor has indicated otherwise.

Additional Editor Comments (if provided):

The manuscript is of interest and has several strengths; however, substantial revisions are required before it can be considered further. The authors should revise the manuscript in line with the reviewers’ comments.

[Note: HTML markup is below. Please do not edit.]

Reviewers' comments:

Reviewer's Responses to Questions

**Comments to the Author**

1. If the authors have adequately addressed your comments raised in a previous round of review and you feel that this manuscript is now acceptable for publication, you may indicate that here to bypass the “Comments to the Author” section, enter your conflict of interest statement in the “Confidential to Editor” section, and submit your "Accept" recommendation.

Reviewer #1: (No Response)

Reviewer #3: (No Response)

**********

2. Does this manuscript meet PLOS Global Public Health’s publication criteria? Is the manuscript technically sound, and do the data support the conclusions? The manuscript must describe methodologically and ethically rigorous research with conclusions that are appropriately drawn based on the data presented.

Reviewer #1: Partly

Reviewer #3: Yes

**********

3. Has the statistical analysis been performed appropriately and rigorously?

Reviewer #1: No

Reviewer #3: Yes

**********

4. Have the authors made all data underlying the findings in their manuscript fully available (please refer to the Data Availability Statement at the start of the manuscript PDF file)?

The PLOS Data policy requires authors to make all data underlying the findings described in their manuscript fully available without restriction, with rare exception. The data should be provided as part of the manuscript or its supporting information, or deposited to a public repository. For example, in addition to summary statistics, the data points behind means, medians and variance measures should be available. If there are restrictions on publicly sharing data—e.g. participant privacy or use of data from a third party—those must be specified.

Reviewer #1: No

Reviewer #3: (No Response)

**********

5. Is the manuscript presented in an intelligible fashion and written in standard English?

PLOS Global Public Health does not copyedit accepted manuscripts, so the language in submitted articles must be clear, correct, and unambiguous. Any typographical or grammatical errors should be corrected at revision, so please note any specific errors here.

Reviewer #1: Yes

Reviewer #3: (No Response)

**********

6. Review Comments to the Author

Please use the space provided to explain your answers to the questions above. You may also include additional comments for the author, including concerns about dual publication, research ethics, or publication ethics. (Please upload your review as an attachment if it exceeds 20,000 characters)

Reviewer #1: I have attached here my extensive narration of the review

Reviewer #3: The authors addressed many of the previous comments, these are other comments:

General nots:

There is still a need for proofreading. I highlighted a few things.

It also seems that the author have removed some of the text that was in the first submission without proper revision to properly connect the text so that it flows smoothly. It feels interrupted.

Introduction:

Page #6: The authors removed a big section on global goals of viral hepatitis elimination and the progress to-date. It is their choice of course, but the starting sentence in paragraph two now needs modification.

Page #8: This is a comment from the first round: It will help the reader to understand access to hepatitis B vaccine to adults if the authors explain how adults can receive their vaccine (if they choose to). Do they need to pay out of pocket, or this is completely covered by the government? This will also come in handy as the authors explain the impact of income.

Methods:

Questionnaire:

• Page #12: missing word: “The level of negative attitudes toward vaccination across all four VAX domains was arranged into three groups:”

Statistical Analysis:

• Page #13: missing data: The authors addressed outliers but not missing data. How have missing data been handled statistically to make sure that these missing data are not affecting the results.

• Please reference (cite) the statistical packages.

Discussion:

The authors took time to explain the public health implications of VAX four-domain results within the Vietnamese context, and they added references to support their interpretation, however, there seems a need for re-organization of flow in the discussion section. The authors provide their interpretation, then repeat the ideas to provide reference from the literature. I recommend re-arranging the text to flow smoothly from one domain (with its supporting reference) to the next.

Overall: the write-up still needs a little strengthening in a few spots, and a good proof-read.

**********

7. PLOS authors have the option to publish the peer review history of their article (what does this mean?). If published, this will include your full peer review and any attached files.

**Do you want your identity to be public for this peer review?**  If you choose “no”, your identity will remain anonymous but your review may still be made public.

For information about this choice, including consent withdrawal, please see our Privacy Policy.

Reviewer #1: **Yes:** ALUORA P. OKOTI

Reviewer #3: No

**********

[NOTE: If reviewer comments were submitted as an attachment file, they will be attached to this email and accessible via the submission site. Please log into your account, locate the manuscript record, and check for the action link "View Attachments". If this link does not appear, there are no attachment files.]

-->Figure Resubmissions:

While revising your submission, we strongly recommend that you use PLOS’s NAAS tool (https://ngplosjournals.pagemajik.ai/artanalysis) to test your figure files. NAAS can convert your figure files to the TIFF file type and meet basic requirements (such as print size, resolution), or provide you with a report on issues that do not meet our requirements and that NAAS cannot fix.

After uploading your figures to PLOS’s NAAS tool - https://ngplosjournals.pagemajik.ai/artanalysis, NAAS will process the files provided and display the results in the "Uploaded Files" section of the page as the processing is complete. If the uploaded figures meet our requirements (or NAAS is able to fix the files to meet our requirements), the figure will be marked as "fixed" above. If NAAS is unable to fix the files, a red "failed" label will appear above. When NAAS has confirmed that the figure files meet our requirements, please download the file via the download option, and include these NAAS processed figure files when submitting your revised manuscript.

Attachment Submitted filename: Willingness to accept the HBV vaccine and related factors.pdf

Attachment Submitted filename: PGPH-D-25-02314_R1_reviewer_03.18.2026.pdf

Attachment Submitted filename: PGPH-D-25-02314_reviewer comments_03.27.2026.docx

# Author comment: Author response to Decision Letter 2

**DOI:** 10.1371/journal.pgph.0006886.r004

20 Apr 2026

Attachment Submitted filename: Response to Reviewers.docx

# Peer review: Decision Letter 2

**DOI:** 10.1371/journal.pgph.0006886.r005

**Authors:** Xiao Li

22 Jun 2026

PGPH-D-25-02314R2

Willingness to accept the HBV vaccine and related factors: Administering the vaccination attitudes examination scale to Vietnamese adults

PLOS Global Public Health

Dear Dr. Nguyen,

Thank you for submitting your manuscript to PLOS Global Public Health. After careful consideration, we feel that it has merit but does not fully meet PLOS Global Public Health’s publication criteria as it currently stands. Therefore, we invite you to submit a revised version of the manuscript that addresses the points raised during the review process.

==============================

Could you kindly address the additional comments from Reviewer 1, please?

==============================

Please submit your revised manuscript by Jul 22 2026 11:59PM. If you will need more time than this to complete your revisions, please reply to this message or contact the journal office at globalpubhealth@plos.org. When you're ready to submit your revision, log on to https://www.editorialmanager.com/pgph/ and select the 'Submissions Needing Revision' folder to locate your manuscript file.

Please include the following items when submitting your revised manuscript:

- A letter that responds to each point raised by the editor and reviewer(s). You should upload this letter as a separate file labeled 'Response to Reviewers'.
- A marked-up copy of your manuscript that highlights changes made to the original version. You should upload this as a separate file labeled 'Revised Manuscript with Track Changes'.
- An unmarked version of your revised paper without tracked changes. You should upload this as a separate file labeled 'Manuscript'.

Guidelines for resubmitting your figure files are available below the reviewer comments at the end of this letter.

As the corresponding author, your ORCID iD is verified in the submission system and will appear in the published article. PLOS supports the use of ORCID, and we encourage all coauthors to register for an ORCID iD and use it as well. Please encourage your coauthors to verify their ORCID iD within the submission system before final acceptance, as unverified ORCID iDs will not appear in the published article. *Only*  the individual author can complete the verification step; PLOS staff *cannot*  verify ORCID iDs on behalf of authors.

We look forward to receiving your revised manuscript.

Kind regards,

Xiao Li

Academic Editor

PLOS Global Public Health

**Journal Requirements:**

If the reviewer comments include a recommendation to cite specific previously published works, please review and evaluate these publications to determine whether they are relevant and should be cited. There is no requirement to cite these works unless the editor has indicated otherwise.

Please review your reference list to ensure that it is complete and correct. If you have cited papers that have been retracted, please include the rationale for doing so in the manuscript text, or remove these references and replace them with relevant current references. Any changes to the reference list should be mentioned in the rebuttal letter that accompanies your revised manuscript. If you need to cite a retracted article, indicate the article’s retracted status in the References list and also include a citation and full reference for the retraction notice.

[Note: HTML markup is below. Please do not edit.]

Reviewers' comments:

Reviewer's Responses to Questions

**Comments to the Author**

1. If the authors have adequately addressed your comments raised in a previous round of review and you feel that this manuscript is now acceptable for publication, you may indicate that here to bypass the “Comments to the Author” section, enter your conflict of interest statement in the “Confidential to Editor” section, and submit your "Accept" recommendation.

Reviewer #1: All comments have been addressed

Reviewer #3: All comments have been addressed

**********

2. Does this manuscript meet PLOS Global Public Health’s publication criteria? Is the manuscript technically sound, and do the data support the conclusions? The manuscript must describe methodologically and ethically rigorous research with conclusions that are appropriately drawn based on the data presented.

Reviewer #1: Yes

Reviewer #3: Yes

**********

3. Has the statistical analysis been performed appropriately and rigorously?

Reviewer #1: Yes

Reviewer #3: Yes

**********

4. Have the authors made all data underlying the findings in their manuscript fully available (please refer to the Data Availability Statement at the start of the manuscript PDF file)?

The PLOS Data policy requires authors to make all data underlying the findings described in their manuscript fully available without restriction, with rare exception. The data should be provided as part of the manuscript or its supporting information, or deposited to a public repository. For example, in addition to summary statistics, the data points behind means, medians and variance measures should be available. If there are restrictions on publicly sharing data—e.g. participant privacy or use of data from a third party—those must be specified.

Reviewer #1: Yes

Reviewer #3: Yes

**********

5. Is the manuscript presented in an intelligible fashion and written in standard English?

PLOS Global Public Health does not copyedit accepted manuscripts, so the language in submitted articles must be clear, correct, and unambiguous. Any typographical or grammatical errors should be corrected at revision, so please note any specific errors here.

Reviewer #1: Yes

Reviewer #3: Yes

**********

6. Review Comments to the Author

Please use the space provided to explain your answers to the questions above. You may also include additional comments for the author, including concerns about dual publication, research ethics, or publication ethics. (Please upload your review as an attachment if it exceeds 20,000 characters)

**Reviewer #1:**  1. Title and Scope of Population The title refers to "Vietnamese adults," and the abstract discusses the "Vietnamese population" generally. However, the sampling method was restricted to Ho Chi Minh City (HCMC).

Recommendation: To accurately reflect the methodology and avoid overgeneralization, the title should be revised to specify the population, e.g., "Willingness to accept the HBV vaccine among urban Vietnamese adults" or "...among adults in Ho Chi Minh City." The discussion correctly notes that rural validation is needed, so the title should acknowledge this urban limitation.

2. Interpretation of "Knowledge Paradox" vs. Regression Results There is a complex relationship between knowledge, attitudes (VAX score), and behavior (Refusal/Hesitancy) that needs to be clarified to avoid confusing the reader.

• The Paradox: The authors note that knowledge is positively correlated with the VAX score (negative attitude). Meaning: Higher knowledge

Higher skepticism/mistrust.

• The Regression: The results state that "Poor knowledge" predicts refusal (AOR = 2.42). Meaning: Lower knowledge

Higher refusal.

• Reconciliation: While the authors attempt to explain this as a "knowledge paradox," the connection between these two findings could be sharper.

o It appears that poor knowledge leads to outright refusal (perhaps due to lack of perceived need/awareness).

o Conversely, higher knowledge is associated with skepticism/mistrust (High VAX), which correlates with hesitancy rather than outright refusal.

o Recommendation: In the Discussion, explicitly articulate that while increasing knowledge reduces outright refusal, it may simultaneously increase skepticism or "critical awareness" that manifests as hesitancy or higher VAX scores. This distinction is vital for public health messaging (education alone may not solve hesitancy).

Specific Comments & Minor Revisions

Abstract

• The abstract summarizes the findings well but could be more specific about the "Knowledge paradox" finding in the results section, as this is a unique contribution.

• Ensure the conclusion aligns with the "Urban" limitation.

Methods

• Typo: Under "Statistical analyses," in the description of multinomial logistic regression reference categories, there is a typo: "for occupation, ohmemaker/retired/unemployed". This should be corrected to "homemaker".

• Sampling: The recruitment in public spaces (cafes, parks) is practical but inherently biased toward individuals with leisure time and higher mobility. This should be explicitly stated as a limitation in the Discussion section.

Results

• Regression Interpretation: The text states, "individuals with a high level of mistrust were 85.3% less likely to refuse the vaccine but 78.8% more likely to exhibit hesitancy." This is a counter-intuitive and important finding (Mistrust leads to hesitancy, not refusal). Ensure this is highlighted as a key takeaway—mistrust makes people pause, while preference for natural immunity drives them to refuse.

Discussion

• The discussion on the "Knowledge Paradox" is interesting but requires careful wording to avoid implying that ignorance is better. The conclusion should emphasize that while knowledge is necessary to prevent refusal, it must be paired with trust-building to prevent hesitancy.

• Comparison with other countries: The comparisons with Indonesia, China, and Thailand are good context. Ensure the differences in healthcare systems (e.g., Thailand's Universal Coverage Scheme) are linked to the findings regarding income as a barrier in Vietnam.

**Reviewer #3:**  I’d like to thank the authors for addressing most of my previous comments, the paper is strengthened and looks great. This is a much-needed knowledge.

**********

7. PLOS authors have the option to publish the peer review history of their article (what does this mean?). If published, this will include your full peer review and any attached files.

**Do you want your identity to be public for this peer review?**  If you choose “no”, your identity will remain anonymous but your review may still be made public.

For information about this choice, including consent withdrawal, please see our Privacy Policy.

Reviewer #1: **Yes:** PATRICK OKOTI ALUORA

Reviewer #3: **Yes:** Yasmin Ibrahim

**********

[NOTE: If reviewer comments were submitted as an attachment file, they will be attached to this email and accessible via the submission site. Please log into your account, locate the manuscript record, and check for the action link "View Attachments". If this link does not appear, there are no attachment files.]

**Figure Resubmissions:**

While revising your submission, we strongly recommend that you use PLOS’s NAAS tool (https://ngplosjournals.pagemajik.ai/artanalysis) to test your figure files. NAAS can convert your figure files to the TIFF file type and meet basic requirements (such as print size, resolution), or provide you with a report on issues that do not meet our requirements and that NAAS cannot fix.

After uploading your figures to PLOS’s NAAS tool - https://ngplosjournals.pagemajik.ai/artanalysis, NAAS will process the files provided and display the results in the "Uploaded Files" section of the page as the processing is complete. If the uploaded figures meet our requirements (or NAAS is able to fix the files to meet our requirements), the figure will be marked as "fixed" above. If NAAS is unable to fix the files, a red "failed" label will appear above. When NAAS has confirmed that the figure files meet our requirements, please download the file via the download option, and include these NAAS processed figure files when submitting your revised manuscript.

Attachment Submitted filename: Review of Manuscript-willingness.docx

# Author comment: Author response to Decision Letter 3

**DOI:** 10.1371/journal.pgph.0006886.r006

29 Jun 2026

Attachment Submitted filename: Response_to_Reviewers_auresp_3.docx

# Editor report: Decision Letter 3

**DOI:** 10.1371/journal.pgph.0006886.r007

**Authors:** Xiao Li

1 Jul 2026

Willingness to accept the HBV vaccine and related factors: Administering the vaccination attitudes examination scale to Vietnamese adults

PGPH-D-25-02314R3

Dear Mrs Nguyen,

We are pleased to inform you that your manuscript 'Willingness to accept the HBV vaccine and related factors: Administering the vaccination attitudes examination scale to Vietnamese adults' has been provisionally accepted for publication in PLOS Global Public Health.

Before your manuscript can be formally accepted you will need to complete some formatting changes, which you will receive in a follow up email. A member of our team will be in touch with a set of requests.

Please note that your manuscript will not be scheduled for publication until you have made the required changes, so a swift response is appreciated.

IMPORTANT: The editorial review process is now complete. PLOS will only permit corrections to spelling, formatting or significant scientific errors from this point onwards. Requests for major changes, or any which affect the scientific understanding of your work, will cause delays to the publication date of your manuscript.

If your institution or institutions have a press office, please notify them about your upcoming paper to help maximize its impact. If they'll be preparing press materials, please inform our press team as soon as possible -- no later than 48 hours after receiving the formal acceptance. Your manuscript will remain under strict press embargo until 2 pm Eastern Time on the date of publication. For more information, please contact globalpubhealth@plos.org.

Thank you again for supporting Open Access publishing; we are looking forward to publishing your work in PLOS Global Public Health.

Best regards,

Xiao Li

Academic Editor

PLOS Global Public Health

***********************************************************

Dear authors,

Thank you for the point-by-point response letter!

Please prepare high-quality resolution pictures for the Figures before the production of the article.

Best regards,

Xiao

Reviewer Comments (if any, and for reference):
